# The Impact on Systematic Reviews of Risk of Bias Assessment Changes From Conference Abstracts to Full Text

**DOI:** 10.1002/cesm.70078

**Published:** 2026-03-27

**Authors:** Ryan P. W. Kenny, Katie Twentyman, Dawn Craig, Nick Meader, Gill Norman

**Affiliations:** ^1^ Evidence Synthesis Group, Population Health Sciences Institute, Faculty of Medical Sciences Newcastle University Newcastle upon Tyne UK; ^2^ NIHR Innovation Observatory, Population Health Sciences Institute, Faculty of Medical Sciences Newcastle University Newcastle upon Tyne UK

**Keywords:** gray literature, randomized controlled trials, risk of bias, systematic review

## Abstract

Conference abstracts are commonly included in systematic reviews of evidence. Due to limitations in word count, conference abstracts often lack data or information. This causes issues for the assessment of risk of bias (RoB). We therefore aimed to compare the RoB rating, using the Cochrane RoB tool, for abstracts and full texts. This was accomplished using previously published Cochrane reviews and comparing RoB ratings for included studies originally included as an abstract and later as full text. To accomplish this, we searched the Cochrane Database of Systematic Reviews for reviews with updates across numerous disciplines (depression, anxiety, surgical, Parkinson's disease, Alzheimer's disease, multiple sclerosis, motor neuron disease, cancer, cardiovascular disease, and musculoskeletal disease). We identified 29 reviews, with 52 randomized controlled trials included, which had an abstract and subsequent full text available. If abstracts and full texts were not assessed using the Cochrane RoB tool, we obtained the texts and performed the assessment (*n* = 32). To assess the likelihood of changing the domain assessment rating (low, unclear, or high) from conference abstract to full text, we performed a Bayesian categorical multinomial model for each domain (i.e., signaling question) of the Cochrane tool. At the abstract assessment stage, the most common decision was unclear. Using unclear as the reference level in the model led to increased odds of being rated high at full text, compared to abstract assessment, for domains 2 (allocation concealment: odds ratio [OR] = 3.09, 95% credible intervals (CrI) 1.01 to 9.84) and 3 (blinding: OR = 5.09, 95% CrI 1.67 to 16.20). Domain 2 also had odds of being rated low (OR: 2.93, 95% CrI: 1.13 to 7.87). This suggests an impact of changing conference abstract to full text assessments on RoB. The numerous unclear ratings observed at the abstract assessment were usually due to a lack of reporting. While the findings of this study should be interpreted within the context of small numbers, the evidence still suggests that, in some instances, such as allocation concealment and blinding, it is likely that the decision could change based on full‐text assessment. This also has implications for the certainty of the evidence, which is impacted by the RoB assessments, with having abstracts only or full texts available potentially changing the overall certainty. Current RoB tools may not be suitable for assessing conference abstracts.

## 1 Introduction

Systematic reviews aim to gather all empirical evidence available on a specific research question [1]. Included studies can be in many forms, from peer‐reviewed journal articles to gray literature such as policy briefings. The sources included depend on the area of study and the research question, therefore searches are conducted across multiple databases [2]. Supplementary searches are also commonly performed for gray literature, which includes academic sources such as theses and conference papers [3], although many conference proceedings are available in academic journals. When conducting a systematic review, researchers aim to be as comprehensive as possible in identifying, collating, and summarizing [4] all available evidence that meets their eligibility criteria [2].

The evidence is mixed regarding the impact of the inclusion of conference abstracts on meta‐analysis results. There is potential for it to change the direction of effect (i.e., a non‐statistically significant effect could become statistically significant and *vice versa*) [4]. However, work assessing randomized controlled trials (RCTs) in healthcare has suggested that the gray literature effect sizes are lower than those reported in larger published trials [5]. It is also common for conference abstracts not to become peer‐reviewed full texts [6]. These factors can lead to publication bias, especially if such gray literature is not considered in the systematic review.

Conference abstracts are limited by word count, leading to a lack of reporting of all available data or information. Additionally, contracting authors did not always lead to further information. This causes issues for the assessment of risk of bias, where much information can go unreported. For example, one study found that 56 of 90 (62%) conference abstracts were reported to have a high risk of bias due to poor reporting [7], which could have impacted reviewer judgments due to a lack of information. Therefore, even if the effect estimate of a meta‐analysis is not impacted by the inclusion of conference abstracts, if the evidence is rated as high risk of bias, it could impact the certainty of evidence.

The risk of bias tools themselves may also not be appropriate for assessing conference abstracts, where it is common for information to be missing. This can lead to uncertainty in the assessment, with the required information not being reported in the conference abstract, leading to inadequacy in using the risk of bias tool. This can be true even for RCTs, where there are extensive guidelines for reporting (e.g., CONSORT) [8], which are not always adhered to [7]. Additionally, there may be disparities between the guidelines for reporting and the risk of bias tool requirements for reporting to answer the signaling questions. If there were changes between assessments, this could also impact the certainty of evidence (i.e., Grading of Recommendations, Assessment, Development, and Evaluations; GRADE).

The aim of this study was to compare risk of bias ratings, using the Cochrane risk of bias tool, for abstracts and full texts. This is to understand the impact of changing from abstract to full text on the individual domains and which ones may be most susceptible to change. While we could plausibly expect domains to change from abstract to full text, due to increased information availability, to the author's knowledge, no study has attempted to quantify the magnitude and direction of such changes across the individual RoB domains. To accomplish this, we used previously published Cochrane reviews and compared risk of bias ratings for included studies that had initially been based on an abstract, but the primary reference had been replaced by a full text in a subsequent update. Cochrane reviews were chosen because they are highly standardized and commonly updated. Additionally, they also reliably include conference abstracts.

## 2 Materials and Methods

### 2.1 Searching

As we aimed to assess risk of bias changes related to RCTs, we searched the Cochrane Database of Systematic Reviews (CDSR) for updated systematic reviews. To do this, we conducted multiple searches using the following conditions to give a wide breadth of areas assessed (number of records); see Table 1.

**Table 1:** The areas searched on the CENTRAL database and the number of records identified.

Search area	Number of records	Number of included records
Depression	244	4
Anxiety	165	4
Surgery and Wound	109	6
Parkinson's disease	13	1
Alzheimer's disease	27	2
Multiple sclerosis	30	0
Motor neuron disease	18	0
Cancer	408	9
Cardiovascular disease	194	2
Musculoskeletal disease	35	1
Total	1243	29

These areas were used as search terms in the “Title Abstract Keyword” search field, combined with a search string in the DOI field of “pub3 OR pub4 OR pub5 OR pub6 OR pub7 OR pub8 OR pub9 OR pub10 OR pub11.” This allowed us to identify reviews that had been updated at least once. No date restrictions were applied; all searches were conducted in May 2024.

When records exceeded 100, we only assessed the first 100 records, ordered by relevancy. However, in some instances, we assessed all records. For example, as expected, the anxiety search returned many articles (*n* = 47) that were also included in the depression sample. Consequently, all records were assessed for this search.

### 2.2 Screening

One of two reviewers (R.P.W.K. and K.T.) performed single screening on each individual search area. To assess eligibility, the reviewer accessed the full texts of each version available of the review to check if they included any conference abstracts. If there were conference abstracts included, the reviewer then assessed future versions of this review to see if these abstracts became included full texts. We excluded reviews where there were no conference abstracts in the included studies or if none of the included conference abstracts became full texts. Reviews which included conference abstracts as a secondary reference, where a full text was available, were also not included, as, following Cochrane Guidance, they would not have provided a RoB assessment for the abstract.

### 2.3 Data Extraction/Risk of Bias Assessment

For reviews meeting the eligibility criteria, we extracted data into a piloted form. The data included the citation information (e.g., author), date of first review publication, following dates of further publications, original study reference (i.e., the conference abstract reference), the full text reference (and the year of the review if more than one follow‐up review), and the risk of bias tool used. Where risk of bias information was available for both abstracts and full texts, we extracted this information.

Due to the variation in dates when the reviews were published, there were variations in how the risk of bias was assessed. As the risk of bias tool 1 (RoB1) was used in most of the systematic reviews, we extracted the decisions from the included reviews (*n* = 10) for studies reporting this tool directly (*n* = 20). For other included systematic reviews, where the risk of bias assessment was different (e.g., JADAD, allocation concealment only reported), we searched for the conference abstracts and full texts to perform our own RoB1 assessment. RoB1 assessments of both the conference abstracts and full texts were completed blinded by two reviewers (R.P.W.K. and K.T.). All abstracts were assessed initially, followed by the full texts for each included study, where appropriate. Discrepancies between the two reviews were resolved through discussion. If required, a third reviewer's (GN) opinion was sought. The agreement between reviewers (R.P.W.K. and K.T.) per domain was calculated using the cohen.kappa function in the psych package in R. Weighted and unweighted kappas are available in Table S1.

### 2.4 Statistical Analysis

All analyses were performed using R (v4.4.1). We used the Bayesian package *brms* to conduct analyses [9]. Specifically, we performed a categorical multinomial model, with the dependent variable the rating of the RoB1. Both types (i.e., abstract and full text) and domain (i.e., signaling question of the RoB1 tool) were included as fixed effects, with the model specified without an intercept. Each domain was assessed separately (domain 1, domain 2, etc.). This was entered as an interaction term, as we were interested in the impact of abstract vs full text decisions per domain. Study number was included as a random factor. A random slope for study by type was considered; however, this led to poor model convergence and was not taken further. A random slope for study by type was considered; however, this led to poor model convergence and was not taken further. We compared the results of the full model to a null model, which did not include the interaction term. Weak informative priors (i.e., normal(0,1)) were specified. We conducted a range of other values to assess which would be the better fit (i.e., normal(0,2.5); Cauchy(0,1), Cauchy(0,2.5)), results are available in supplementary material 3. Analyses utilized four chains, 5,000 iterations, thin of one, and burn‐in of 1500 iterations. We compared the results of the full model to the null model. Model fit was assessed using the widely applicable information criterion (WAIC). If violated, we utilized the leave one out analysis (LOO), as per recommendations from the brms package (see Supporting Information Material S1). The unclear category acted as the reference category, owing to its common rating at the abstract stage. We then completed further models for each domain with high and low ratings as the reference categories to assess the impact on the results. Results are represented by odds ratios (OR) with 95% credible intervals (CrI). Models were also undertaken to assess the impact of the CONSORT statement. For this, we conducted two models, a simpler model including abstract year (≤ 2008 vs. > 2008) and a complex model including interactions between type, domain, and abstract year.

## 3 Results

We initially identified 1243 reviews, of which 29 were eligible; see Table 1. From these reviews, we identified 91 RCTs that were originally included as an abstract, for which, in subsequent updates, full texts were available. The original reviews were published between 2001 and 2018. Final updates were published between 2008 and 2024. Twenty‐three reviews had more than two updates. Of the 91 studies identified, 20 were directly extracted from the source reviews. Where RoB tools were not consistently used, or the RoB1 tool was not used at all, we sought the abstracts and full texts of these articles. We could locate and access 32 of these abstracts and linked full texts. The final dataset included 52 unique studies with RoB assessments made at both abstract and full‐text levels.

Of the included reviews, the data range for the final publication was 2004 to 2024. The included abstracts ranged from 1999 to 2019, and full texts ranged from 2003 to 2020. For Alzheimer's disease, the two reviews were published in 2013 and 2019, with abstracts available from 2000 to 2003 and full texts from 2003 to 2007. For anxiety, the four reviews were published from 2015 to 2019, with abstracts available from 2003 to 2011 and full texts from 2006 to 2015. For cancer, the nine reviews were published from 2004 to 2023, with abstracts available from 2002 to 2012 and full texts from 2004 to 2016. For cardiovascular disease, the two reviews were published in 2020 and 2024. Abstracts were published from 2014 to 2015, and full texts from 2016 to 2017. For Depression, the four reviews were published between 2008 and 2019. Abstracts were available from 1999 to 2010 and full texts from 2003 to 2014. The musculoskeletal disease review was published in 2023, with the abstract available in 2016 and the full text in 2017. The Parkinson's disease review was published in 2013. Abstracts were published from 2008 to 2010, and full texts from 2011 to 2012. Finally, surgery and wound reviews were published between 2015 and 2023. Abstracts were published from 2002 to 2018, and full texts from 2003 to 2020.

**Figure 1:**
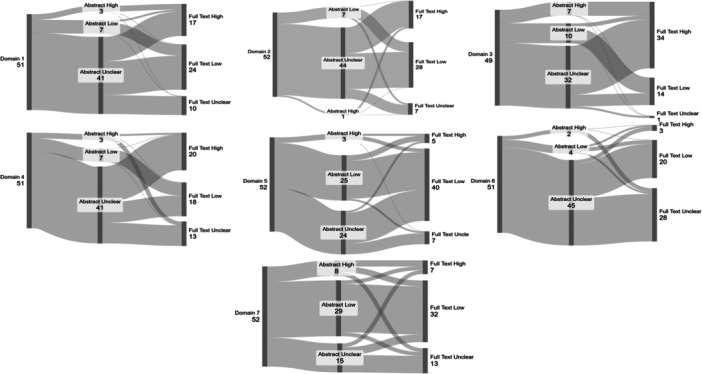
Sankey plots showing changes between abstract and full text risk of bias assessments for each domain.

At the abstract assessment stage, the most common decision for domains 1‐4 and 6 was unclear, while domains 5 and 7 were commonly rated as low. Domain 5 had similar counts of unclear and low (24 vs. 25, respectively; see Table 2). Changes between the abstract and full text RoB assessments can be visualized in Figure 1. The conditional plots for each analysis, plus the trace plots and posterior distribution plots, can be seen in supplementary material 1. Assessments for each domain at abstract and full text can be found in supplementary material 2, as can the full model results for unclear, low, and high reference levels. Matrices for absolute changes from abstract to full text can also be found in supplementary material 3.

**Table 2:** Count data for each domain for abstract and full text decisions.

	Domain 1		Domain 2		Domain 3		Domain 4		Domain 5		Domain 6		Domain 7	
Rating	Abstract	Full text	Abstract	Full text	Abstract	Full text	Abstract	Full text	Abstract	Full text	Abstract	Full text	Abstract	Full text
Unclear	41	11	44	7	32	1	41	13	24	7	45	28	15	13
Low	7	24	7	20	10	14	7	19	25	39	4	20	29	32
High	3	16	1	25	7	34	3	20	3	5	2	3	8	7

*Note:* For domain 1, one study did not report this information. For Domain 3, three studies did not report this information. For domain 4, one study reported a mixed low and high at the abstract. For domain 6, one study did not report this information. These results are not included in the above table and were not included in the analyses.

Domain 1: Random sequence generation

Domain 2: Allocation concealment

Domain 3: Blinding of participants and personnel

Domain 4: Blinding of outcome assessors

Domain 5: Incomplete data

Domain 6: Selective reporting

Domain 7: Other bias

### 3.1 Unclear Reference Results

The multinomial models showed a potential increase in odds for domain 2 for both high ratings at full text (allocation concealment; OR: 3.09, 95% CrI: 1.01 to 9.84) and of a low rating (OR: 2.93, 95% CrI: 1.13 to 7.87). Domain 3 also showed potential for increased odds of a high rating at full text (blinding of participants and personnel; OR: 5.09, 95% CrI: 1.67 to 16.20). There was evidence of lower odds from abstract to full text domain 7 (other bias) to low (OR: 0.38, 95% CrI: 0.16 to 0.94) or high (OR: 0.26, 95% CrI: 0.18 to 0.37). For all other results, the posterior CrIs include 1 (see Table S2). The observed increased in odds associated with domains 2 and 3 (concealment and blinding of participants and personnel) is likely linked to the lack of reporting available for these domains in abstracts. Domain 4 (blinding of outcome assessors) also has a high count of unclear; however, the posterior CrI did included 1 in terms of changing to low or high.

### 3.2 Low Reference Results

The multinominal models showed a potential reduction in odds of changing from low to unclear in domains 2 (allocation concealment; OR: 0.27, 95% CrI: 0.10 to 0.70) and 3 (blinding of participants and personnel). This suggests that once an abstract has attained a low RoB rating, it is unlikely this will change to unclear at full text. There were also increased odds of attaining an unclear rating at full text from abstract in domain 7 (other bias; OR: 3.78, 95% CrI: 1.58 to 8.88). However, this is likely driven by the small number of data points and changes that occur in this domain; only two studies changed from low to unclear ratings between the abstract and full text. For all other analyses, the posterior CrI crossed 1 between abstract and full text from low to high rating (see Table S3).

### 3.3 High Reference Results

The multinominal models showed a reduction in odds of changing from high to unclear in domains 2 (allocation concealment; OR: 0.27, 95% CrI: 0.10 to 0.77) and 3 (blinding of participants and personnel; OR: 0.17, 95% CrI: 0.05 to 0.54). This suggests that once an abstract has attained a high RoB rating, it is unlikely that this will change to unclear at full text. The results suggest domain 7 (other bias) has an increased odds of becoming unclear at full text compared to abstract assessment (OR: 5.49, 95% CrI: 1.92 to 15.31). However, this is likely driven by limited data in this domain. Only two studies were rated high at abstract assessment and then became unclear at full text assessment. This limits potential conclusions that can be made. No further posterior odds changes were observed when using the high rating as a reference, as represented by the posterior CrI including 1 (see Table S4).

### 3.4 Impact on GRADE

Of the 52 included studies, 24 observed an increase in risk of bias across at least two domains. This would have implications for the certainty of evidence. When assessing abstracts, many of the signaling domains were answered as unclear at abstract RoB assessment. This is owing to the limited detail that can be provided in a small word count. When assessing full‐text RoB, it was common for 2 or more domains to become rated as high RoB. Only five assessments at the abstract stage had at least two domains rated high RoB ratings; 13 had at least one domain at risk of bias. When assessing full text, 27 additional assessments had at least two domains rated high RoB (excluding those which had at least two domains rated high RoB at abstract assessment).

Thirty‐one studies were found to have abstracts ranked as low or unclear RoB in two or more domains that were later rated as high RoB when the full text was included. There were five reviews which included abstracts initially and with GRADE information (“very low” *n* = 2, “low to moderate” n = 3). The GRADE rating changed for all three “low to moderate” abstracts became ‘high’ at full text, and the remaining two became “very low to low” at full text. Five other reviews, which included abstracts with no GRADE available at abstract, became “very low to moderate” at full text.

### 3.5 Impact of CONSORT Statement

When using an unclear rating as a reference, the evidence suggested that those abstracts posted after the CONSORT statement (2008) had greater odds of being low. Results were consistent across domain 1 (OR: 1.64, 95% CrI: 1.03 to 2.60), domain 3 (OR: 1.63, 95% CrI: 1.02 to 2.60), domain 4 (OR: 1.65, 95% CrI: 1.04 to 2.64), domain 5 (OR: 1.66, 95% CrI: 1.02 to 2.73), domain 6 (OR: 1.64, 95% CrI: 1.02 to 2.63), and domain 7 (OR: 1.64, 95% CrI: 1.02 to 2.67). Additionally, a numerical trend was also observed for domain 2 (OR: 1.56, 95% CrI: 0.97 to 2.54). Similarly, all domains had lower odds of being rated high risk of bias when >2008, with CrIs not including one. Further results, including those for high and low references, can be seen in supplementary material 3. When considering interactions between type and abstract year, the results became more heterogeneous (see Supporting Information Material 3).

## 4 Discussion

We aimed to assess the potential impact of using the RoB1 tool on abstracts that become full texts. There was limited evidence from the actual reviews we identified, owing to changes over time for the tools used. This meant we had to implement the RoB1 ourselves on studies identified within the systematic reviews that included abstracts, which became full texts in a subsequent update. Overall, we observed that a few of the domains tended to be consistently rated unclear when assessing abstracts, most likely owing to the limited word count. For the most part, there was a change to low or high when assessing the full text, as the word count allowed for greater explanation. For domains 2 and 3, the change to high showed increased odds; however, more data is required to fully determine the impact of assessing abstracts on RoB changes at full text.

Domain 2 is related to allocation concealment, which was often not well described. In this instance, the reviewers undertook the view that if there were a lack of clarity, we would rate this as high at full text. This decision likely affected the rating and the subsequent increased odds. As risk of bias tools are known to be subjective, a different group of assessors may come to different conclusions. We accept that other reviewers may opt for stating unclear still at this stage. A sensitivity analysis was conducted to assess the impact of rating unclear, rather than high, which led to the CrIs including one (see Supporting Information Material 3). Similarly, for domain 3, which is related to blinding, there were inconsistencies in reporting, which led to high risk of bias ratings. However, it is worth noting that domain 3 also had a numerical trend in the odds of becoming low‐rated at full text from abstract. More data may have provided a posterior CrI that did not include one for both low and high assessments. The lack of data is further highlighted by the wide CrIs observed, showing the uncertainty within the model. That said, the evidence reported shows the potential impact of changing from abstract to full text and which domains may be the most susceptible to change. Review authors may therefore wish to pay particular interest to these domains when conducting reviews that include conference abstracts or other sources of limited information.

One reason for the lack of reporting in abstracts for allocation concealment may be due to guidelines also do not include it as part of their criteria. For example, one of the most used guidelines for the reporting of RCTs in abstracts is the CONSORT 2008 guidance [8]. However, one domain in the RoB1 guidance, namely reporting of concealment, is not explicitly mentioned in the abstract guidance. It is also important to state that of the 52 abstracts included in this assessment, 27 pre‐dated these guidelines (i.e., pre‐2008). To this end, we analyzed the impact of the CONSORT statement as a covariate, and the results suggested that those abstracts produced after 2009 were less likely to be rated as high for all domains. This suggests an impact over time; however, when introducing an interaction term by type, the results were more heterogeneous. This, coupled with the relatively small sample size, means that further work is required to fully understand whether the CONSORT guidelines have an impact on risk of bias assessments of conference abstracts.

In the RoB1 tool, the reviewer has three options to rate the signaling question: low, unclear, or high. In the later iteration of the RoB tool, the RoB2, the unclear rating was changed to “some concerns”. This tool is intended to be used to assess individual outcomes of interest. Whilst the RoB1 tool can also be used in this manner, it is commonly used to give an overall indication of potential bias in the studies. We decided to use the RoB1 mainly as it was more commonly used in the literature, as it pre‐dates the RoB2 by ~10 years, therefore far more data on risk of bias ratings were available for this tool. In addition, the Cochrane RoB1 tool is still widely used even in Cochrane reviews [10], and from our previous editorial experience working with Cochrane Review Groups, it was unusual for an update of a review to adopt RoB2 when the previous version had used RoB1.

There was also an issue in the sourcing of abstracts with linked full texts. Twenty studies were included with the publishing authors' decisions at both stages of RoB assessment. We could access a further 32 studies, which meant we had RoB assessments of 52 of 91 (57%) identified studies. Issues with locating abstracts were the main reason for not including (*n* = 31 of 39; 80%). The remaining studies we could not access the full text. This highlights a potential issue for including conference abstracts in reviews. We did not attempt to contact the authors for this work. A number of the publications were also over 20 years prior to this work. Potentially, newer conference abstracts may be easier to access due to online portals.

The difference between assessing full texts and abstracts of RoB potentially has further implications for GRADE. The impact of changing between the two could have a significant impact on the certainty of evidence and, therefore, the overall findings of the review. It is especially important to consider the timeframe between which a conference abstract is published and a full text may become available. Authors may wish to err on the side of caution when interpreting RoB assessments of conference abstracts, when considering GRADE. Further work should be conducted with the consideration of how assessing abstracts only impacts GRADE assessments and conclusions of systematic reviews. This will also be influenced by the number of conference abstracts included in the work; here, we had multiple reviews, which only contributed a single study with a conference abstract. Consistency should be checked between the abstract evidence and full text evidence available in a review, as the risk of bias may be being impacted by the inclusion of evidence from the abstract only.

## 5 Conclusions

We have provided evidence of the potential changes that may occur and in which domains these changes may be most likely to occur. This could allow for understanding when including abstract‐only available evidence of the potential impact on future updates when full texts may be available. It is difficult to implement the RoB tool on conference abstracts. This is mainly owing to the reduced word count, which requires researchers to pick what information is vital to purvey to the readers. This led to several unclear ratings at the abstract level, with the expectation that the detail was not provided due to a lack of space. While the findings of this study should be interpreted within the context of small numbers, the evidence still suggests that, in some instances, such as allocation concealment and blinding, it is likely that the decision could change based on full‐text assessment. It would be worthwhile to consider what we would expect to be reported in a conference abstract and develop a tool for assessing such literature with greater accuracy. Current tools appear to be too cumbersome and lead to an unclear rating of the study. This can then, in turn, affect judgments on the certainty of evidence. In other review settings, these findings may be even more evident, for example, in prognostic modeling, the PROBAST is commonly used, which has numerous domains considering many complex signaling questions that are likely unavailable in a conference abstract. Further work is needed to solidify these results and replicate them in other areas. With future endeavors undertaken to create and validate tools for assessing conference abstracts that provide a clearer RoB judgment.

## Author Contributions

**Ryan P. W. Kenny:** conceptualisation, investigation, methodology, formal analysis, writing – original draft, writing review and editing. **Katie Twentyman:** investigation, methodology, writing – original draft, writing review and editing. **Dawn Craig:** methodology, writing review and editing. **Nick Meader:** investigation, methodology, formal analysis, writing review and editing. **Gill Norman:** conceptualisation, investigation, methodology, writing – original draft, writing review and editing.

## Funding

The authors have nothing to report.

## Conflicts of Interest

Nick Meader: I am a current employee of GlaxoSmithKline (GSK) and I also own shares in GSK. However, the current manuscript was prepared before I was in this employment and still with the NIHR Innovation Observatory. Gill Norman: Previous employment at the University of Manchester was partially funded by Cochrane Wounds (NIHR funding); I was an editor and methodologist with the group until March 2023. One of the systematic reviews included was authored by me (Norman et al. [2022] Negative pressure wound therapy for surgical wounds healing by primary closure). I had no input into the risk of bias assessments for any of the studies. The other authors declare no conflicts of interest.

## Supporting information

Supplementary material 1 model fit v3.

Supplementary material 2 Results ORs changes (2).

supplementary material 3.

## Data Availability

The data that support the findings of this study are available in the supplementary material of this article.
