# Supplementary material for: The Impact on Systematic Reviews of Risk of Bias Assessment Changes From Conference Abstracts to Full Text
Source: Cochrane Evid Synth Methods. 2026 Mar 27;4(3):e70078. doi: 10.1002/cesm.70078 (PMC13073319; doi:10.1002/cesm.70078)
Supplement: Supplementary file 1 — Supplementary material 1 model fit v3. [file CESM-4-e70078-s002.docx]

**Agreement between reviewers per domain of RoB1**

**Table 1.** The agreement between reviewers per domain for abstract and full text assessments.

| **Domain** | **Abstract/Full Text** | **Unweight Kappa (95% CI)** | **Weighted Kappa (95% CI)** |
| --- | --- | --- | --- |
| **1** | Abstract | 0.64 (0.19 to 1.00) | 0.67 (0.23 to 1.00) |
|  | Full Text | 0.53 (0.32 to 0.75) | 078 (0.62 to 0.95) |
| **2** | Abstract | 0.66 (0.04 to 1.00) | 0.67 (0.07 to 1.00) |
|  | Full Text | 0.51 (0.26 to 0.76) | 0.75 (0.53 to 0.97) |
| **3** | Abstract | 0.79 (0.57 to 1.00) | 0.82 (0.62 to 1.00) |
|  | Full Text | 0.81 (0.61 to 1.00) | 0.92 (0.83 to 1.00) |
| **4** | Abstract | 0.28 (-0.07 to 0.64) | 0.34 (0.02 to 0.66) |
|  | Full Text | 0.46 (0.22 to 0.71) | 0.58 (0.31 to 0.86) |
| **5** | Abstract | 0.12 (-0.09 to 0.31) | 0.01 (-0.28 to 0.30) |
|  | Full Text | 0.24 (-0.13 to 0.61) | 0.27 (-0.15 to 0.68) |
| **6** | Abstract | 0.37 (-0.18 to 0.91) | 0.41 (-0.08 to 0.91) |
|  | Full Text | 0.47 (0.16 to 0.78) | 0.30 (-0.15 to 0.74) |
| **7** | Abstract | 0.04 (-0.24 to 0.33) | -0.02 (-0.35 to 0.31) |
|  | Full Text | 0.32 (0.00 to 0.63) | 0.30 (-0.07 to 0.66) |

**Model code**

Full model example code:

Model <- brm(rating ~ 0 + type*domain.1 + (1|study), data = data, family = categorical(), chains = 4, iter = 5000, prior = prior1, thin = 1, warmup = 1500, save_pars = save_pars(all = TRUE)

Null model example code:

Model <- brm(rating ~ 0 + (1|study), data = data, family = catergorical(), chains = 4, iter = 5000, thin = 1, warmup = 1500, save_pars = save_pars(all = TRUE)

**Results when using “unclear” as the reference level**

**Table 1.** ORs of the multinomial analysis for each domain change from abstract to full text; reference level unclear.

| Domain | RoB rating | OR | 95% CrI |
| --- | --- | --- | --- |
| 1 | High | 1.39 | 0.46 to 4.24 |
|  | Low | 1.90 | 0.75 to 4.96 |
| 2 | High | 3.09 | 1.01 to 9.84 |
|  | Low | 2.93 | 1.13 to 7.87 |
| 3 | High | 5.09 | 1.67 to 16.20 |
|  | Low | 2.11 | 0.71 to 6.52 |
| 4 | High | 1.57 | 0.54 to 4.63 |
|  | Low | 1.22 | 0.48 to 3.16 |
| 5 | High | 0.63 | 0.18 to 2.18 |
|  | Low | 1.20 | 0.47 to 3.12 |
| 6 | High | 0.63 | 0.18 to 2.18 |
|  | Low | 1.20 | 0.47 to 3.12 |
| 7 | High | 0.26 | 0.18 to 0.37 |
|  | Low | 0.38 | 0.16 to 0.94 |

**WAIC (widely applicable information criterion) results**

The models for each domain outperform the null model, with a lower elpd and WAIC estimate. Additionally, it has a higher p_waic estimate. All these values suggest better predictive value and model fitting for the domain analyses. However, all models have p_waic estimates greater than 0.4, with the suggestion from the brm package to use the leave one out (loo) instead.

| **Model** | **Elpd_waic Estimate (SE)** | **P_waic Estimate (SE)** | **WAIC Estimate (SE)** | **P_waic estimates greater than 0.4 (%)** |
| --- | --- | --- | --- | --- |
| Domain 1 | -647.6 (15.4) | 53.7 (1.7) | 1295.1 (30.7) | 0 (0.0%) |
| Domain 2 | -641.9 (15.3) | 52.8 (1.7) | 1283.8 (30.6) | 0 (0.0%) |
| Domain 3 | -624.7 (15.8) | 54.8 (1.9) | 1249.5 (31.5) | 2 (0.3%) |
| Domain 4 | -645.8 (15.5) | 54.1 (1.7) | 1291.6 (31.0) | 0 (0.0%) |
| Domain 5 | -627.0 (16.3) | 55.4 (1.9) | 1254.0 (32.6) | 5 (0.7%) |
| Domain 6 | -621.6 (16.0) | 54.2 (1.9) | 1243 (32.0) | 6 (0.8%) |
| Domain 7 | -627.9 (16.0) | 55.1 (1.9) | 1255.9 (32.0) | 4 (0.6%) |
| Null model | -741.0 (8.6) | 56.5 (1.5) | 1482.0 (17.3) | 1 (0.1%) |

**LOO (leave one out) results**

The full model outperforms the null model, with lower elpd_loo and looic estimates, and a higher p_loo estimate.

| **Model** | **Elpd_loo Estimate (SE)** | **P_loo Estimate (SE)** | **Looic Estimate (SE)** | **Pareto k diagnostic good (-Inf, 0.7)** | **Pareto k diagnostic bad (0.7, 1)** | **Pareto k diagnostic very bad (1, Inf)** |
| --- | --- | --- | --- | --- | --- | --- |
| Domain 1 | NA | NA | NA | NA | NA | NA |
| Domain 2 | NA | NA | NA | NA | NA | NA |
| Domain 3 | -625.1 (15.8) | 55.1 (1.9) | 1250.1 (31.6) | All (100%) | NA | NA |
| Domain 4 | NA | NA | NA | NA | NA | NA |
| Domain 5 | -627.3 (16.3) | 55.7 (1.9) | 1254.6 (32.7) | All (100%) | NA | NA |
| Domain 6 | -621.9 (16.0) | 54.5 (1.9) | 1243.8 (32.1) | All (100%) | NA | NA |
| Domain 7 | -628.2 (16.0) | 55.4 (1.9) | 1256.5 (32.0) | All (100%) | NA | NA |
| Null model | -741.4 (8.7) | 56.9 (1.5) | 1482.7 (17.3) | All (100%) | NA | NA |

**Results when using “low” as the reference level**

**Table 2.** ORs of the multinomial analysis for each domain change from abstract to full text; reference level low.

| Domain | RoB rating | OR | 95% CrI |
| --- | --- | --- | --- |
| 1 | High | 0.99 | 0.33 to 3.17 |
|  | Unclear | 0.52 | 0.20 to 1.30 |
| 2 | High | 1.64 | 0.52 to 5.37 |
|  | Unclear | 0.27 | 0.10 to 0.70 |
| 3 | High | 2.70 | 0.96 to 5.54 |
|  | Unclear | 0.28 | 0.08 to 0.94 |
| 4 | High | 1.51 | 0.50 to 4.52 |
|  | Unclear | 0.76 | 0.29 to 1.90 |
| 5 | High | 0.45 | 0.14 to 1.45 |
|  | Unclear | 0.96 | 0.37 to 2.37 |
| 6 | High | 0.35 | 0.10 to 1.34 |
|  | Unclear | 1.24 | 0.49 to 3.13 |
| 7 | High | 0.35 | 0.12 to 0.97 |
|  | Unclear | 3.78 | 1.58 to 8.88 |

**WAIC (widely applicable information criterion) results**

The models for each domain outperform the null model, with a lower elpd and WAIC estimate. Additionally, it has a higher p_waic estimate. All these values suggest better predictive value and model fitting for the domain analyses. However, all models including a domain have p_waic estimates greater than 0.4, with the suggestion from the brm package to use the leave one out (loo) instead. The null model did not have any such values as was therefore not re-run using the loo function.

| **Model** | **Elpd_waic Estimate (SE)** | **P_waic Estimate (SE)** | **WAIC Estimate (SE)** | **P_waic estimates greater than 0.4 (%)** |
| --- | --- | --- | --- | --- |
| Domain 1 | -650.3 (15.4) | 55.2 (1.9) | 1300.6 (30.9) | 5 (0.7%) |
| Domain 2 | -642.6 (15.4) | 54.1 (1.9) | 1285.2 (30.8) | 5 (0.7%) |
| Domain 3 | -626.4 (16.0) | 56.0 (2.2) | 1252.7 (31.9) | 6 (0.8%) |
| Domain 4 | -648.7 (15.5) | 55.3 (1.9) | 1297.5 (31.1) | 5 (0.7%) |
| Domain 5 | -629.8 (16.3) | 56.2 (2.1) | 1259.7 (32.6) | 6 (0.8%) |
| Domain 6 | -623.3 (16.3) | 56.7 (2.2) | 1246.6 (32.6) | 9 (1.3%) |
| Domain 7 | -627.8 (16.1) | 56.8 (2.1) | 1255.5 (32.2) | 5 (0.7%) |
| Null model | -741.0 (8.7) | 56.8 (1.5) | 1482.1 (17.5) | NA |

**LOO (leave one out) results**

The full model outperforms the null model, with lower elpd_loo and looic estimates, and a higher p_loo estimate.

| **Model** | **Elpd_loo Estimate (SE)** | **P_loo Estimate (SE)** | **Looic Estimate (SE)** | **Pareto k diagnostic good (-Inf, 0.7)** | **Pareto k diagnostic bad (0.7, 1)** | **Pareto k diagnostic very bad (1, Inf)** |
| --- | --- | --- | --- | --- | --- | --- |
| Domain 1 | -650.6 (15.5) | 55.5 (1.9) | 1301.2 (30.9) | All (100%) | NA | NA |
| Domain 2 | -642.9 (15.4) | 54.4 (1.9) | 1285.8 (30.8) | All (100%) | NA | NA |
| Domain 3 | -626.7 (16.0) | 56.3 (2.2) | 12.53.4 (31.9) | All (100%) | NA | NA |
| Domain 4 | -649.0 (15.6) | 55.6 (1.9) | 1298.1 (31.1) | All (100%) | NA | NA |
| Domain 5 | -630.2 (16.3) | 56.6 (2.1) | 1260.3 (32.6) | All (100%) | NA | NA |
| Domain 6 | -623.7 (16.3) | 57.0 (2.2) | 1247.3 (32.6) | All (100%) | NA | NA |
| Domain 7 | -628.1 (16.1) | 57.1 (2.1) | 1256.2 (32.3) | All (100%) | NA | NA |
| Null model | NA | NA | NA | NA | NA | NA |

**Results when using “high” as the reference level**

**Table 3.** ORs of the multinomial analysis for each domain change from abstract to full text; reference level high.

| Domain | RoB rating | OR | 95% CrI |
| --- | --- | --- | --- |
| 1 | Low | 1.35 | 0.49 to 3.74 |
|  | Unclear | 0.59 | 0.21 to 1.58 |
| 2 | Low | 1.21 | 0.43 to 3.43 |
|  | Unclear | 0.27 | 0.09 to 0.77 |
| 3 | Low | 0.54 | 0.20 to 1.44 |
|  | Unclear | 0.17 | 0.05 to 0.54 |
| 4 | Low | 0.85 | 0.31 to 2.43 |
|  | Unclear | 0.61 | 0.22 to 1.63 |
| 5 | Low | 1.75 | 0.62 to 4.97 |
|  | Unclear | 1.61 | 0.52 o 4.92 |
| 6 | Low | 2.72 | 0.83 to 9.10 |
|  | Unclear | 2.67 | 0.86 to 8.14 |
| 7 | Low | 1.27 | 0.48 to 3.38 |
|  | Unclear | 5.49 | 1.92 to 15.31 |

**WAIC (widely applicable information criterion) results**

The models for each domain outperform the null model, with a lower elpd and WAIC estimate. Additionally, it has a higher p_waic estimate. All these values suggest better predictive value and model fitting for the domain analyses. However, all models including a domain have p_waic estimates greater than 0.4, with the suggestion from the brm package to use the leave one out (loo) instead. The null model did not have any such values as was therefore not re-run using the loo function.

| **Model** | **Elpd_waic Estimate (SE)** | **P_waic Estimate (SE)** | **WAIC Estimate (SE)** | **P_waic estimates greater than 0.4 (%)** |
| --- | --- | --- | --- | --- |
| Domain 1 | -651.1 (15.4) | 49.5 (1.4) | 1302.3 (30.8) | NA |
| Domain 2 | -645.5 (15.3) | 49.1 (1.4) | 1290.9 (30.6) | NA |
| Domain 3 | -630.2 (15.6) | 49.1 (1.5) | 1260.3 (31.3) | NA |
| Domain 4 | -649.4 (15.5) | 49.5 (1.5) | 1298.9 (30.9) | NA |
| Domain 5 | -630.7 (16.4) | 50.8 (1.7) | 1261.4 (32.7) | NA |
| Domain 6 | -625.7 (16.0) | 50.9 (1.6) | 1251.3 (32.1) | 1 (0.4%) |
| Domain 7 | -630.0 (16.2) | 51.7 (1.7) | 1260.0 (32.4) | NA |
| Null model | -752.8 (8.5) | 58.7 (1.0) | 1505.6 (17.1) | NA |

Due to some p_waic estimates being greater than 0.4, we performed leave one out (loo) analysis, as recommended by the brms package guidance.

**LOO (leave one out) results**

The full model outperforms the null model, with lower elpd_loo and looic estimates, and a higher p_loo estimate.

| **Model** | **Elpd_loo Estimate (SE)** | **P_loo Estimate (SE)** | **Looic Estimate (SE)** | **Pareto k diagnostic good (-Inf, 0.7)** | **Pareto k diagnostic bad (0.7, 1)** | **Pareto k diagnostic very bad (1, Inf)** |
| --- | --- | --- | --- | --- | --- | --- |
| Domain 1 | NA | NA | NA | NA | NA | NA |
| Domain 2 | NA | NA | NA | NA | NA | NA |
| Domain 3 | NA | NA | NA | NA | NA | NA |
| Domain 4 | NA | NA | NA | NA | NA | NA |
| Domain 5 | NA | NA | NA | NA | NA | NA |
| Domain 6 | -625.9 (16.0) | 51.1 (1.6) | 1251.8 (32.1) | All (100%) | NA | NA |
| Domain 7 | NA | NA | NA | NA | NA | NA |
| Null model | NA | NA | NA | NA | NA | NA |

**Included studies**

| **Area of Study** | **Review** | **Abstract Reference** | **Full Text Reference** |
| --- | --- | --- | --- |
| Alzheimer's Disease | Mason et al 2013 Drug therapy for obstructive sleep apnoea in adults. DOI: 10.1002/14651858.CD003002.pub3 | Carley et al 2003 Serotonin antagonist improves obstructive sleep apnoea. Sleep Medicine 4 Suppl 1:6 | Carley et al 2007 efficacy of mirtazapine in obstructive sleep apnea syndrome. Sleep Vol. 30, issue 1:35-41. |
|  | McShane et al 2019 Memantine for dementia. DOI: 10.1002/14651858.CD003154.pub6 | Reisberg & Ferris 2000 Results of a placebo-controlled 6-month trial with memantine in moderate to severe Alzheimer’s disease (AD). Journal of the European College of Neuropsychopharmacology 10(suppl  3):S363-4 | Reisberg et al 2003 Memantine in moderate to  severe Alzheimer´s Disease. New England Journal of Medicine 348:1333-41. |
| Anxiety | Moore et al 2018 Communication skills training for healthcare professionals working with people who have cancer. DOI: 10.1002/14651858.CD003751.pub4 | Fujimori 2011 Effect of communication skills training program for oncologists based on the patient preferences for communicating bad news: A randomized control trial. Psycho-Oncology Conference: 13th World Congress of Psycho-Oncology of the International Psycho-Oncology Society, IPOS Antalya Turkey. Conference Start: 20111016 Conference End: 20111020 | Fujimori et al 2014 Effect of communication skills training program for oncologists based on patient preferences for communication when receiving bad news: a randomized controlled trial.  Journal of Clinical Oncology 32(20):2166-72. DOI: 10.1200/JCO.2013.51.2756 |
|  |  | Gibon et al 2011 Is it possible to improve radiotherapy team communication skills? A randomized study assessing the efficacy of a training program in the context of an encounter with a simulated anxious patient called Mrs Leblanc. Psycho-Oncology. 2011; Vol. 13th World Congress of the International  Psycho-Oncology Society 16/01/2011 -20/01/2011, Antalya, Turkey:102. DOI: 10.1002/pon.2077 | Merckaert et al 2015 Transfer of communication skills to the workplace: impact of a 38-hour communication skills training program designed for radiotherapy teams. Journal of Clinical  Oncology 33(8):901-9. DOI: 10.1200/JCO.2014.57.3287 |
|  | Pollock et al 2019 Interventions for visual field defects in people with stroke. DOI: 10.1002/14651858.CD008388.pub3 | Plow et al 2010 Combining non-invasive cortical  stimulation with vision rehabilitation to improve visual function in post-stroke hemianopia. In: American Academy of Neurology  (AAN) Meeting; April 2010; Toronto. | Plow et al 2012 Comparison of visual field training for hemianopia with active versus sham transcranial direct cortical stimulation. Neurorehabilitation and Neural Repair 26(6):616-26 |
|  | Howcroft et al 2016 Action plans with brief patient education for exacerbations in chronic obstructive pulmonary disease. DOI: 10.1002/14651858.CD005074.pub4 | McGlone et al 2004 The effect of a written  action plan in COPD. Respirology 9(2 Suppl):A46. | Wood-Baker et al 2006 Written action  plans in chronic obstructive pulmonary disease increase appropriate treatment for acute exacerbations. Respirology 11(5):619-26 |
|  | Mangesi et al 2015 Fetal movement counting for assessment of fetal wellbeing. DOI: 10.1002/14651858.CD004909.pub3 | Gomez et al 2003 A comparison of fetal movement chart by high risk women. American Journal of Obstetrics and Gynecology 189(6):S179 | Gomez et al 2007 Compliance with a fetal movement chart by high-risk obstetric patients in a Peruvian hospital. American Journal of Perinatology 24(2):89-93. |
| Cancer | Akl et al 2017 Parenteral anticoagulation in ambulatory patients with cancer. DOI: 10.1002/14651858.CD006652.pub5 | Pelzer et al 2009 A prospective, randomized trial of  simultaneous pancreatic cancer treatment with enoxaparin and chemotherapy - first results of the CONKO 004 trial. In: Onkologie - DGHO meeting. Vol. 580. October 2009 | Pelzer et al 2015 Efficacy of prophylactic low–molecular weight heparin for ambulatory patients with advanced pancreatic cancer: outcomes from the CONKO-004 trial. Journal of Clinical Oncology 33(18):2028-34. |
|  | Robertson et al 2021 Effect of testing for cancer on cancer‐ or venous thromboembolism (VTE)‐related mortality and morbidity in people with unprovoked VTE. DOI: 10.1002/14651858.CD010837.pub5 | Piccoli et al 2012 The value of thoraco-abdominal CT scanning for the detection of occult cancer in patients with unprovoked venous thromboembolism. A randomized study. Thrombosis Research 129 Suppl 1:S155-94 | Prandoni et al 2016 Extensive computed tomography versus limited screening for detection of occult cancer in unprovoked venous thromboembolism: a multicenter, controlled, randomized clinical trial. Seminars in Thrombosis and Hemostasis 42(8):884-90 |
|  | O'Carrigan et al 2017 Bisphosphonates and other bone agents for breast cancer. DOI: 10.1002/14651858.CD003474.pub4 | Llombart et al 2009 Effect of zoledronic acid on aromatase inhibitor associated bone loss in postmenopausal women with early breast cancer receiving adjuvant letrozole: E-ZO-FAST 36-month follow-up. ASCO Breast Cancer Symposium; 2009; Chicago. Chicago: American Society of Clinical Oncology | Llombart et al 2012 Immediate administration of zoledronic acid reduces aromatase inhibitor-associated bone loss in postmenopausal women with early breast cancer: 12- month analysis of the E-ZO-FAST trial. Clinical Breast Cancer 12(1):40-8 |
|  | Gaitskell et al 2023 Angiogenesis inhibitors for the treatment of epithelial ovarian cancer. DOI: 10.1002/14651858.CD007930.pub3 | Burger et al 2010 Gynaecologic Oncology Group study. Safety and subgroup eNicacy analyses in GOG218, a phase III trial of bevacizumab (BEV) in the primary treatment of advanced epithelial ovarian cancer (EOC), primary peritoneal cancer (PPC) or fallopian tube cancer (FTC). Annals of Oncology 2010;21(Suppl):Abstr 978PD viii307 | Burger et al 2011 Incorporation of bevacizumab in the primary treatment of ovarian cancer. New England Journal of Medicine 365:2473-83 |
|  |  | Karlan et al 2010 Randomized, double-blind, placebo-controlled phase II study of AMG 386 combined with weekly paclitaxel in patients (pts) with recurrent ovarian carcinoma. Journal of  Clinical Oncology 28(Suppl 15):5000 | Karlan et al 2012 Randomized, double-blind, placebo-controlled phase II study of AMG 386 combined with weekly paclitaxel in patients with recurrent ovarian cancer. Journal of Clinical  Oncology 30(4):362-71. |
|  |  | Ledermann et al 2009 A randomized phase II placebo-controlled trial using maintenance therapy to evaluate the vascular targeting agent BIBF 1120 following treatment of relapsed ovarian cancer (OC). Journal of Clinical Oncology 2(Suppl 15):5501. | Ledermann et al 2011 Randomized phase II placebo-controlled trial of maintenance therapy using the oral triple angiokinase inhibitor BIBF 1120 aKer chemotherapy for relapsed ovarian cancer. Journal of Clinical Oncology 29(28):3798-804. |
|  |  | Perren 2010 et al ICON7: a phase III randomised Gynaecologic Cancer Intergroup trial of concurrent bevacizumab and chemotherapy followed by maintenance bevacizumab, versus chemotherapy alone in women with newly diagnosed epithelial ovarian (EOC), primary peritoneal (PPC) or fallopian tube cancer (FTC). Annals of Oncology 21(Suppl 8):LBA4. | Oza et al 2015 Standard chemotherapy with or without bevacizumab for women with newly diagnosed ovarian cancer (ICON7): overall survival results of a phase 3 randomised trial. Lancet Oncology 16(8):928-36. |
|  |  | Vergote et al 2009 Aflibercept (VEGF Trap) in advanced ovarian cancer patients with recurrent symptomatic malignant ascites: results of a randomized, double-blind, placebo-controlled study. International Journal of Gynecological Cancer 19:Suppl 2 | Gotlieb et al 2012 Intravenous aflibercept for treatment of  recurrent symptomatic malignant ascites in patients with advanced ovarian cancer: a phase 2, randomised, double-blind, placebo-controlled study. Lancet Oncology 2012;13:154-62. |
|  | Akl et al 2018 Anticoagulation for people with cancer and central venous catheters. DOI: 10.1002/14651858.CD006468.pub6 | Young et al 2005 WARP - a multicenter prospective randomised controlled trial (RCT) of thrombosis prophylaxis with warfarin in cancer patients with central venous catheters (CVCs). Journal of Clinical Oncology 23(16S):8004 | Young et al 2009 Warfarin thromboprophylaxis in cancer patients with central venous catheters (WARP): an open-label randomised trial. Lancet 373(9663):567-74. |
|  | Egger et al 2004 Platinum‐containing regimens for metastatic breast cancer. DOI: 10.1002/14651858.CD003374.pub4 | Icli et al 2002 Paclitaxel (T) vs cisplatin + VP-16 (EP) in metastatic breast cancer patients treated with anthracyclines: A phase III randomized study, Turkish Oncology Group. Annals of Oncology Vol. 13:47. | Icli et al 2005 Cisplatin plus oral etoposide (EoP) combination is more eGective than paclitaxel in patients with advanced breast cancer pretreated with anthracyclines: a randomised phase III trial of Turkish Oncology Group. British Journal of Cancer 92:639-44. |
|  |  | Fountzilas et al 2002 Paclitaxel and epirubicin versus paclitaxel and carboplatin as first-line chemotherapy in patients with advanced breast cancer: A phase III study conducted by the Hellenic Cooperative Oncology Group. Annals of Oncology 13(Suppl 5):46-69 | Fountzilas et al 2004 Paclitaxel and epirubicin versus paclitaxel and carboplatin as first-line chemotherapy in patients with advanced breast cancer: a phase III study conducted by the Hellenic Cooperative Oncology Group. Annals of Oncology 15(10):1517-26 |
|  | O'Rouke et al 2010 Concurrent chemoradiotherapy in non‐small cell lung cancer. DOI: 10.1002/14651858.CD002140.pub3 | Huber et al 2003 Induction chemotherapy and following simultaneous radio/chemotherapy versus induction chemotherapy and radiotherapy alone in inoperable NSCLC (stage IIIA/IIIB). Proceedings of the American Society of Clinical Oncology 22:622. | Huber et al 2006 Simultaneous chemoradiotherapy compared with radiotherapy alone aQer induction chemotherapy in inoperable stage IIIA or IIIB non-small-cell lung cancer: study CTRT99/97 by the Bronchial Carcinoma Therapy Group. Journal of clinical oncology : o)icial journal of the American Society of Clinical Oncology 24(27):4397-404. |
|  |  | Manegold et al 2003 A phase II randomized study comparing docetaxel/cisplatin induction therapy followed by thoracic radiotherapy with or without weekly docetaxel in unresectable stage IIIA-IIIB non-small cell lung cancer. European Journal of  Cancer 1 Suppl(5):248-9. | Scagliotti et al 2006 Docetaxel-based induction therapy prior to radiotherapy with or without docetaxel for non-small-cell lung cancer. British Journal of Cancer 94:1375-82. [DOI: 10.1038/sj.bjc.6603115] |
|  | Tonia et al 2012 Erythropoietin or darbepoetin for patients with cancer. DOI: 10.1002/14651858.CD003407.pub5 | Machtay et al 2004 Definitive radiotherpay +/- erythropoietin for squamous cell carcinoma of the head and neck: preliminary report of RTOG 99-03. International Journal of Radiation Oncology, Biology, Physics. Vol. 60 (Suppl 1):S132. | Machtay et al 2007 Radiotherapy with or without  erythropoietin for anemic patients with head and neck cancer: a randomized trial of the Radiation Therapy Oncology Group (RTOG 99-03). International Journal of Radiation Oncology,  Biology, Physics 69(4):1008-17 |
|  |  | Razzouk et al 2004 Influence of hemoglobin response to epoetin alfa on quality-of-life in anemic children with cancer receiving myelosuppressive chemotherapy. Blood Vol. 104, issue 11:abstract 221. | Razzouk et al 2006 Double-blind, placebo-controlled study of quality of life, hematologic end points, and safety of weekly epoetin alfa in children with cancer receiving myelosuppressive chemotherapy. Journal of Clinical Oncology 24(22):3583-9 |
|  |  | Savonije et al 2004 Early intervention with epoetin-alfa during platinum-based chemotherapy. Journal of Clinical Oncology. Vol. 22, issue 14S:#8111. | Savonije et al 2005 Effects of early intervention with epoetin alfa on transfusion requirement, hemoglobin level and survival during platinum-based chemotherapy: Results of a multicenter randomised controlled trial. European Journal of Cancer 41(11):1560-9. |
|  | Wilson et al 2019 Taxanes for adjuvant treatment of early breast cancer. DOI: 10.1002/14651858.CD004421.pub3 | Crown et al 2006 Docetaxel given concurrently with or sequentially to anthracycline-based adjuvant therapy for patients with node-positive breast cancer, in comparison with non-taxane combination chemotherapy: first results of the BIG 2-98 trial at 5 years median follow-up. Journal of Clinical Oncology 24(Suppl 18):LBA519. | Francis et al 2008 Adjuvant chemotherapy with sequential or concurrent anthracycline and docetaxel: Breast International Group 02-98 randomized trial. Journal of the National Cancer Institute 100(2):121-33 |
|  |  | Goldstein et al 2005 Phase III AT vs. AC in the adjuvant treatment of node-positive and high-risk nodenegative breast cancer. Journal of Clinical Oncology 23(16 Suppl 7):Abstract 512. | Goldstein et al 2008 Concurrent doxorubicin plus docetaxel is not more eCective than concurrent doxorubicin plus cyclophosphamide in operable breast cancer with 0 to 3 positive axillary nodes: North American Breast Cancer Intergroup Trial E 2197. Journal of Clinical Oncology 26(25):4092-9 |
|  |  | Bianco et al 2006 Sequential epirubicin-docetaxel-CMF as adjuvant therapy of early breast cancer: results of the Taxit216 multicenter phase III trial. Journal of Clinical Oncology 24:LBA520 | Forestieri 2008 Docetaxel in adjuvant therapy of breast cancer: results of the TAXIT 216 multicenter phase III trial. Docetaxel in Adjuvant Therapy of Breast Cancer: Results of the TAXIT 216 Multicenter Phase III Trial. Naples, Italy: University of Naples Federico II |
| Cardiovascular disease | Redfern et al 2024 | Padney et al 2014 Text message reminders to address medication non-adherence in post-MI patients: a one year intervention study. Canadian Journal of Cardiology 30(10):S179. [DOI: 10.1016/j.cjca.2014.07.280] | Padney 2017 The impact of text messaging on medication adherence and exercise among postmyocardial infarction patients: randomized controlled pilot trial. JMIR mHealth and uHealth 5(8):e110. [DOI: 10.2196/ mhealth.7144] |
|  | Schmidt et al 2020 | Stroes et al 2015 Efficacy and safety of 150 mg and 300 mg every 3 weeks in patients with poorly controlled hypercholesterolemia: the ODYSSEY CHOICE I and CHOICE II studies. Journal of the American College of Cardiology 5:99.1. | Roth et al 2016 A phase III randomized trial evaluating alirocumab 300 mg every 4 weeks as monotherapy or add-on to statin: ODYSSEY CHOICE I. Atherosclerosis 2016;1:254-62. |
|  |  | Stroes et al 2015 Efficacy and safety of 150 mg and 300 mg every 3 weeks in patients with poorly controlled hypercholesterolemia: the ODYSSEY CHOICE I and CHOICE II studies. Journal of the American College of Cardiology 5:99.1. | Stroes et al 2016 Efficacy and safety of alirocumab 150 mg every 4 weeks in patients with hypercholesterolemia not on statin therapy: the ODYSSEY CHOICE II Study. Journal |
|  |  | Ginsberg et al 2014 ODYSSEY HIGH FH: efficacy and safety of alirocumab in patients with severe heterozygous familial hypercholesterolemia. www.resource.heartonline.cn/20150520/2_NaN0BW2.pdf | Ginsberg et al 2016 Efficacy and safety of alirocumab in patients with heterozygous familial hypercholesterolemia and LDLC of 160 mg/dl or higher. Cardiovascular Drugs Therapy 30:473-83 |
| Depression | Cavalheri et al 2019 Exercise training undertaken by people within 12 months of lung resection for non‐small cell lung cancer. DOI: 10.1002/14651858.CD009955.pub3 | Brocki et al 2010 Rehabilitation after lung cancer operation- a randomised controlled study. European Respiratory Society 2010 Annual Congress | Brocki et al 2014 Short and long-term effects of supervised versus unsupervised exercise training on health-related quality of life and functional outcomes following lung cancer surgery - a randomized controlled trial. DOI: 10.1016/j.lungcan.2013.10.015 |
|  | Denis & Dowswell 2013 Psychosocial and psychological interventions for preventing postpartum depression. DOI: 10.1002/14651858.CD001134.pub3 | Gamble & Greedy 2003 Reducing postpartum emotional distress: a randomised controlled trial. [abstract]. Perinatal Society of Australia and New Zealand. 7th Annual Congress; 2003 March 9-12; Tasmania, Australia. A29. | Gamble et al 2005 Effectiveness of a counseling intervention after a traumatic childbirth: a randomized controlled trial. Birth 32(1):11-9. |
|  | Goldbeck et al 2014 Psychological interventions for individuals with cystic fibrosis and their families. DOI: 10.1002/14651858.CD003148.pub3 | Powers et al 1999 Behavioral treatment to improve nutrition in toddlers with cystic fibrosis [abstract]. Pediatric Pulmonology Suppl 19:329. | Powers et al 2003 A randomized pilot study of behavioral treatment to increase calorie intake in toddlers with cystic fibrosis. Child Health Care 32:297-311. |
|  | Linde et al 2008 St John's wort for major depression. DOI: 10.1002/14651858.CD000448.pub3 | Fava et al 2002 A double-blind randomised trial of St John's wort, fluoxetine and placebo in major depressive disorder. New research program and abstract of the 155th Annual meeting of the American Psychiatric Association. 69 | Fava et al 2005 A double-blind, randomized trial of St John's wort, fluoxetine, and placebo in major depressive disorder. Journal of Clinical Psychopharmacology 25:441-7. |
| Musculoskeletal | Agnew et al 2023 Interventions for weight reduction in obesity to improve survival in women with endometrial cancer. DOI: 10.1002/14651858.CD012513.pub3 | Allison et al 2016 Lifestyle beyond cancer: obesity and weight loss in endometrial cancer survivors: a randomized, multisite trial. In: TREC (Transdisciplinary Research on Energetics and Cancer) Scientific Grantee's Meeting, National Cancer Institute. March 2016. | Haggerty et al 2017 A randomized, controlled, multicenter study of technology-based weight loss interventions among endometrial cancer survivors. Obesity 25:S102-S8. [DOI: 10.1002/oby.22021] |
| Parkinson's Disease | Tomlinson et al 2013 Physiotherapy versus placebo or no intervention in Parkinson's disease. DOI: 10.1002/14651858.CD002817.pub4 | Canning et al 2008 Homebased treadmill walking for individuals with Parkinson's disease: a pilot randomized controlled trial. Movement Disorders 23(Suppl 1):637. | Canning et al 2012 Home-based treadmill training for individuals with Parkinson's disease: a randomized controlled pilot trial.. Clinical Rehabilitation 26(9):817-826 |
|  |  | Earhart et al 2010 Short-term eFects of a community-based tango program on motor and non-motor symptoms, activities of daily living, and motor complications in PD. Movement Disorders Vol. 25, issue Suppl 3:S697-8. | Duncan et al 2012 Randomized controlled trial of community-based dancing to modify disease progression in Parkinson disease. Neurorehabilitation & Neural Repair 26(2):132-43 |
|  |  | Goodwin et al 2009 Preventing falls in Parkinson's disease: the GETuP trial. Parkinsonism & Related Disorders 15(Suppl 2):S83. | Goodwin et al 2011 An exercise intervention to prevent falls in people with Parkinson's disease: a pragmatic randomised controlled trial . Journal of Neurology, Neurosurgery & Psychiatry 82(11):1232-8 |
| Surgery and Wounds | Norman et al 2022 Negative pressure wound therapy for surgical wounds healing by primary closure. DOI: 10.1002/14651858.CD009261.pub7 | Hussamy et al 2018 A randomized trial of closed incision negative pressure therapy in morbidly obese women undergoing cesarean delivery. American Journal of Obstetrics and Gynecology 218(1):S35. | Hussamy et al 2019 Closed incision negative pressure therapy in morbidly obese women undergoing cesarean delivery: a randomized controlled trial. Obstetrics and Gynecology 134(4):781-9. |
|  |  | Ruhstaller et al 2017 PROphylactic wound VACuum therapy after cesarean section to prevent wound complications in the obese population: a randomised controlled trial (the PROVAC study). American Journal of Obstetrics and Gynecology 216(1 Suppl 1):S34. | Ruhstaller et al 2017 Prophylactic wound vacuum therapy after cesarean section to prevent wound complications in the obese population: a randomized controlled trial (the ProVac study). American Journal of Perinatology 34(11):1125. |
|  |  | Martin et al 2019 Negative-pressure therapy for hepatectomy and pancreatectomy: a randomized trial for surgical site infection prevention. Hepato Pancreato Biliary Journal 21(Suppl 1):S26-7. | O'Neil et al 2020 Negative-pressure wound therapy does not reduce superficial SSI in pancreatectomy and hepatectomy procedures. Journal of Surgical Oncology 122(3):480-6. |
|  | Carson et al 2021 Transfusion thresholds for guiding red blood cell transfusion. DOI: 10.1002/14651858.CD002042.pub5 | Colomo et al 2008 Transfusion strategies in patients with cirrhosis and acute gastrointestinal bleeding. Hepatology 48(4 Suppl):413A | Villanueva et al 2013 Transfusion strategies for acute upper gastrointestinal bleeding. New England Journal of Medicine 368(1):11-21. |
|  | Guenga et al 2011 Mechanical bowel preparation for elective colorectal surgery DOI: 10.1002/14651858.CD001544.pub4 | Zmora et al 2002 LeR-side anastomosis without mechanical bowel preparation: a randomised, prospective trial. Diseases of Colon and Rectum Vol. 45, issue 4:A7-A8. | Zmora et al 2003 Colon and rectal surgery without mechanical bowel preparation. A randomised prospective trial. Annals of Surgery 237:363-7. |
|  | Hahn et al 2019 Target of rapamycin inhibitors (TOR‐I; sirolimus and everolimus) for primary immunosuppression in kidney transplant recipients DOI: https://doi.org/10.1002/14651858.CD004290.pub3 | Durrbach et al 2004 Use of sirolimus as initial therapy after renal transplantation: preliminary results of a randomized pilot study in patient receiving marginal kidneys. Transplantation 78(2 Suppl):228 | Durrbach et al 2008 Prospective comparison of the use of sirolimus and cyclosporine in recipients of a kidney from an expanded criteria donor. Transplantation 85(3):486-90 |
|  |  | Gallon et al 2003 A prospective randomized single center study of prednisone free immunosuppression that compares two maintenance combinations: tacrolimus/mycophenolate mofetil versus tacrolimus/sirolimus. Journal of the American Society of Nephrology 14(Nov):664A. | Gallon et al 2006 Long-term renal allograft function on a tacrolimus-based, pred-free maintenance immunosuppression comparing sirolimus vs MMF. American Journal of Transplantation 6(7):1617-23 |
|  |  | Glotz et al 2005 6 months preliminary results of a randomized trial comparing sirolimus (SRL) versus tacrolimus (FK) in 141 transplant patients receiving a cadaveric renal graft. American Journal of Transplantation 5(Suppl 11):460. | Glotz et al 2010 Thymoglobulin induction and sirolimus versus tacrolimus in kidney transplant recipients receiving mycophenolate mofetil and steroids. Transplantation 89(12):1511-7 |
|  |  | Kumar et al 2003 Comparison of tacrolimus (FK506) and sirolimus (SRL) combination with FK506 and mycophenolate mofetil (MMF) in kidney transplant recipients with steroid avoidance. American Journal of Transplantation 3(Suppl 5):350 | Anil Kumar et al 2005 Comparison of steroid avoidance in tacrolimus/mycophenolate mofetil and tacrolimus/sirolimus combination in kidney transplantation monitored by surveillance biopsy. Transplantation 80(6):807-14. |
|  |  | Lebranchu et al 2004 Preliminary results of a randomized trial comparing sirolimus (SRL) versus cyclosporine (CsA) in 150 transplant patients receiving a cadaveric renal graD. Transplantation 78(2 Suppl):463 | Lebranchu et al 2012 Five-year results of a randomized trial comparing de novo sirolimus and cyclosporine in renal transplantation: the SPIESSER study. American Journal of Transplantation 12(7):1801-10 |
|  |  | Paczek et al 2003 An open-label, concentration-controlled, randomised 6-month study of standard-dose tacrolimus + sirolimus +steroids compared to reduced-dose tacrolimus + sirolimus + steroids in renal allograft recipients. American Journal of Transplantation 3(Suppl 5):464 | Bechstein et al 2013 A comparative, randomized trial of concentration-controlled sirolimus combined with reduced-dose tacrolimus or standard-dose tacrolimus in renal allograft recipients. Transplantation Proceedings 45(6):2133-40. |
|  |  | Pescovitz et al 2004 Pharmacokinetics, safety and efficacy of mycophenolate mofetil in combination with sirolimus vs cyclosporine in renal transplant patients. American Journal of Transplantation 4(Suppl 8):251. | Pescovitz et al 2007 Pharmacokinetics, safety, and efficacy of mycophenolate mofetil in combination with sirolimus or ciclosporin in renal transplant patients. British Journal of Clinical Pharmacology 64(6):758-71. |
|  | Ker et al 2015 Antifibrinolytic drugs for acute traumatic injury DOI: https://doi.org/10.1002/14651858.CD004896.pub4 | Yutthakasemsunt et al 2010 Tranexamic Acid for preventing progressive intracranial hemorrhage in adults with traumatic brain injury; a preliminary report. National Neurotrauma Symposium. 14-17 July 2010 | Yutthakasemsunt et al 2013 Tranexamic acid for patients with traumatic brain injury: a randomized, double-blinded, placebo-controlled trial. BMC Emergency Medicine 13(20):doi:10.1186/1471-227X-13-20 |
|  | Lin et al 2023 Hyperbaric oxygen therapy for late radiation tissue injury DOI: https://doi.org/10.1002/14651858.CD005005.pub5 | Clarke et al 2004 Treatment of radiation necrosis with hyperbaric oxygen: a randomized double-blind placebo controlled trial. Undersea and Hyperbaric Medicine 31:Suppl | Clarke et al 2008 Hyperbaric oxygen treatment of chronic refractory radiation proctitis: a randomised and controlled double-blind crossover trial with long-term follow up. International Journal of Radiation Oncology, Biology, Physics 72:134-43. |

**Conditional plots**

*Conditional plots when using unclear as the reference level*


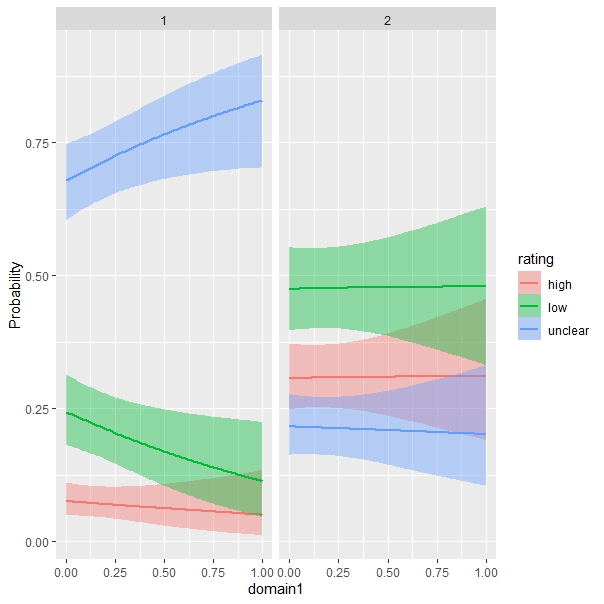

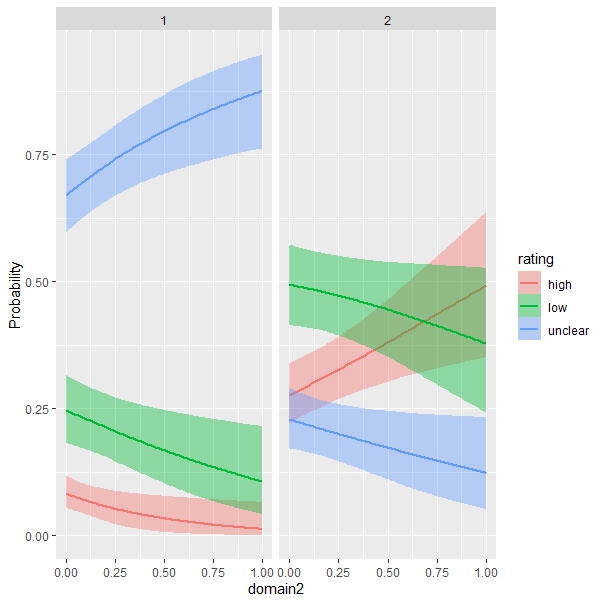

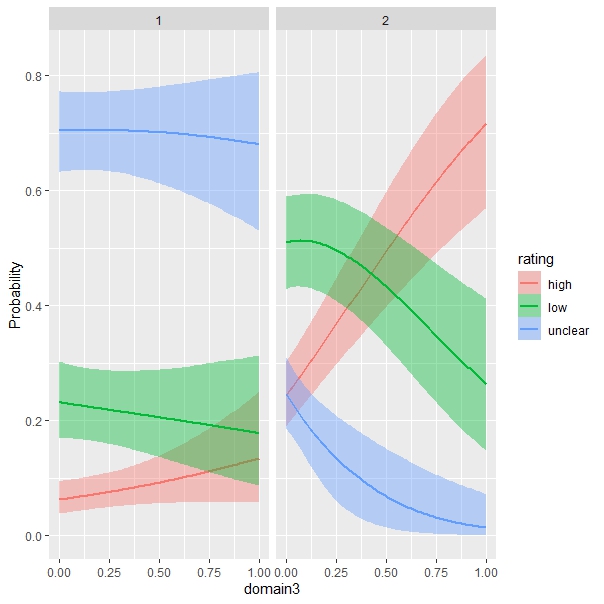

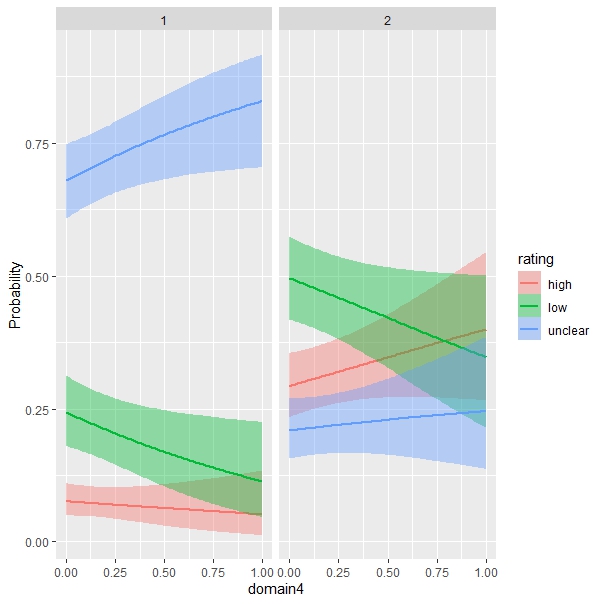

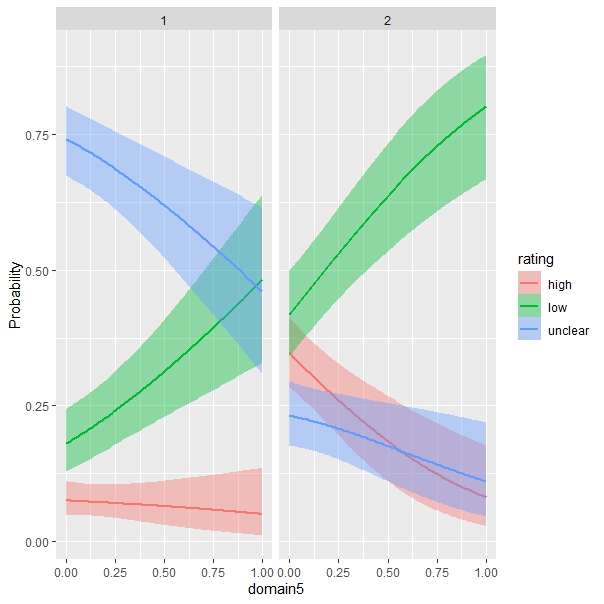

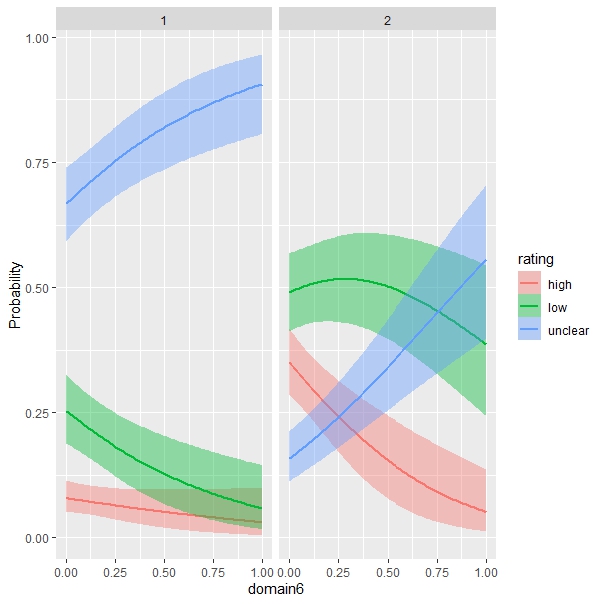

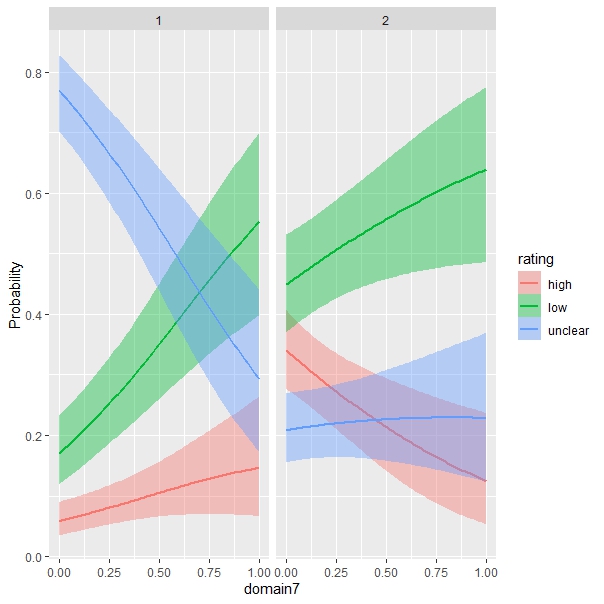


*Conditional plots when using low as the reference level*


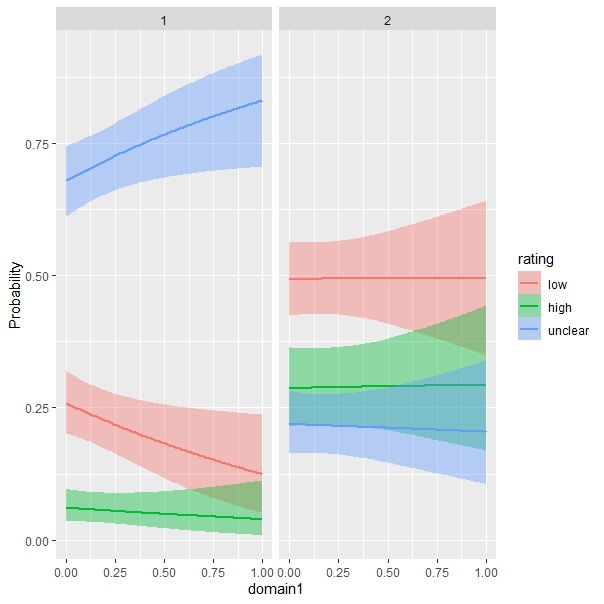

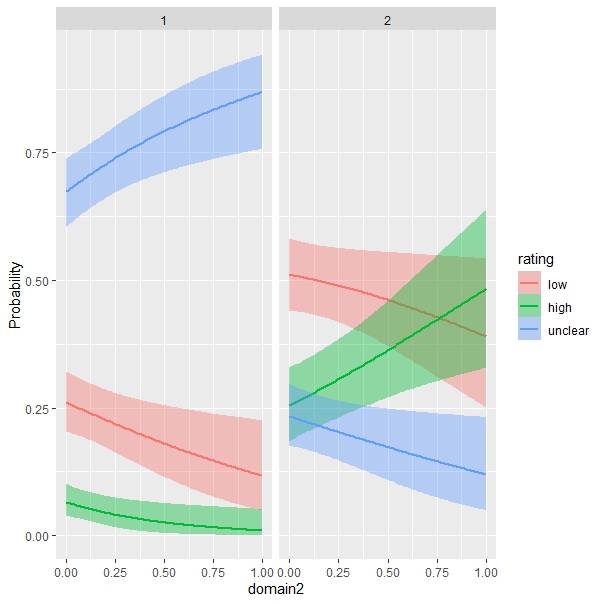

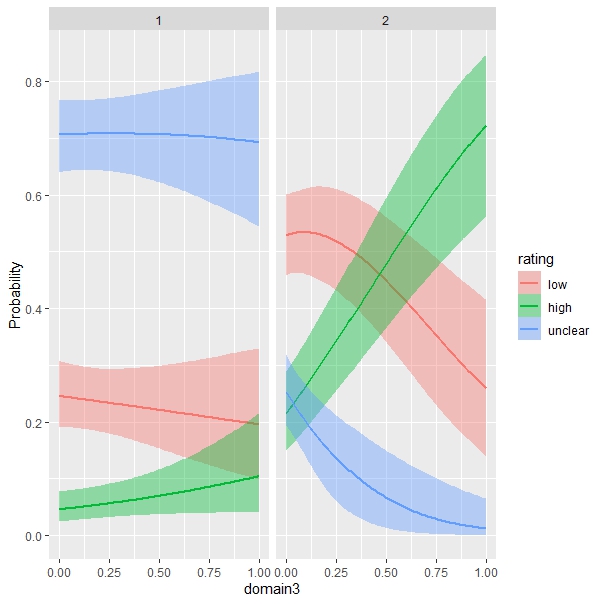

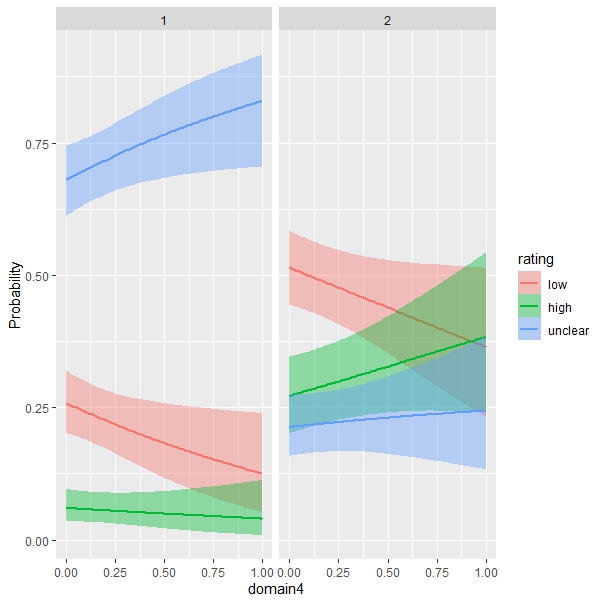

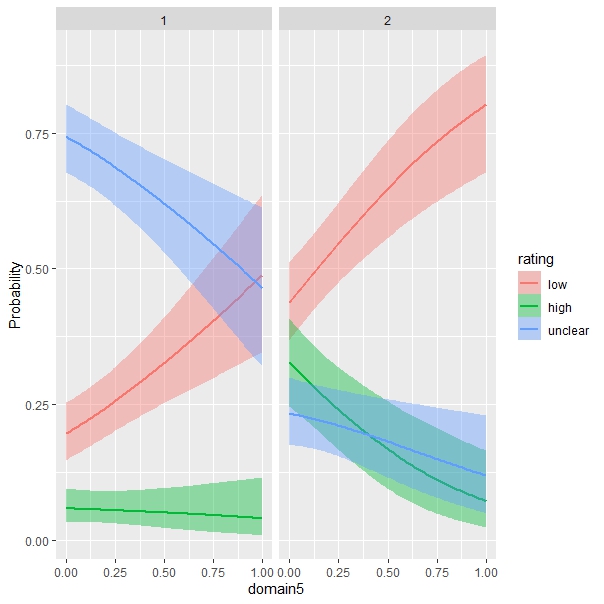

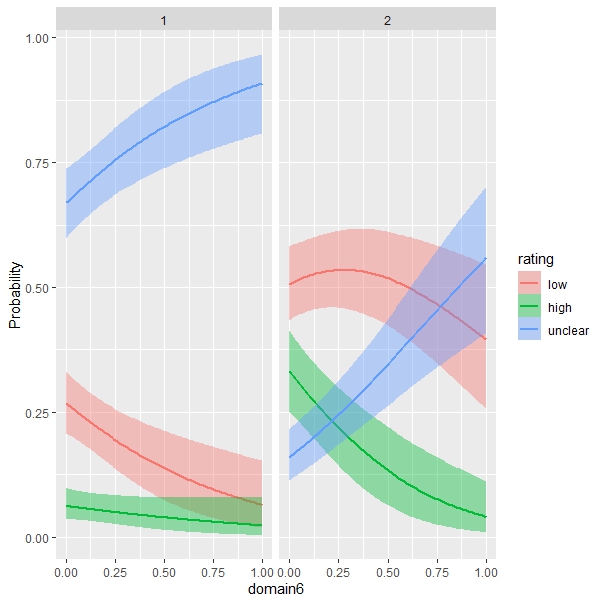

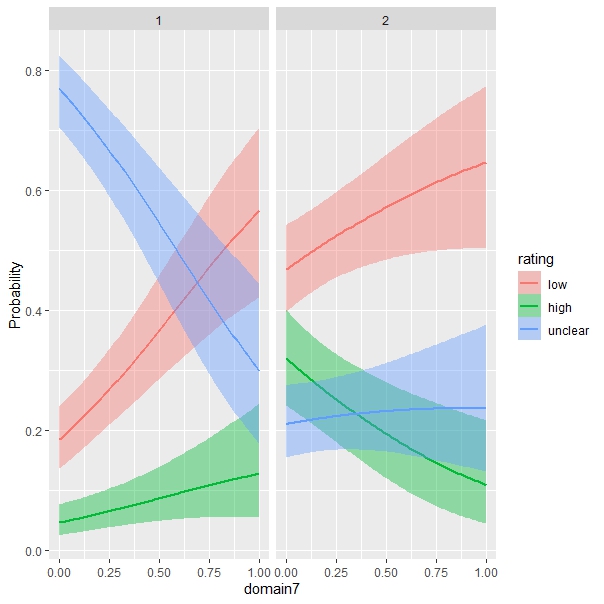


*Conditional plots when using high as the reference level*


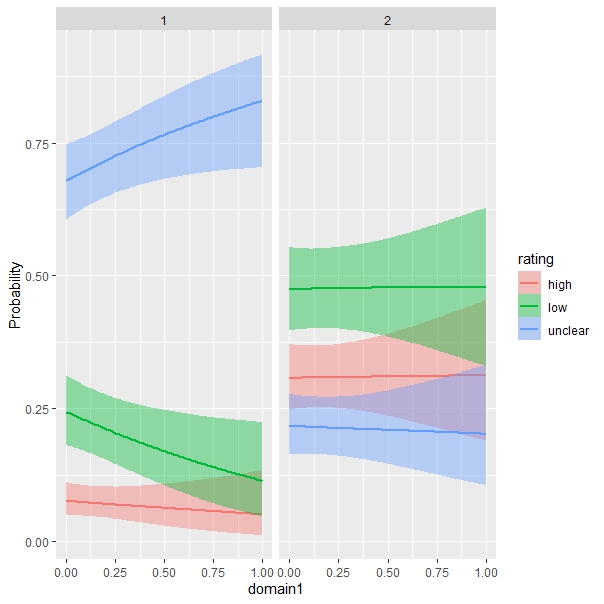

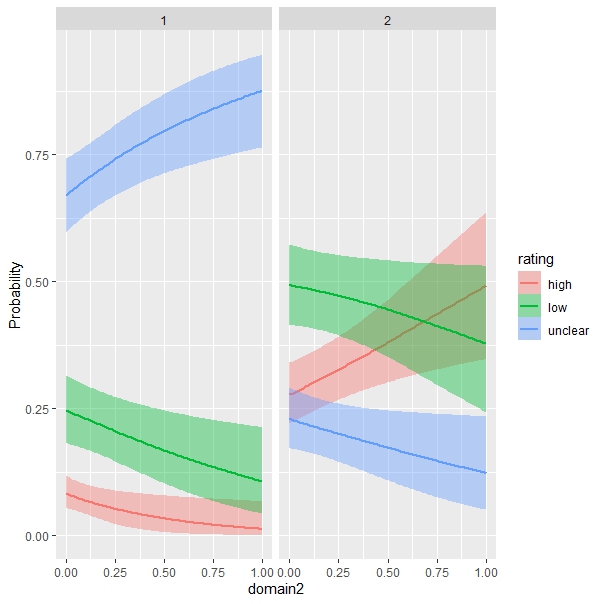

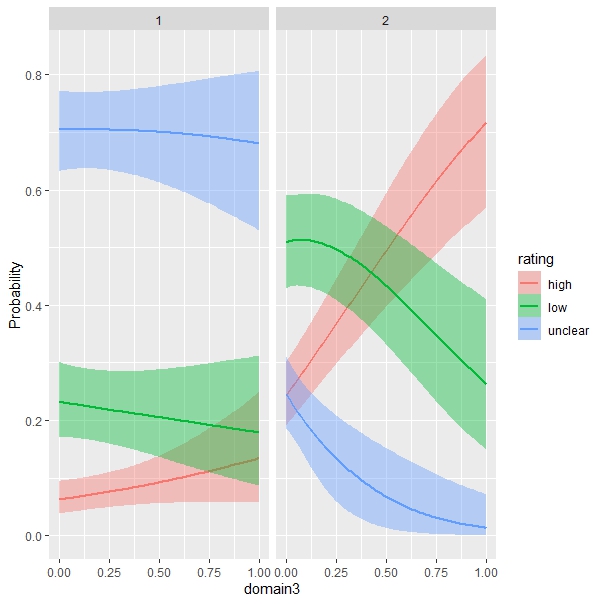

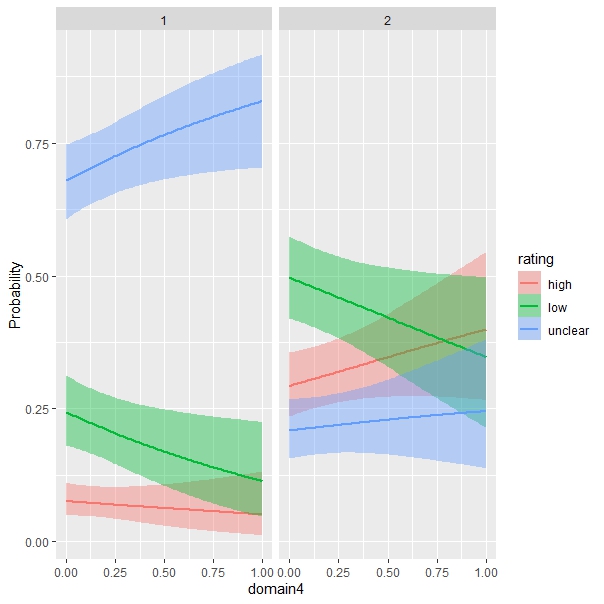

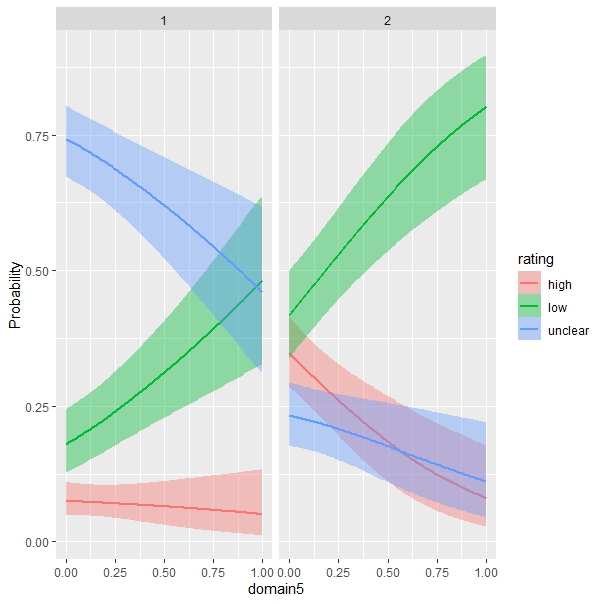

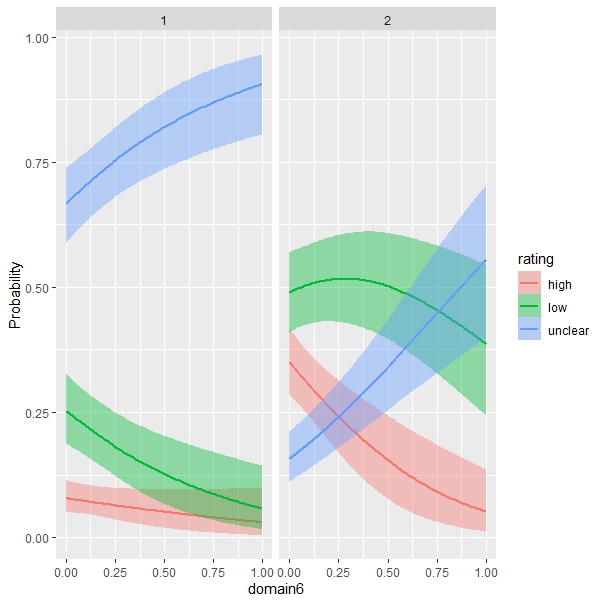

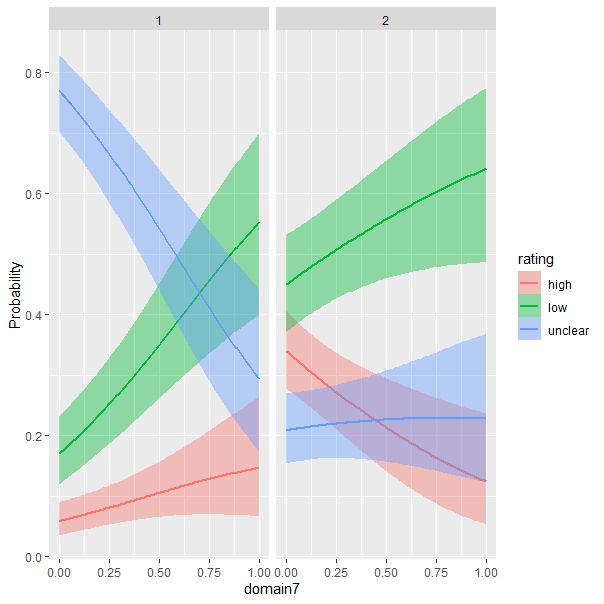


**Posterior Plots**

*Unclear reference*


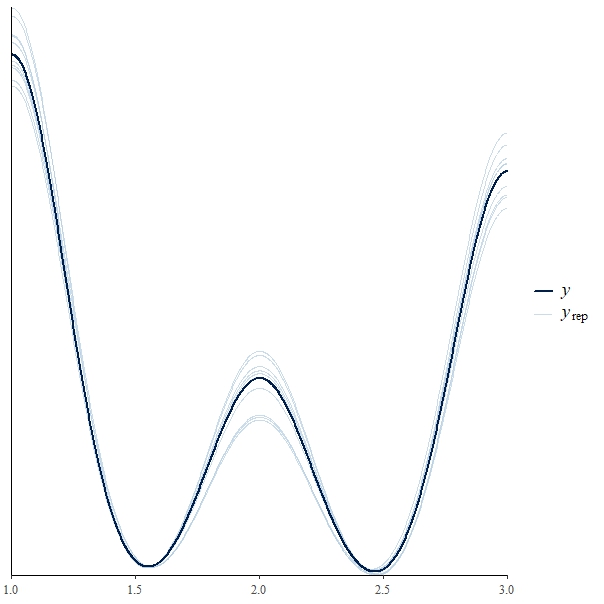


Domain 1


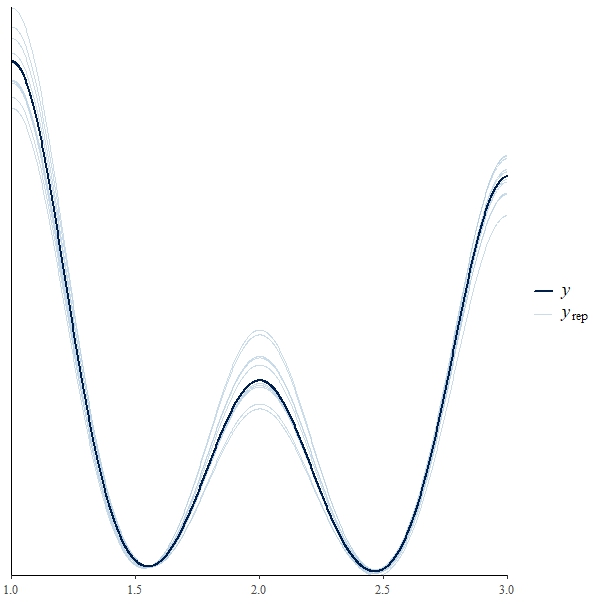


Domain 2


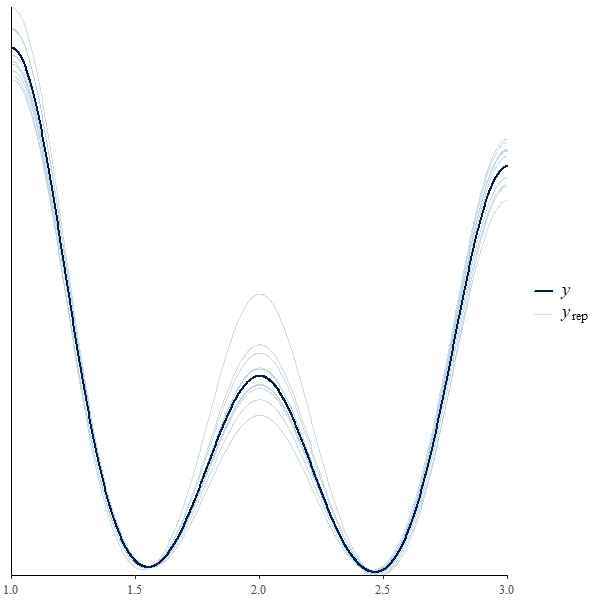


Domain 3


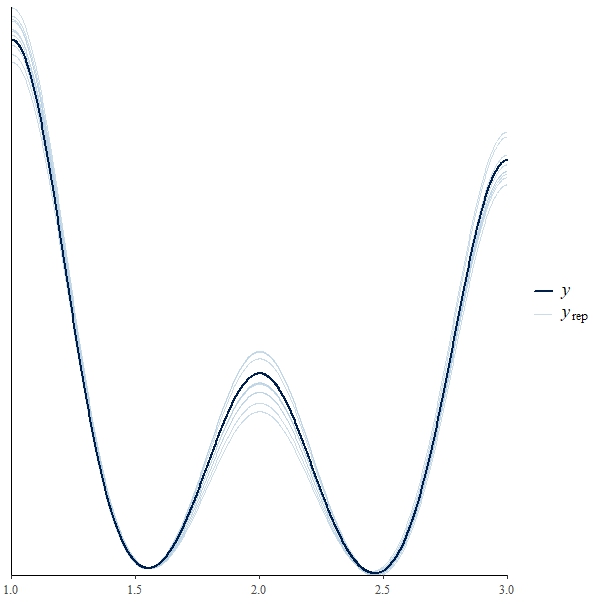


Domain 4


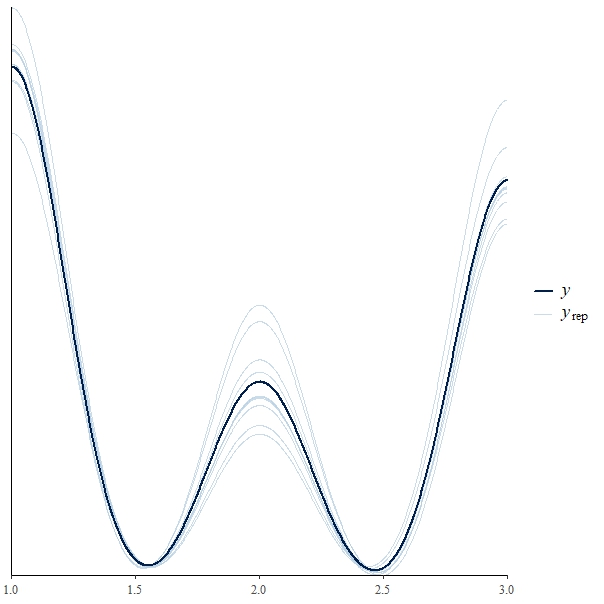


Domain 5


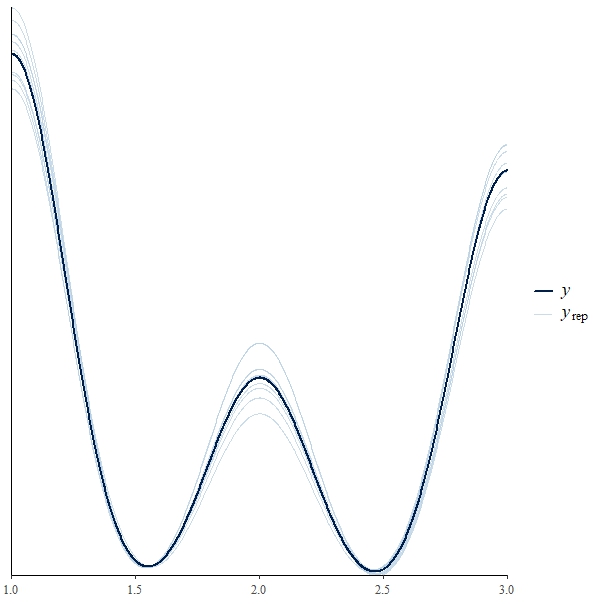


Domain 6


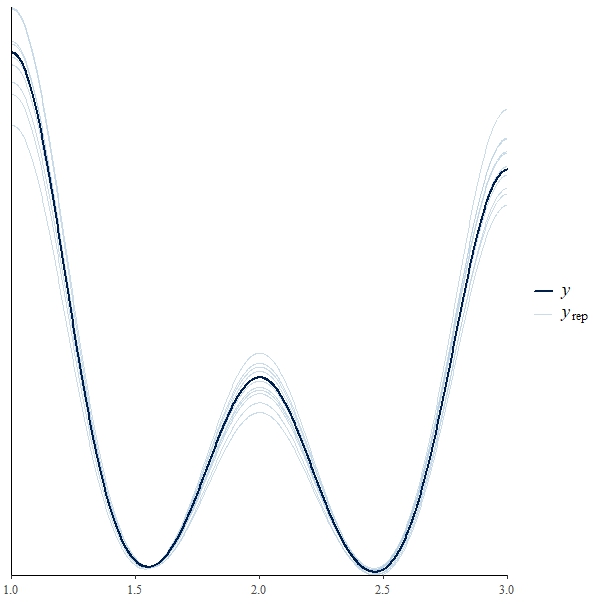


Domain 7

*Low reference*


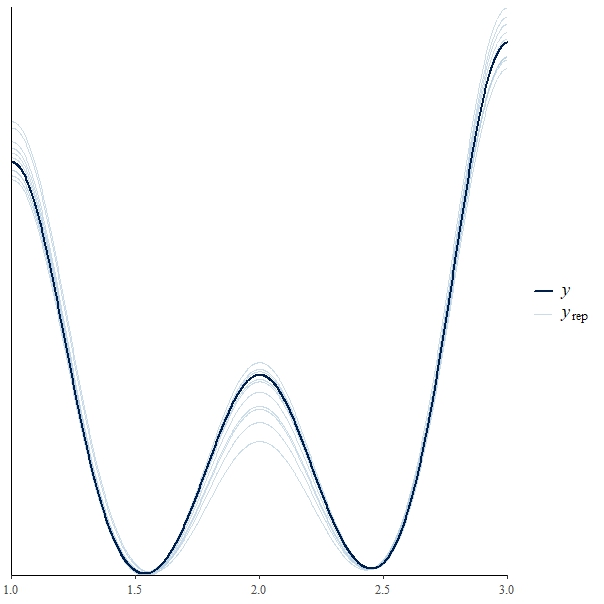


Domain 1


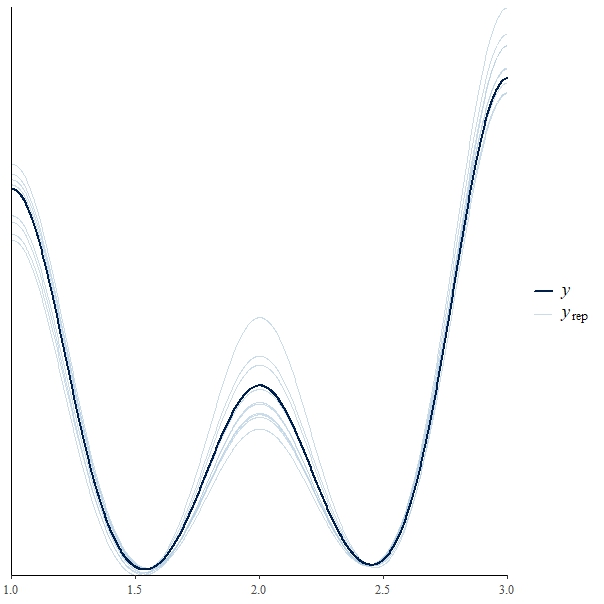


Domain 2


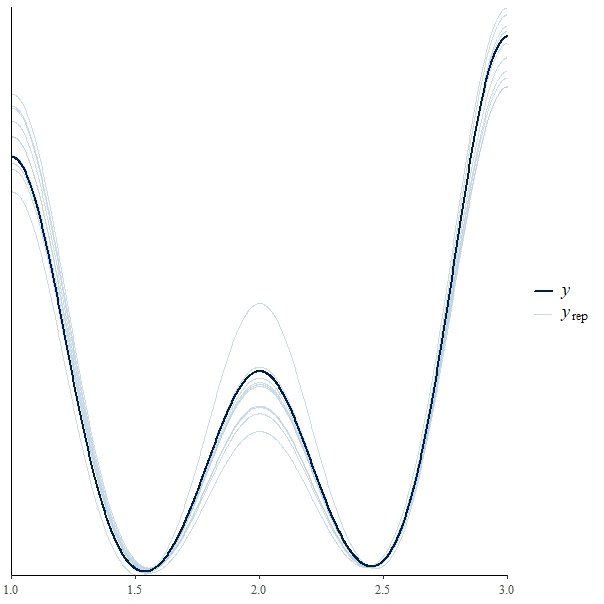


Domain 3


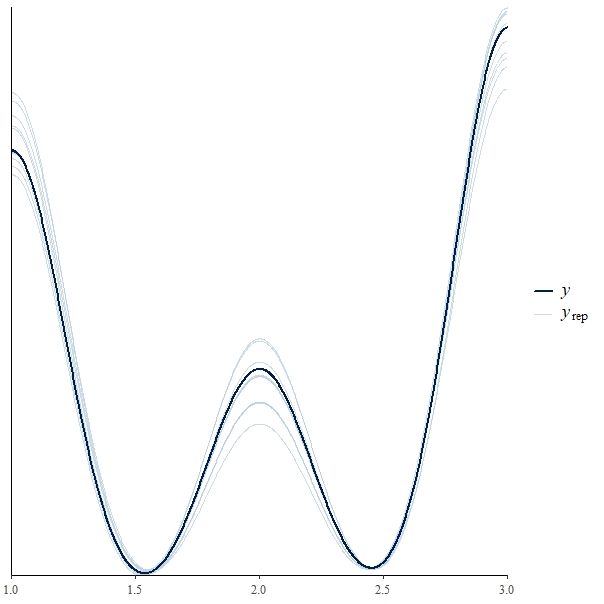


Domain 4


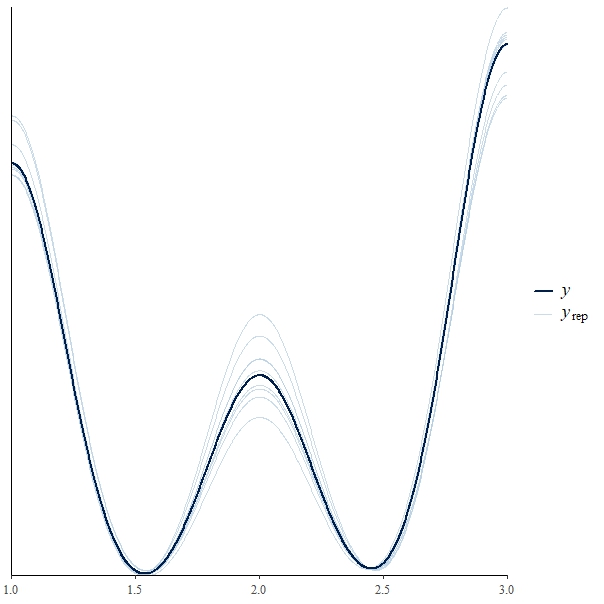


Domain 5


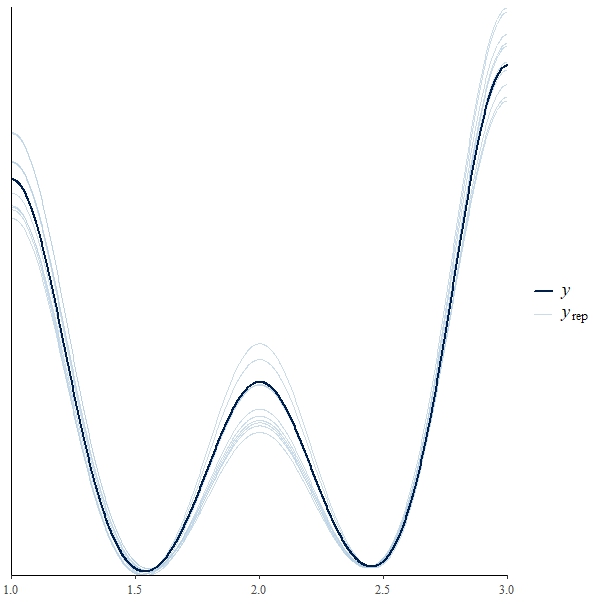


Domain 6


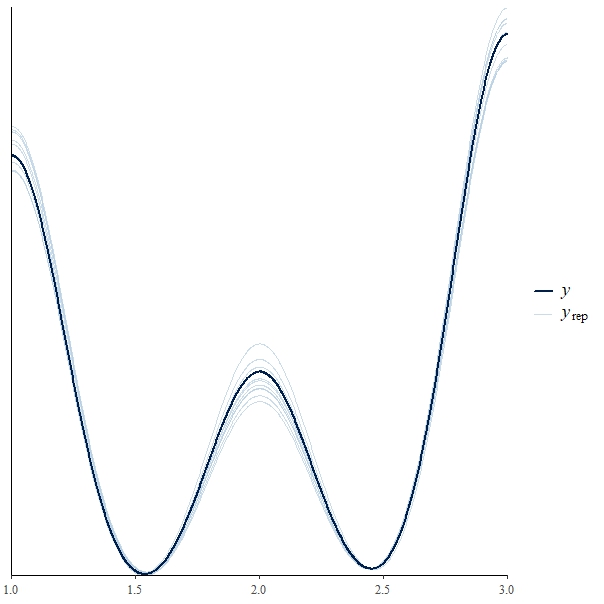


Domain 7

*High reference*


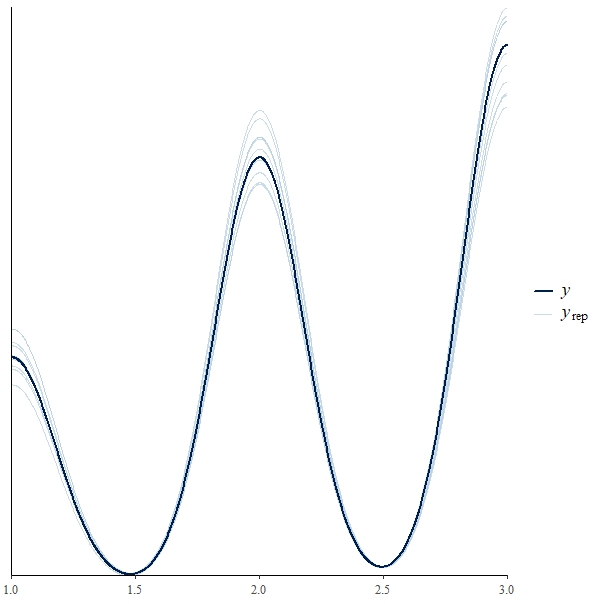


Domain 1


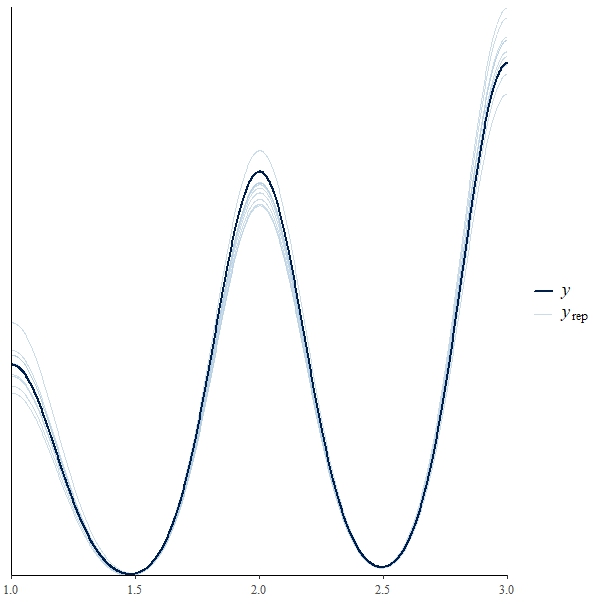


Domain 2


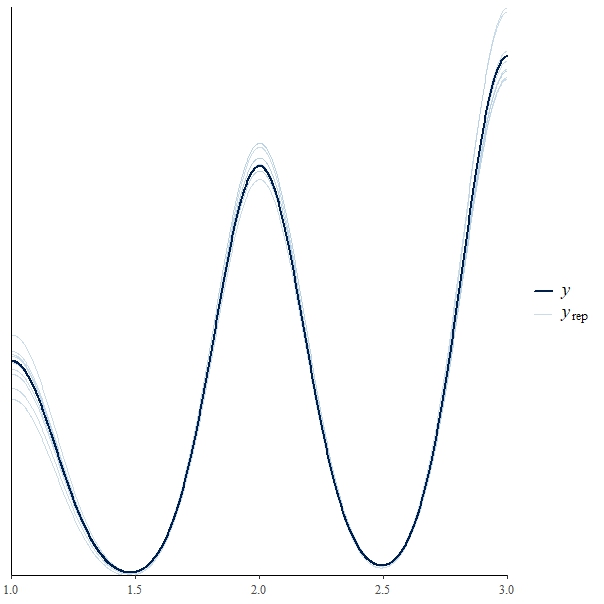


Domain 3


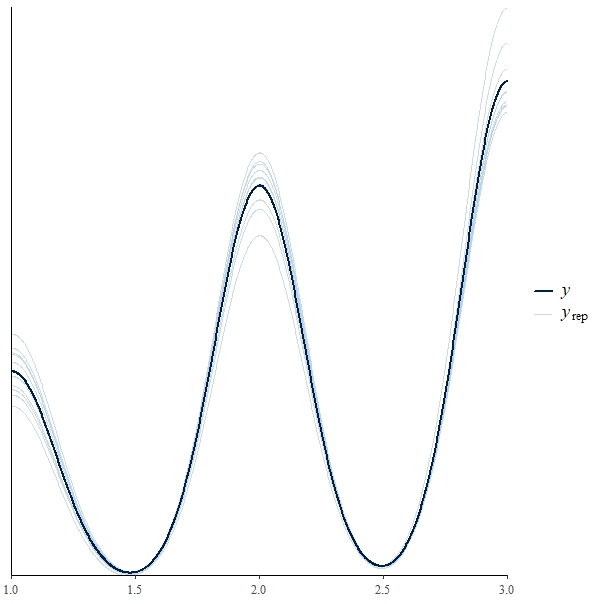


Domain 4


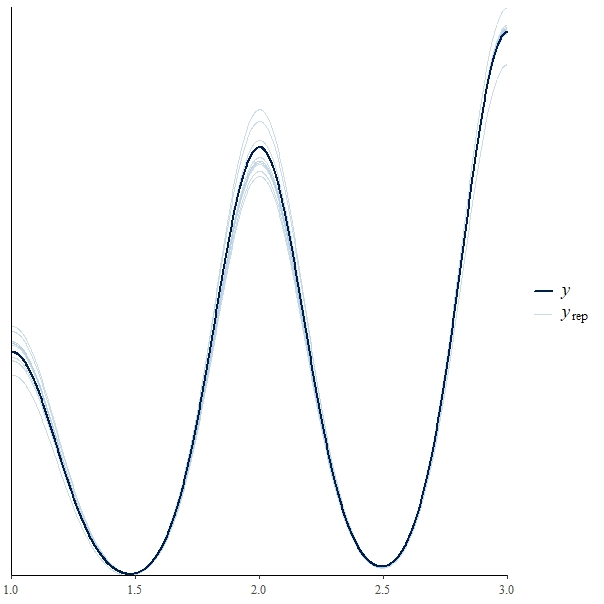


Domain 5


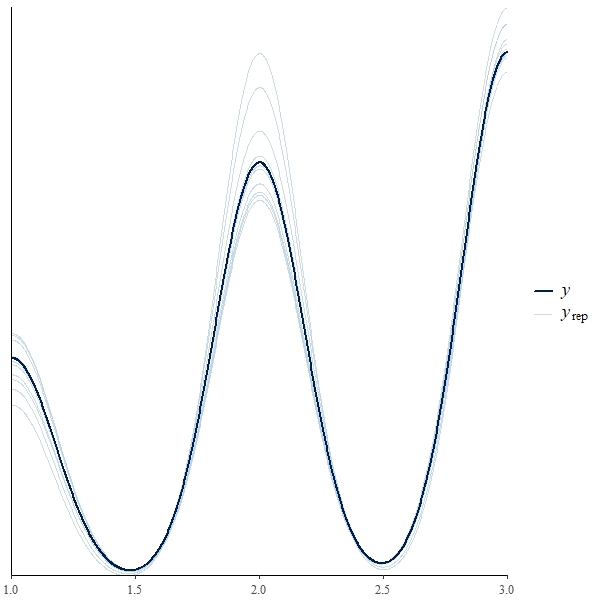


Domain 6


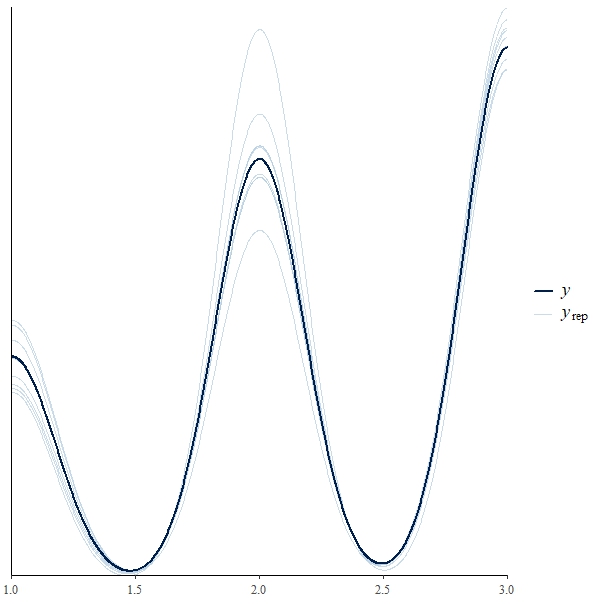


Domain 7

**Trace plots**

*Unclear reference*


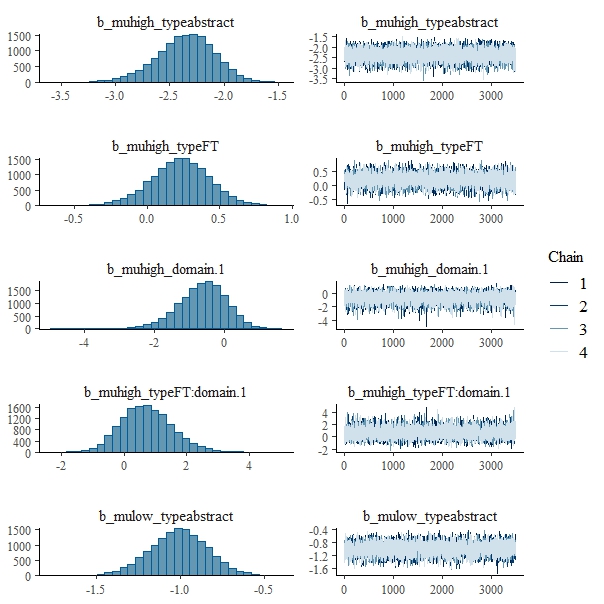

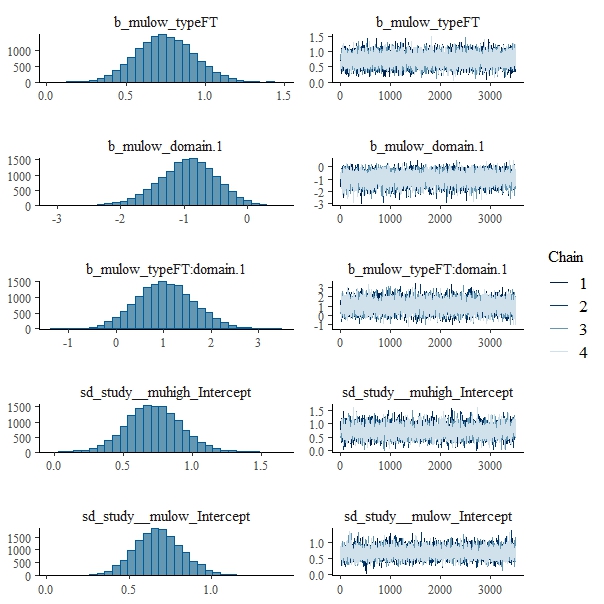


Domain 1


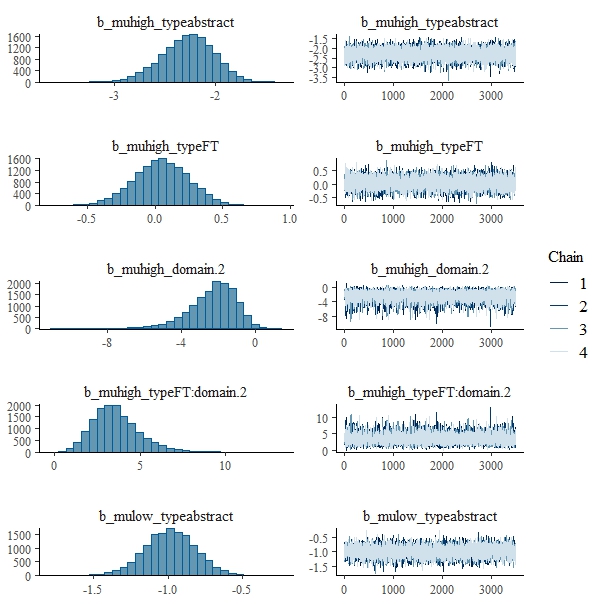

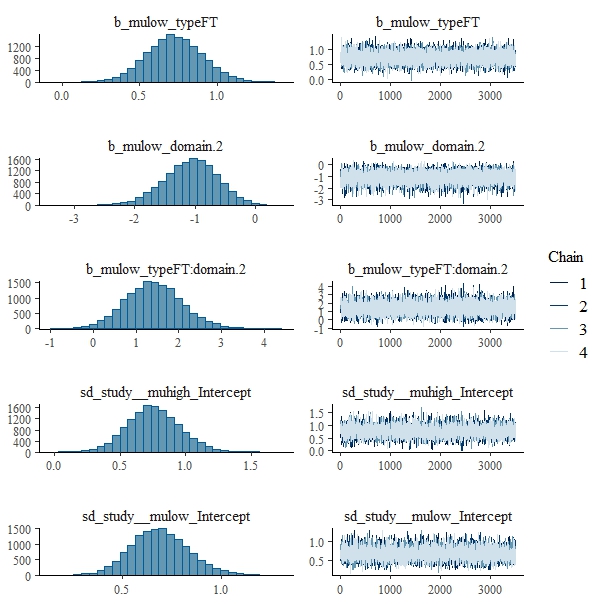


Domain 2


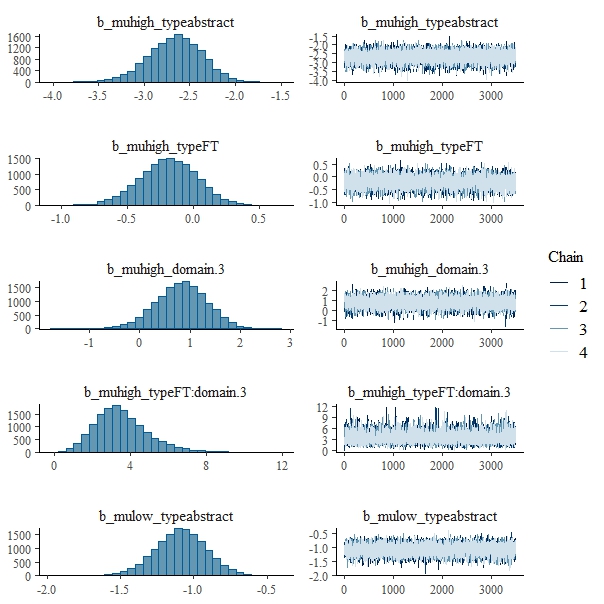

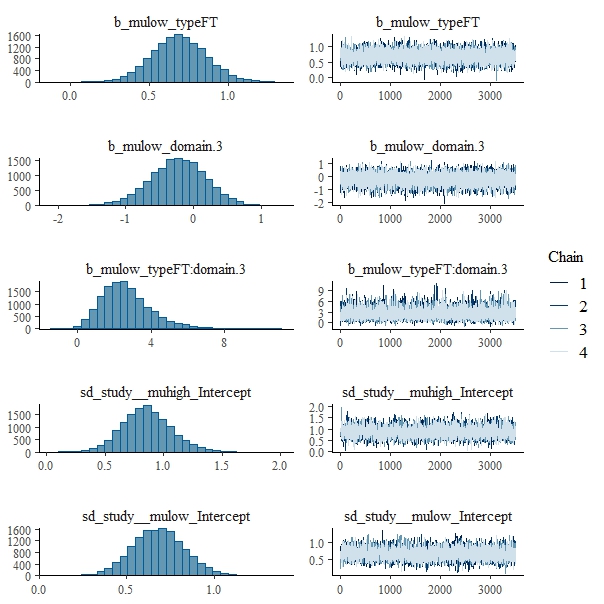


Domain 3


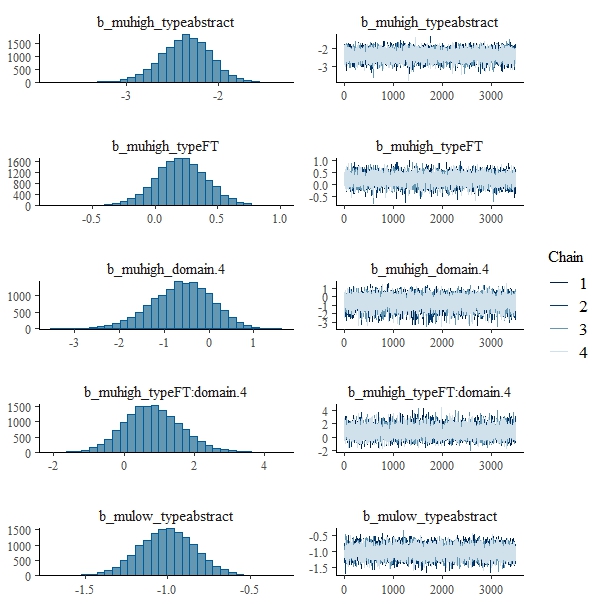

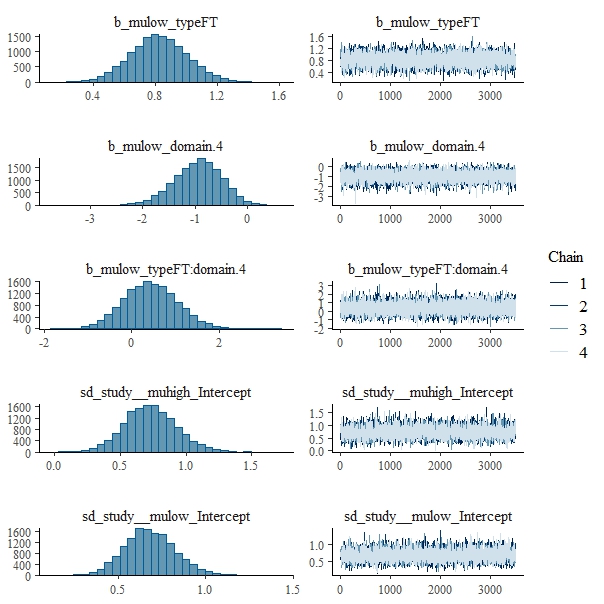


Domain 4


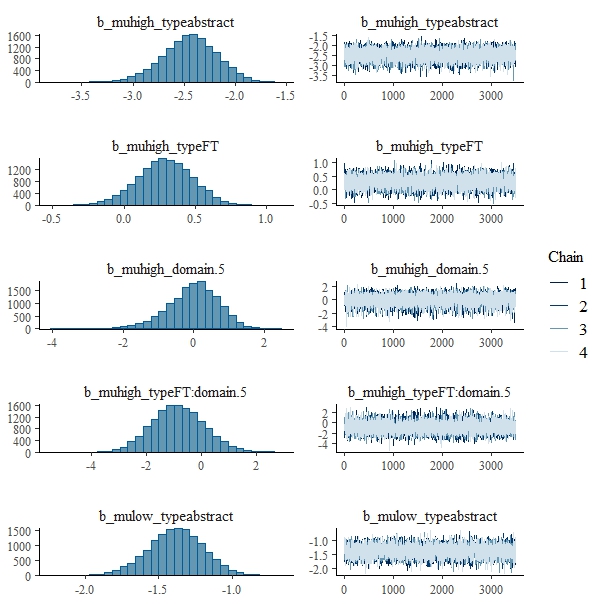

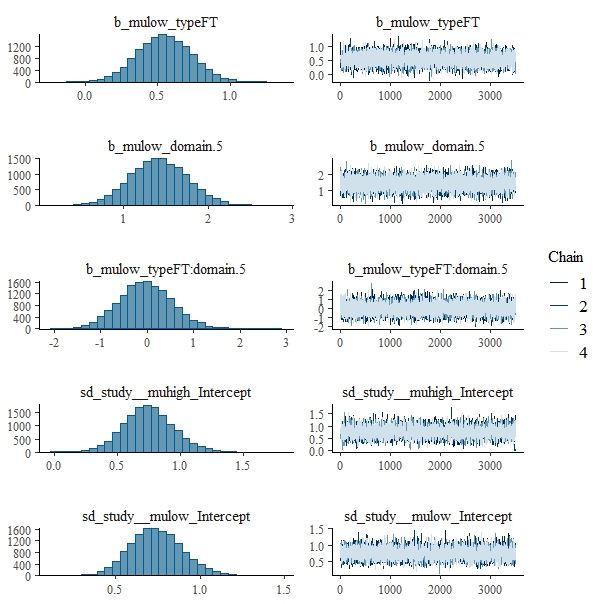


Domain 5


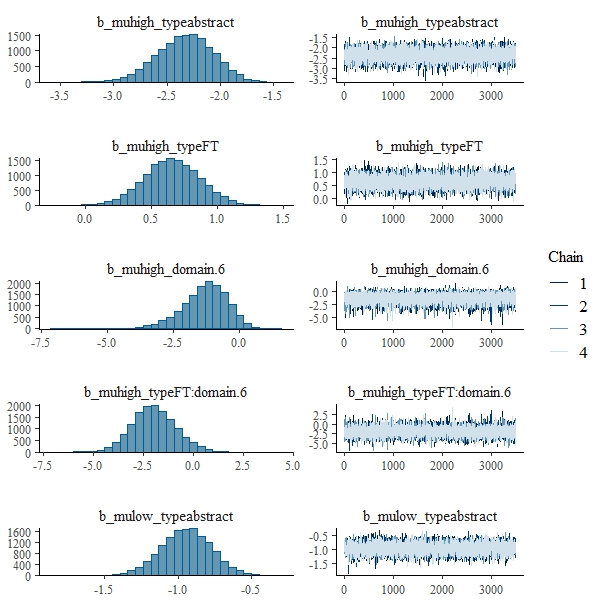

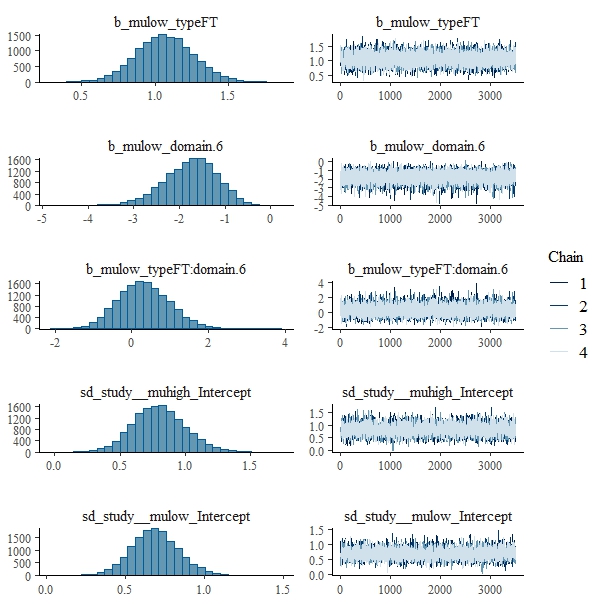


Domain 6


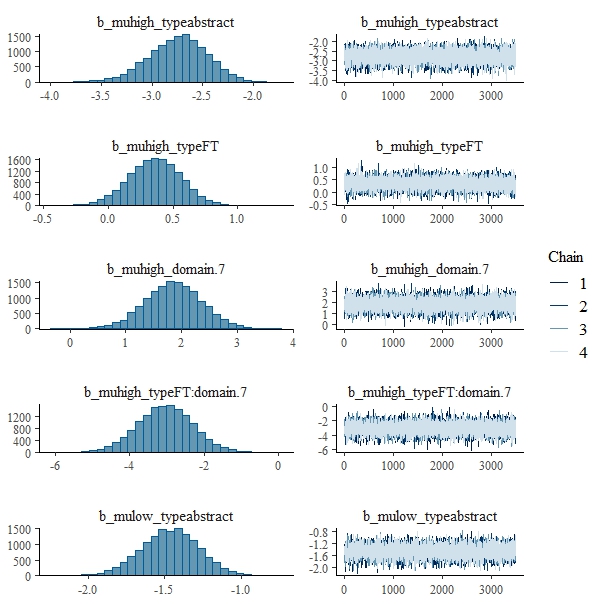

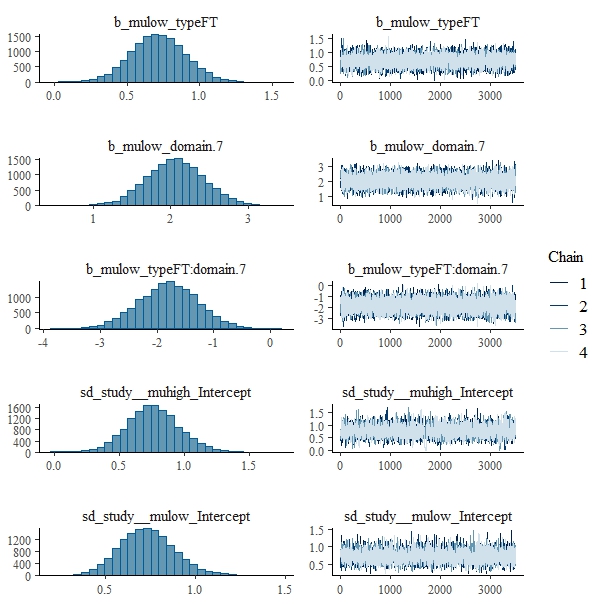


Domain 7

*Low reference*


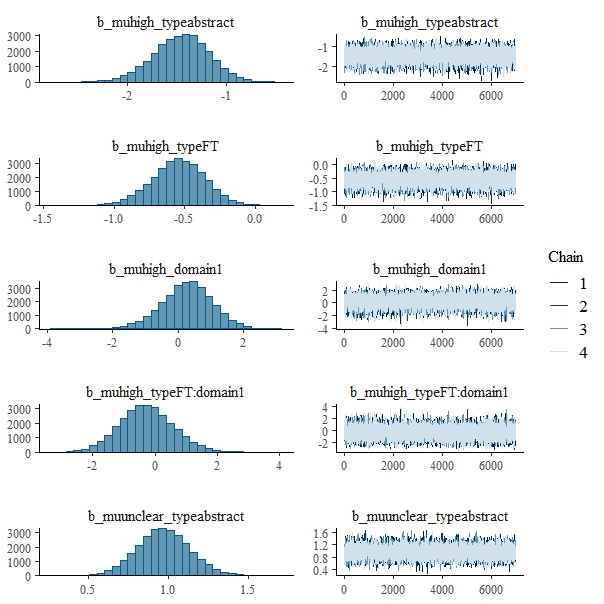

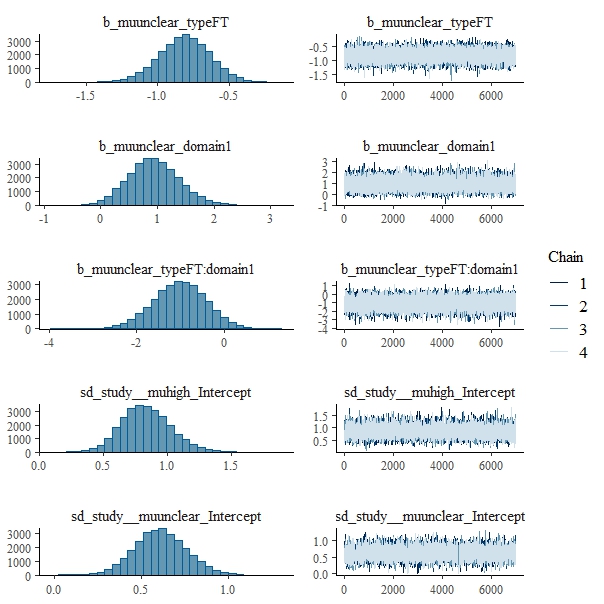


Domain 1


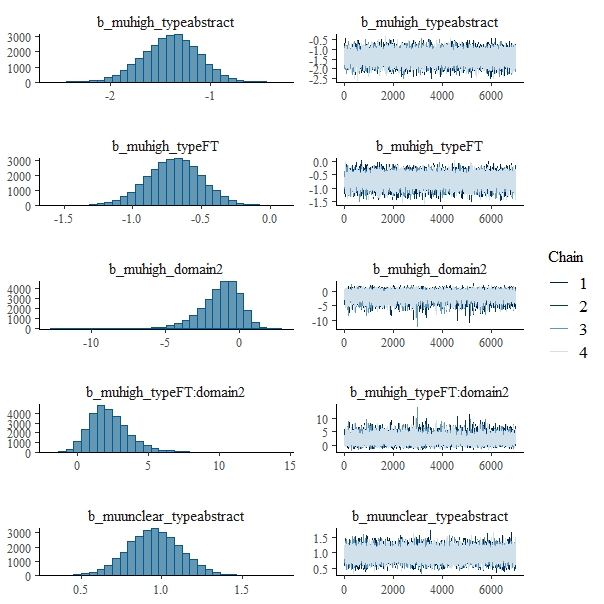

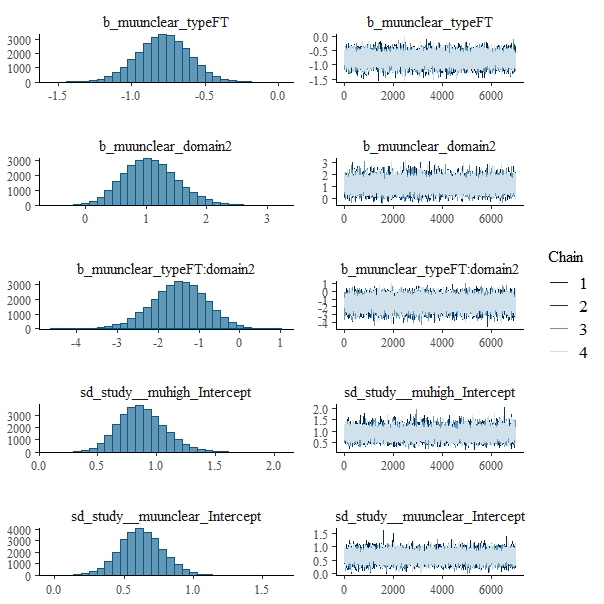


Domain 2


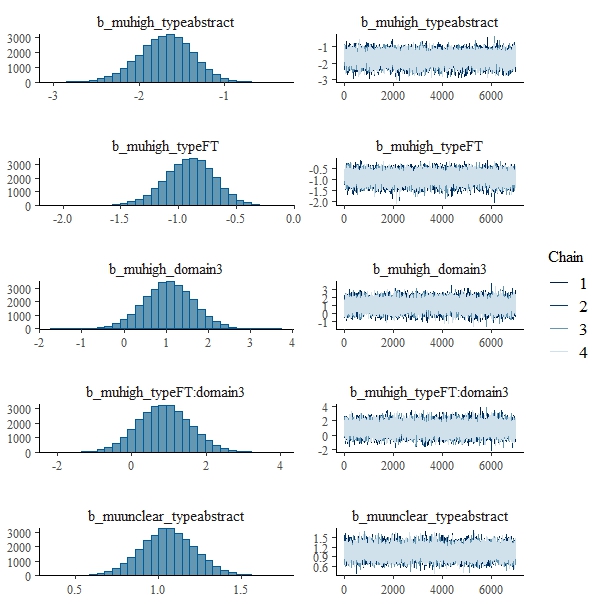

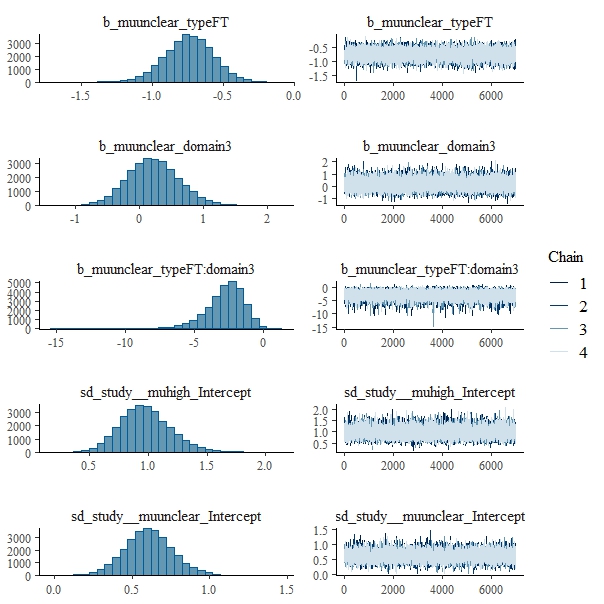


Domain 3


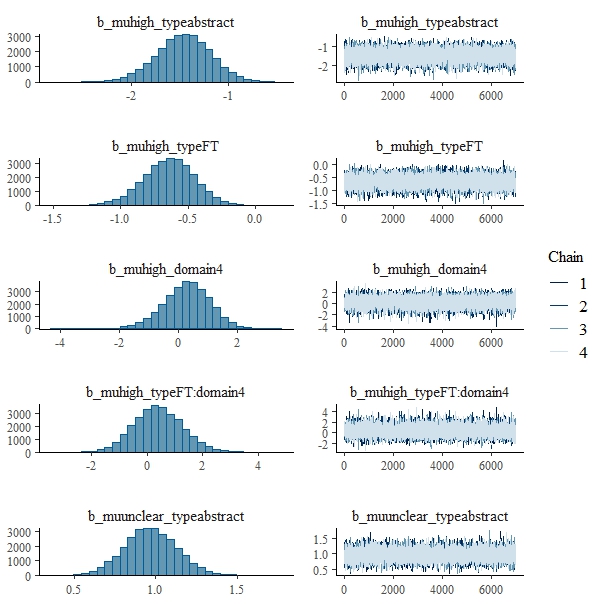

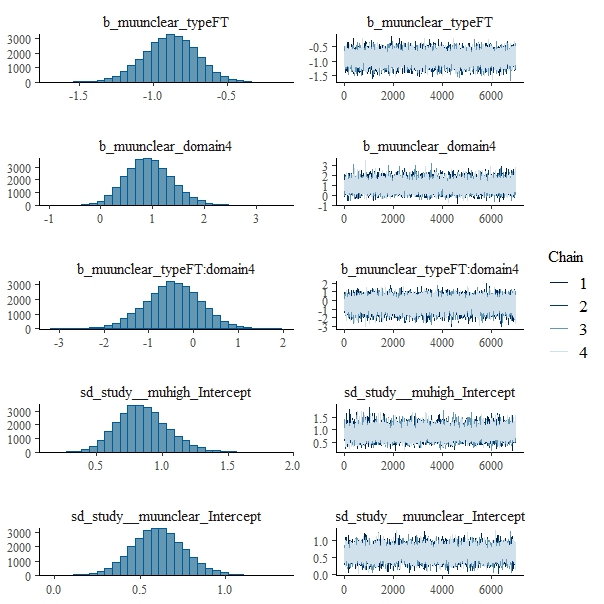


Domain 4


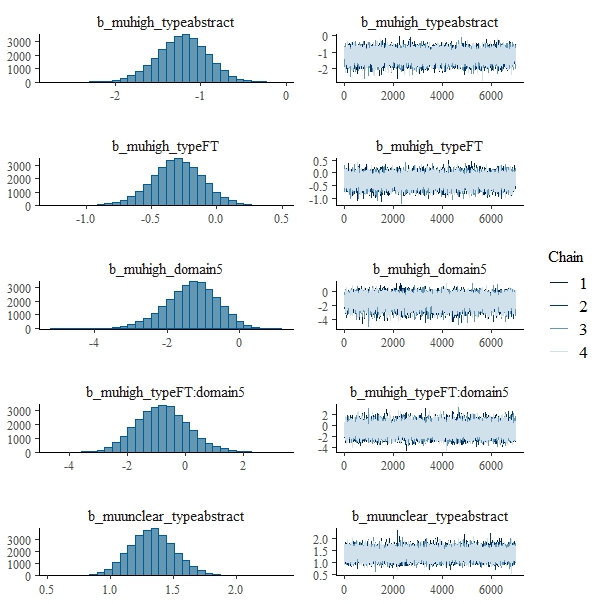

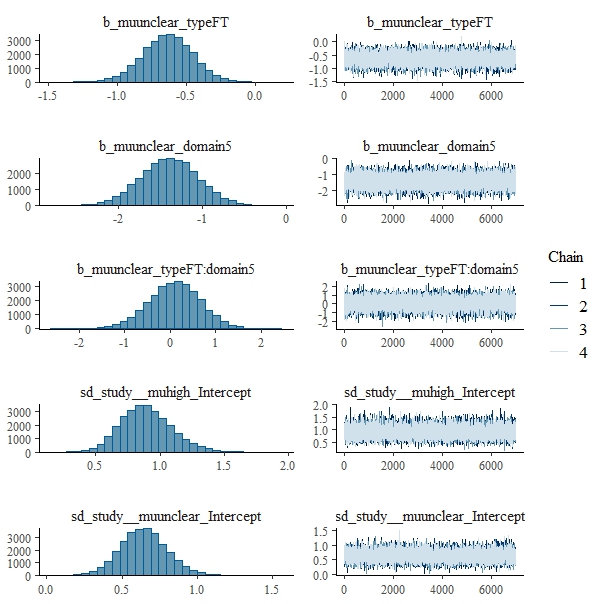


Domain 5


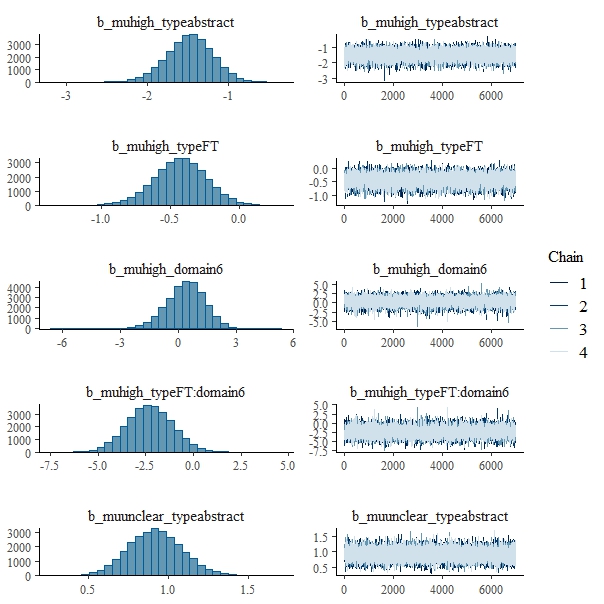

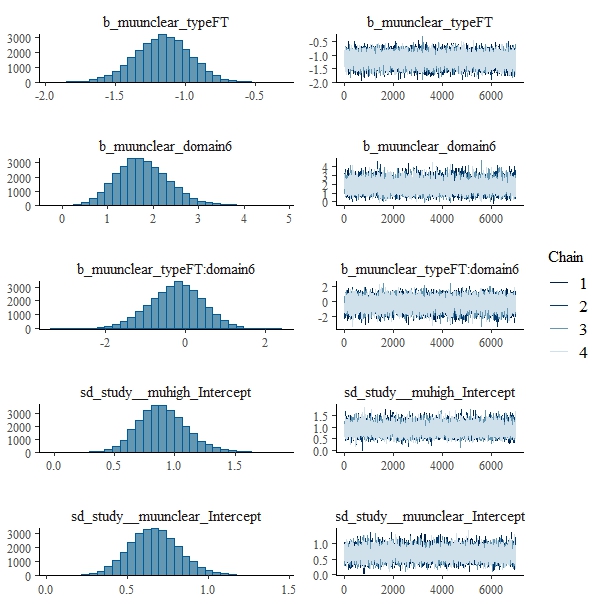


Domain 6


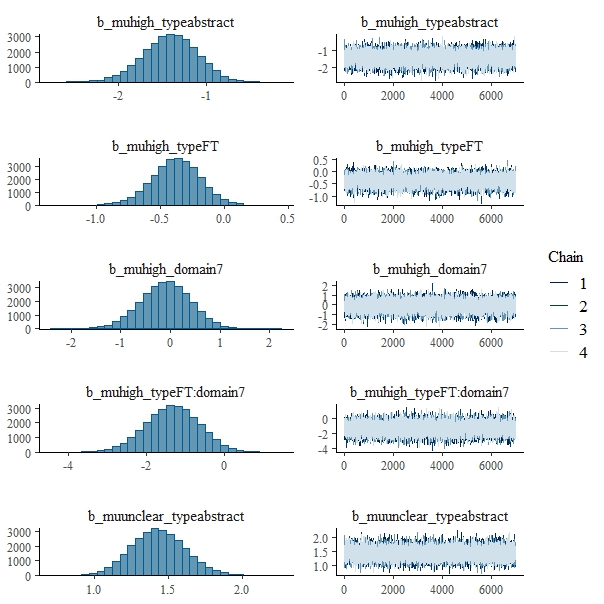

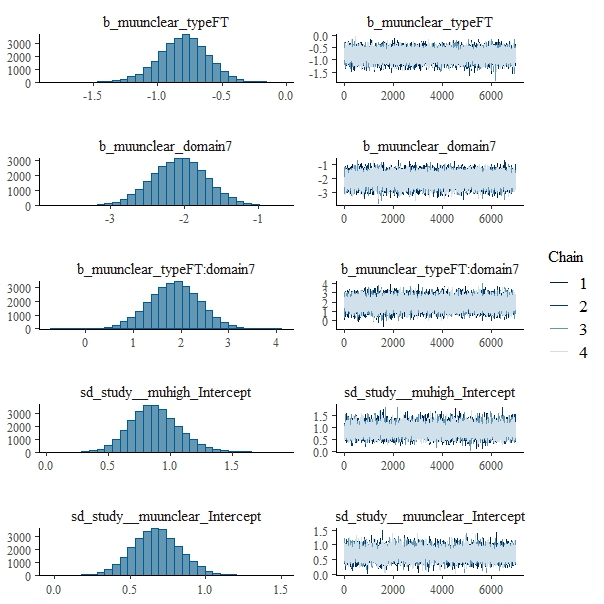


Domain 7

*High reference*


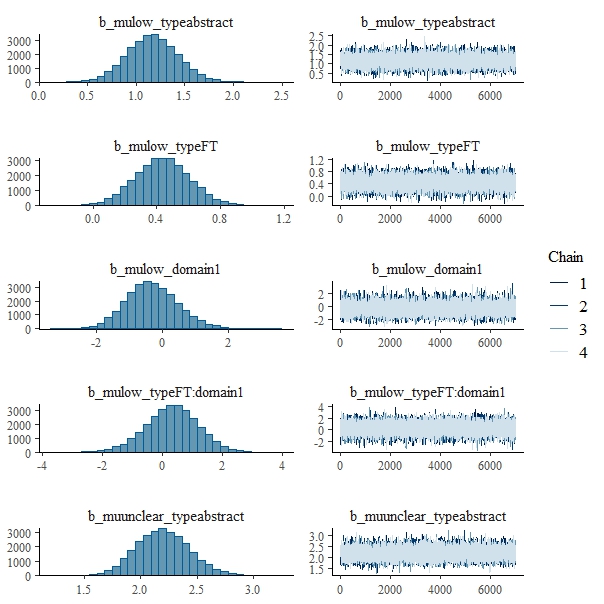

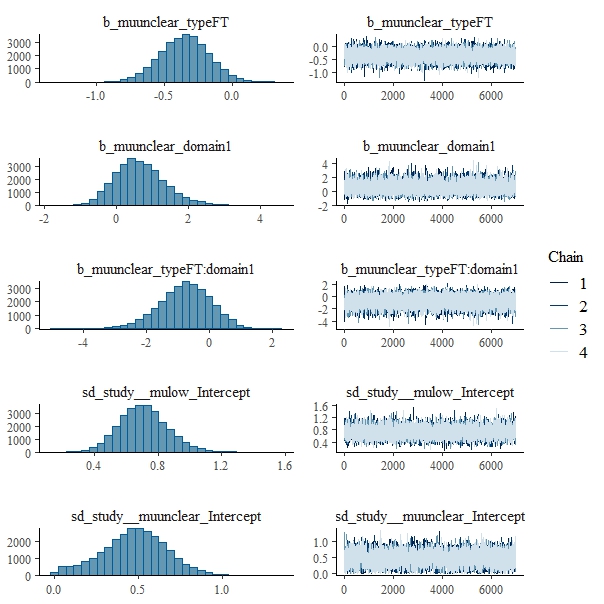


Domain 1


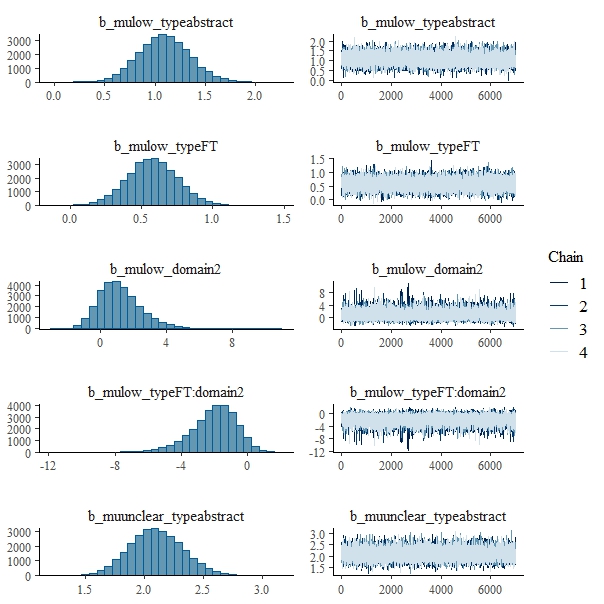

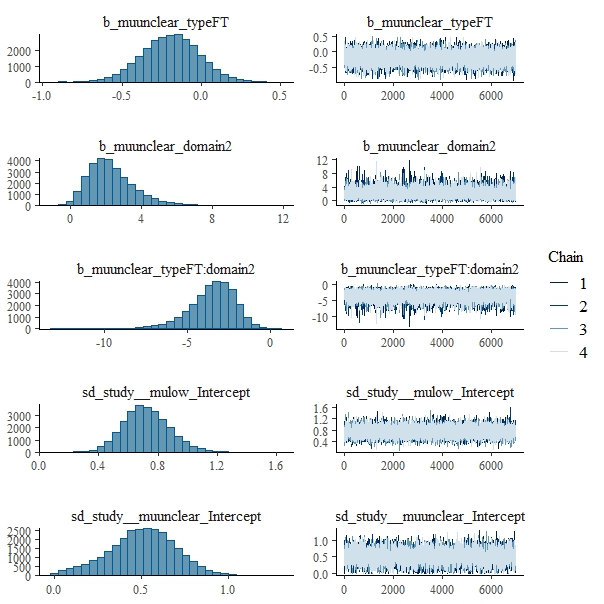


Domain 2


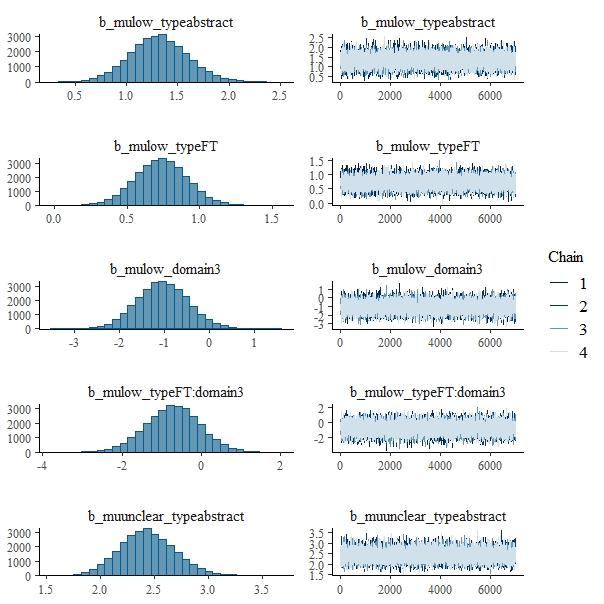

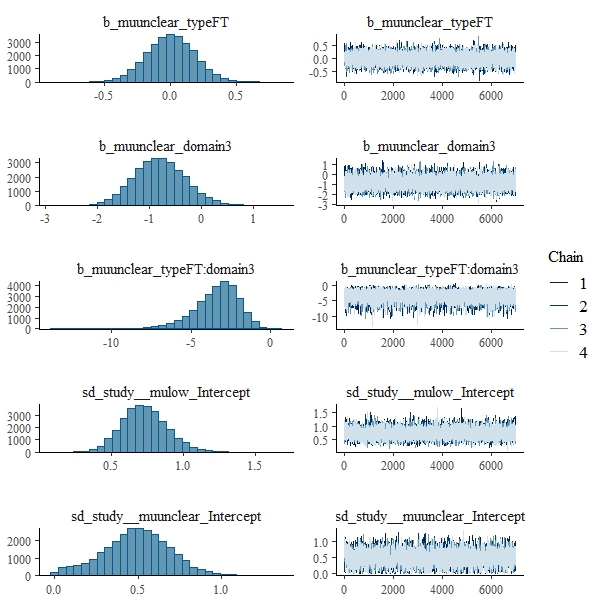


Domain 3


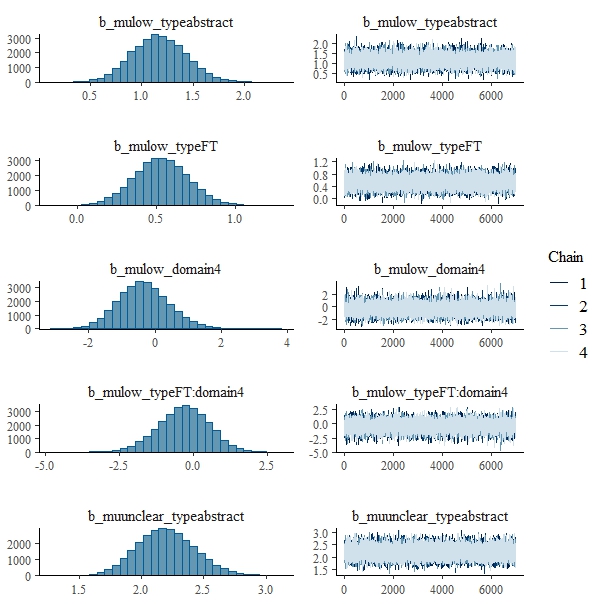

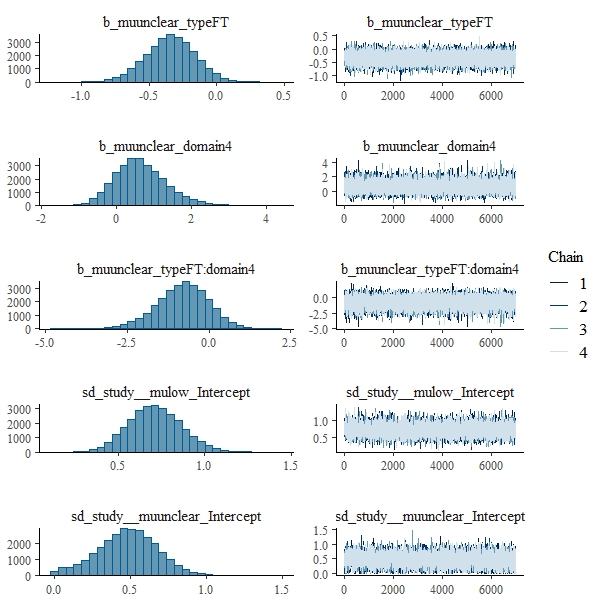


Domain 4


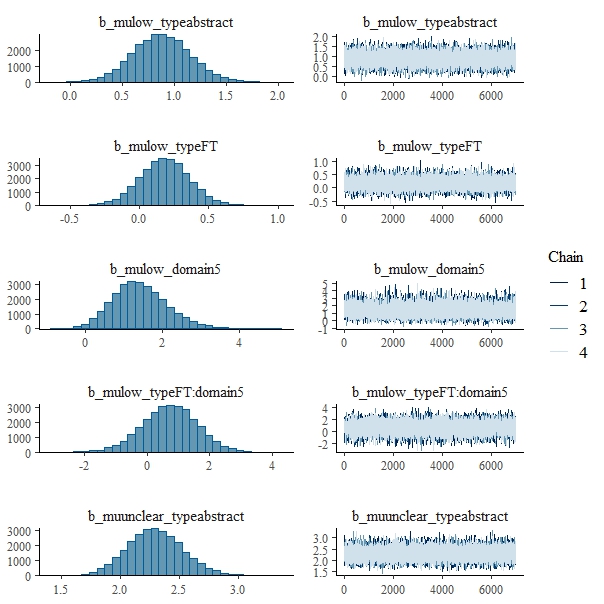

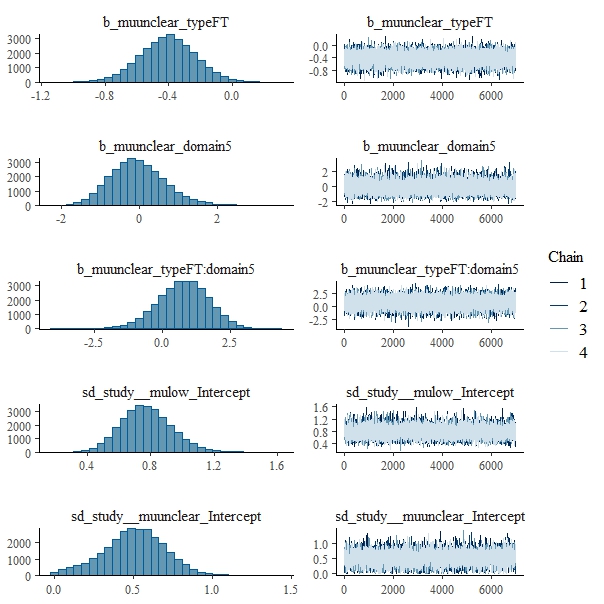


Domain 6


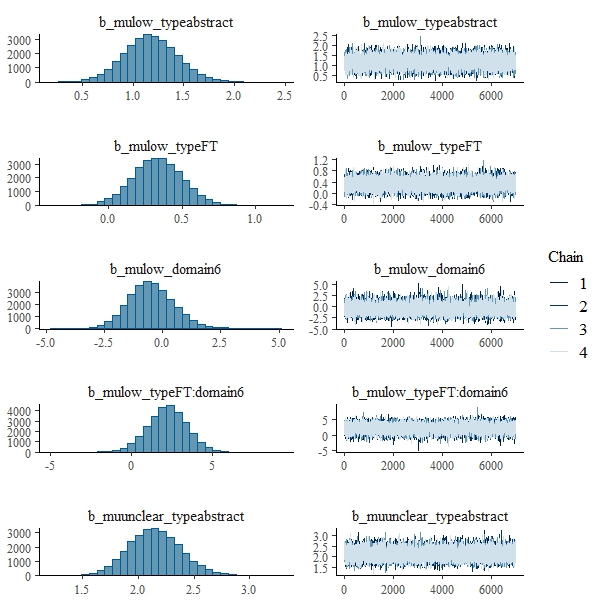

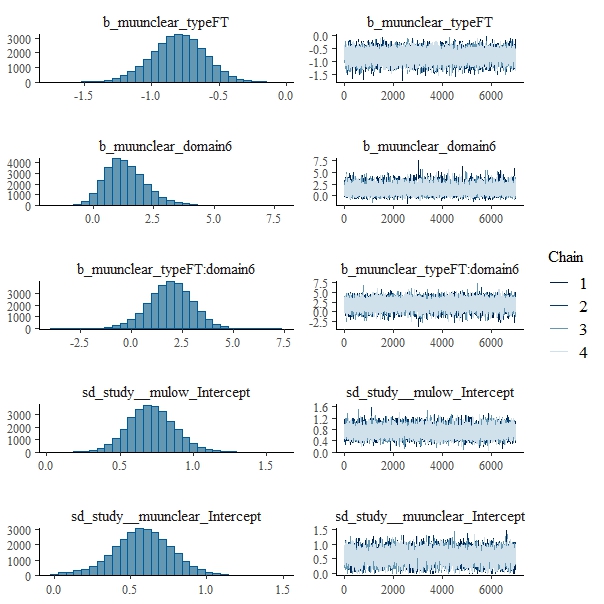


Domain 5


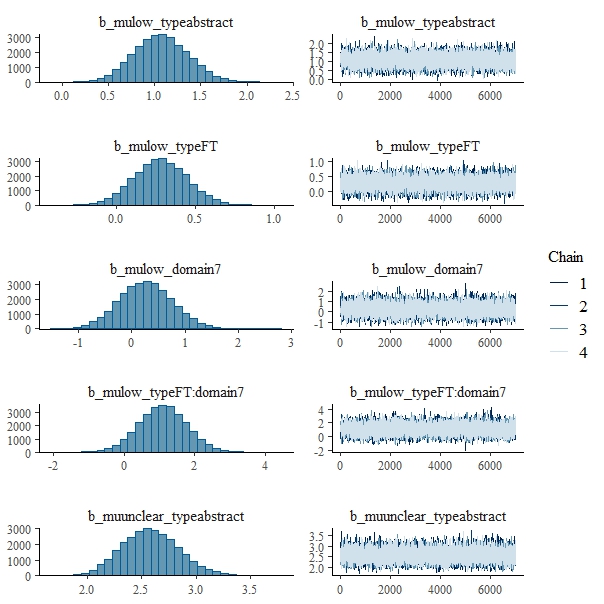

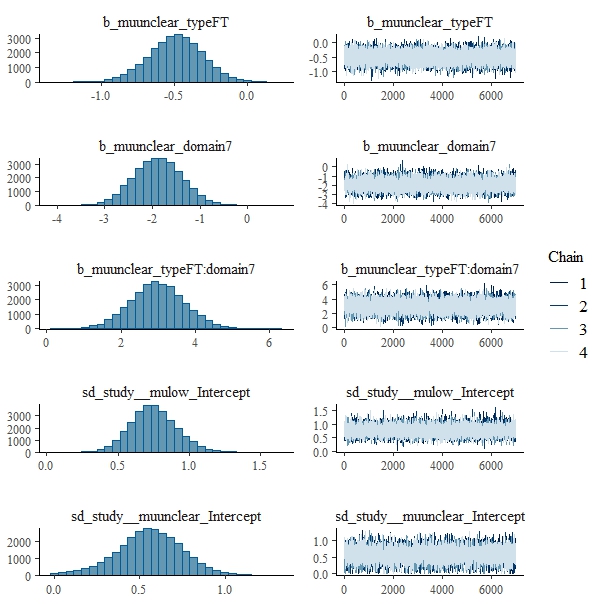


Domain 7
