# Supplementary material for: The Impact on Systematic Reviews of Risk of Bias Assessment Changes From Conference Abstracts to Full Text
Source: Cochrane Evid Synth Methods. 2026 Mar 27;4(3):e70078. doi: 10.1002/cesm.70078 (PMC13073319; doi:10.1002/cesm.70078)
Supplement: Supplementary file 3 — supplementary material 3. [file CESM-4-e70078-s003.docx]

# Absolute changes

Left column represents the abstract rating (low, unclear or high) while the following three column to the right represent the number (percentage) of ratings which were achieved at full text.

|  | | **Full text** | | | |
| --- | --- | --- | --- | --- | --- |
| **Domain** | **Abstract** | Low n (%) | Unclear n (%) | High n (%) | Total |
| 1 | Low | 6 (85.7) | 1 (14.3) | 0 (0) | 7 |
|  | Unclear | 18 (43.9) | 10 (24.4) | 13 (31.7) | 41 |
|  | High | 0 (0) | 0 (0) | 3 (100) | 3 |
| 2 | Low | 6 (85.7) | 1 (14.3) | 0 (0) | 7 |
|  | Unclear | 22 (50.0) | 6 (13.6) | 16 (36.4) | 44 |
|  | High | 0 (0) | 0 (0) | 1 (100) | 1 |
| 3 | Low | 8 (80) | 0 (0) | 2 (20) | 10 |
|  | Unclear | 6 (18.8) | 1 (3.1) | 25 (78.1) | 32 |
|  | High | 0 (0) | 0 (0) | 7 (100) | 7 |
| 4 | Low | 7 (100) | 0 (0) | 0 (0) | 7 |
|  | Unclear | 11 (26.8) | 11 (26.8) | 19 (46.3) | 41 |
|  | High | 0 (0) | 2 (66.7) | 1 (33.3) | 3 |
| 5 | Low | 21 (84) | 1 (4) | 3 (12) | 25 |
|  | Unclear | 17 (70.8) | 6 (25) | 1 (4.2) | 24 |
|  | High | 2 (66.7) | 0 (0) | 1 (33.3) | 3 |
| 6 | Low | 2 (50) | 1 (25) | 1 (25) | 4 |
|  | Unclear | 18 (40) | 25 (55.6) | 2 (4.4) | 45 |
|  | High | 0 (0) | 2 (100) | 0 (0) | 2 |
| 7 | Low | 24 (85.7) | 2 (7.1) | 2 (7.1) | 28 |
|  | Unclear | 5 (31.2) | 9 (56.2) | 2 (12.5) | 16 |
|  | High | 3 (37.5) | 2 (25) | 3 (37.5) | 8 |

For domain 1 the overall number of full texts that were downgraded form their abstract counterpart was 27.45% (e.g. moved from unclear to high), that remained the same as the abstracts was 37.25%, and finally how many were upgraded (e.g. moved from unclear to low) was 35.29%.

For domain 2 the overall number of full texts that were downgraded form their abstract counterpart was 32.69%, that remained the same as the abstracts was 25% (i.e. did not change), and finally how many were upgraded (e.g. moved from unclear to low) was 42.31%.

For domain 3 the overall number of full texts that were downgraded form their abstract counterpart was 55.1% (e.g. moved from unclear to high), that remained the same as the abstracts was 32.65% (i.e. did not change), and finally how many were upgraded (e.g. moved from unclear to low) was 12.24%.

For domain 4 the overall number of full texts that were downgraded form their abstract counterpart was 37.25% (e.g. moved from unclear to high), that remained the same as the abstracts was 37.25% (i.e. did not change), and finally how many were upgraded (e.g. moved from unclear to low) was 25.49%.

For domain 5 the overall number of full texts that were downgraded form their abstract counterpart was 9.62% (e.g. moved from unclear to high), that remained the same as the abstracts was 53.85% (i.e. did not change), and finally how many were upgraded (e.g. moved from unclear to low) was 36.54%.

For domain 6 the overall number of full texts that were downgraded form their abstract counterpart was 7.84% (e.g. moved from unclear to high), that remained the same as the abstracts was 52.94% (i.e. did not change), and finally how many were upgraded (e.g. moved from unclear to low) was 39.22%.

For domain 7 the overall number of full texts that were downgraded form their abstract counterpart was 11.54% (e.g. moved from unclear to high), that remained the same as the abstracts was 69.23% (i.e. did not change), and finally how many were upgraded (e.g. moved from unclear to low) was 19.23%.

# CONSORT statement analysis (unclear reference)

**Domain 1**

Estimate SE

elpd_waic -645.2 15.9

p_waic 43.8 1.4

waic 1290.5 31.9

Estimate Est.Error Q2.5 Q97.5

muhigh_typeabstract 0.1491880 1.266931 0.09188463 0.2327032

muhigh_typeFT 1.7711353 1.215321 1.20862428 2.6090793

muhigh_domain1 0.7037063 1.593908 0.27344701 1.7253152

muhigh_abstract_year>2008 0.4254593 1.318647 0.24412586 0.7225263

muhigh_typeFT:domain1 1.3893466 1.725275 0.48107991 4.0479990

mulow_typeabstract 0.2990223 1.209413 0.20386131 0.4294352

mulow_typeFT 1.6561126 1.214009 1.13867509 2.4392150

mulow_domain1 0.5008737 1.446461 0.23533237 1.0020271

mulow_abstract_year>2008 1.6385580 1.262977 1.03442431 2.6007034

mulow_typeFT:domain1 1.9165236 1.617896 0.74326895 4.9310059

**Domain 2**

Estimate SE

elpd_waic -641.0 15.8

p_waic 46.4 1.6

waic 1282.0 31.7

Estimate Est.Error Q2.5 Q97.5

mulow_typeabstract 0.3096634 1.211152 0.2102204 0.4466894

mulow_typeFT 1.7104728 1.215063 1.1770503 2.5282711

mulow_domain2 0.5019915 1.430495 0.2432443 0.9873696

mulow_abstract_year>2008 1.5606230 1.275863 0.9749702 2.5375858

mulow_typeFT:domain2 2.8862146 1.631987 1.1174891 7.5954761

muhigh_typeabstract 0.1502371 1.277016 0.0915897 0.2379574

muhigh_typeFT 1.5216364 1.237184 1.0019507 2.3028880

muhigh_domain2 0.4916969 1.654966 0.1756619 1.2610542

muhigh_abstract_year>2008 0.4354766 1.365615 0.2314688 0.7875573

muhigh_typeFT:domain2 3.0616923 1.793116 0.9917715 9.9059141

**Domain 3**

Estimate SE

elpd_waic -622.6 16.2

p_waic 46.2 1.6

waic 1245.2 32.3

Estimate Est.Error Q2.5 Q97.5

muhigh_typeabstract 0.1198881 1.296214 0.07054075 0.1949693

muhigh_typeFT 1.2961654 1.233369 0.85364529 1.9518178

muhigh_domain3 2.4945546 1.504080 1.10122858 5.4873546

muhigh_abstract_year>2008 0.3902805 1.345128 0.21776536 0.6938952

muhigh_typeFT:domain3 5.0982970 1.787953 1.68366378 16.3990520

mulow_typeabstract 0.2786181 1.212177 0.18881751 0.4038483

mulow_typeFT 1.6096405 1.213419 1.10109331 2.3571638

mulow_domain3 0.9072878 1.417187 0.45748285 1.7743248

mulow_abstract_year>2008 1.6304804 1.266849 1.02430506 2.6041168

mulow_typeFT:domain3 2.0706249 1.755973 0.68935188 6.3070472

**Domain 4**

Estimate SE

elpd_waic -643.0 16.0

p_waic 44.2 1.5

waic 1286.1 32.1

Estimate Est.Error Q2.5 Q97.5

muhigh_typeabstract 0.1485799 1.269191 0.09098188 0.2314150

muhigh_typeFT 1.7292083 1.219329 1.16668677 2.5406834

muhigh_domain4 0.7270103 1.588055 0.28732565 1.7440739

muhigh_abstract_year>2008 0.4240351 1.322159 0.24457461 0.7262716

muhigh_typeFT:domain4 1.5644848 1.720325 0.55067129 4.6610342

mulow_typeabstract 0.3008047 1.207660 0.20455782 0.4315959

mulow_typeFT 1.7717908 1.215906 1.21133268 2.6090125

mulow_domain4 0.4704470 1.444063 0.22437976 0.9520647

mulow_abstract_year>2008 1.6478519 1.267389 1.03999324 2.6379742

mulow_typeFT:domain4 1.2027991 1.617566 0.47061123 3.0807443

**Domain 5**

Estimate SE

elpd_waic -624.5 16.8

p_waic 46.1 1.7

waic 1249.0 33.7

1 (0.1%) p_waic estimates greater than 0.4.

LOO

Estimate SE

elpd_loo -624.7 16.9

p_loo 46.3 1.7

looic 1249.5 33.7

All Pareto k estimates are good (k < 0.7).

Estimate Est.Error Q2.5 Q97.5

muhigh_typeabstract 0.1451189 1.268759 0.08917491 0.2265612

muhigh_typeFT 1.8635330 1.219756 1.26152606 2.7536059

muhigh_domain5 0.8061921 1.685133 0.27119173 2.1691492

muhigh_abstract_year>2008 0.4169145 1.323076 0.23885752 0.7144049

muhigh_typeFT:domain5 0.6285445 1.906090 0.17914934 2.2633821

mulow_typeabstract 0.2205855 1.228060 0.14628355 0.3306652

mulow_typeFT 1.3447912 1.229117 0.89704989 2.0108092

mulow_domain5 3.4159551 1.352862 1.87253081 6.1568881

mulow_abstract_year>2008 1.6632175 1.284214 1.02079054 2.7266668

mulow_typeFT:domain5 1.2045155 1.600189 0.48553411 3.0676615

**Domain 6**

Estimate SE

elpd_waic -619.7 16.5

p_waic 45.2 1.6

waic 1239.5 33.1

3 (0.4%) p_waic estimates greater than 0.4.

LOO

Estimate SE

elpd_loo -620.0 16.5

p_loo 45.4 1.6

looic 1239.9 33.1

All Pareto k estimates are good (k < 0.7).

Estimate Est.Error Q2.5 Q97.5

muhigh_typeabstract 0.1603061 1.268602 0.09823509 0.2501538

muhigh_typeFT 2.5213214 1.232748 1.67830910 3.7859102

muhigh_domain6 0.2805942 1.724966 0.09244626 0.7797340

muhigh_abstract_year>2008 0.4195739 1.331576 0.23669649 0.7310207

muhigh_typeFT:domain6 0.2527156 1.881031 0.07322692 0.8814777

mulow_typeabstract 0.3171147 1.210628 0.21691837 0.4576346

mulow_typeFT 2.1350819 1.227838 1.42872803 3.1980191

mulow_domain6 0.2603190 1.513385 0.11058934 0.5671395

mulow_abstract_year>2008 1.6392068 1.269576 1.02297583 2.6327675

mulow_typeFT:domain6 1.0558781 1.637243 0.41077102 2.8650378

**Domain 7**

Estimate SE

elpd_waic -625.6 16.5

p_waic 45.9 1.7

waic 1251.3 33.1

1 (0.1%) p_waic estimates greater than 0.4

LOO

Estimate SE

elpd_loo -625.8 16.5

p_loo 46.2 1.7

looic 1251.7 33.1

All Pareto k estimates are good (k < 0.7)

Estimate Est.Error Q2.5 Q97.5

muhigh_typeabstract 0.1263825 1.277935 0.07678009 0.2021237

muhigh_typeFT 1.8844877 1.219647 1.26936585 2.7943620

muhigh_domain7 2.4275271 1.553844 1.02010835 5.6869483

muhigh_abstract_year>2008 0.4233492 1.327238 0.23813985 0.7281604

muhigh_typeFT:domain7 0.2159051 1.755246 0.07285351 0.6592350

mulow_typeabstract 0.2123107 1.224809 0.14115613 0.3108812

mulow_typeFT 1.5072618 1.222726 1.02310376 2.2343205

mulow_domain7 4.6247628 1.366475 2.47581943 8.5648042

mulow_abstract_year>2008 1.6446575 1.277830 1.01692273 2.6746760

mulow_typeFT:domain7 0.3887369 1.568751 0.16169223 0.9427128

# CONSORT statement analysis (high reference)

**Domain 1**

Estimate SE

elpd_waic -645.1 16.0

p_waic 42.9 1.3

waic 1290.1 32.0

Estimate Est.Error Q2.5 Q97.5

muunclear_typeabstract 6.3471499 1.252102 4.1525192 10.0485292

muunclear_typeFT 0.5399148 1.222592 0.3597459 0.7946034

muunclear_domain1 1.6410590 1.523935 0.7468804 3.8460012

muunclear_abstract_year>2008 2.0974277 1.311028 1.2337295 3.5577814

muunclear_typeFT:domain1 0.5922362 1.682155 0.2116752 1.6251305

mulow_typeabstract 1.8313635 1.280117 1.1221015 2.9803320

mulow_typeFT 0.9400782 1.201103 0.6554639 1.3471912

mulow_domain1 0.7282291 1.607989 0.2903200 1.8561261

mulow_abstract_year>2008 3.4882679 1.303214 2.0743042 5.8605971

mulow_typeFT:domain1 1.3535610 1.701022 0.4806964 3.8229422

**Domain 2**

Estimate SE

elpd_waic -641.1 16.0

p_waic 43.9 1.4

waic 1282.1 32.0

Estimate Est.Error Q2.5 Q97.5

mulow_typeabstract 1.8364268 1.282147 1.13145544 2.982237

mulow_typeFT 1.0817224 1.202960 0.75365611 1.549288

mulow_domain2 0.8460335 1.613251 0.33396175 2.158103

mulow_abstract_year>2008 3.3828384 1.320027 1.99029948 5.916312

mulow_typeFT:domain2 1.2038719 1.699788 0.42364554 3.392427

muunclear_typeabstract 6.1342238 1.260367 3.92416040 9.707618

muunclear_typeFT 0.6123818 1.229056 0.40383188 0.909455

muunclear_domain2 2.1863225 1.546418 0.94102318 5.177029

muunclear_abstract_year>2008 2.1232436 1.334043 1.21397138 3.747634

muunclear_typeFT:domain2 0.2715932 1.730705 0.09027725 0.784744

**Domain 3**

Estimate SE

elpd_waic -623.8 16.1

p_waic 42.7 1.4

waic 1247.5 32.2

1 (0.1%) p_waic estimates greater than 0.4

LOO

Estimate SE

elpd_loo -623.9 16.1

p_loo 42.9 1.4

looic 1247.9 32.2

All Pareto k estimates are good (k < 0.7).

Estimate Est.Error Q2.5 Q97.5

muunclear_typeabstract 7.6912982 1.263673 4.95545888 12.2337775

muunclear_typeFT 0.7202431 1.219271 0.48269434 1.0549303

muunclear_domain3 0.4363142 1.455034 0.21164056 0.9216993

muunclear_abstract_year>2008 2.2334806 1.313203 1.31021189 3.8227893

muunclear_typeFT:domain3 0.1658989 1.846042 0.04760685 0.5288145

mulow_typeabstract 2.0415069 1.289410 1.25029629 3.3669016

mulow_typeFT 1.1948461 1.206567 0.82840896 1.7290173

mulow_domain3 0.3787433 1.523395 0.16603309 0.8514284

mulow_abstract_year>2008 3.7043325 1.314388 2.17074358 6.3979873

mulow_typeFT:domain3 0.5195155 1.647746 0.19416114 1.3771413

**Domain 4**

Estimate SE

elpd_waic -643.3 16.1

p_waic 43.0 1.3

waic 1286.6 32.2

Estimate Est.Error Q2.5 Q97.5

muunclear_typeabstract 6.3990342 1.252440 4.1602420 10.0289217

muunclear_typeFT 0.5481509 1.219796 0.3669078 0.8017393

muunclear_domain4 1.4966544 1.517440 0.6791498 3.5226787

muunclear_abstract_year>2008 2.1065322 1.305710 1.2569554 3.5842600

muunclear_typeFT:domain4 0.6071775 1.658832 0.2230617 1.6305279

mulow_typeabstract 1.8446448 1.279909 1.1324033 3.0082551

mulow_typeFT 1.0171809 1.201730 0.7040398 1.4469328

mulow_domain4 0.6267532 1.609692 0.2450706 1.5877930

mulow_abstract_year>2008 3.5358807 1.301488 2.1191448 5.9907862

mulow_typeFT:domain4 0.8444385 1.710964 0.2936107 2.4352861

**Domain 5**

Estimate SE

elpd_waic -625.0 16.9

p_waic 44.5 1.5

waic 1250.0 33.8

Estimate Est.Error Q2.5 Q97.5

muunclear_typeabstract 6.8570352 1.255821 4.4305816 10.8708932

muunclear_typeFT 0.5237200 1.214696 0.3552663 0.7638039

muunclear_domain5 0.9525391 1.545355 0.4141085 2.2723161

muunclear_abstract_year>2008 2.1165944 1.313070 1.2377515 3.6425424

muunclear_typeFT:domain5 1.6116961 1.780676 0.5152209 4.9941843

mulow_typeabstract 1.3902271 1.297617 0.8350742 2.3248417

mulow_typeFT 0.7365617 1.210163 0.5072024 1.0751984

mulow_domain5 3.6814637 1.559098 1.5538635 8.9729038

mulow_abstract_year>2008 3.6075092 1.313530 2.1139246 6.2167242

mulow_typeFT:domain5 1.7651003 1.695651 0.6227748 4.9701001

**Domain 6**

Estimate SE

elpd_waic -619.9 16.6

p_waic 44.1 1.5

waic 1239.8 33.1

Estimate Est.Error Q2.5 Q97.5

muunclear_typeabstract 5.8772467 1.260142 3.7751105 9.3185009

muunclear_typeFT 0.3685794 1.241899 0.2380128 0.5542262

muunclear_domain6 4.6371803 1.647705 1.7913109 12.6578039

muunclear_abstract_year>2008 2.0863825 1.332110 1.1796432 3.6486398

muunclear_typeFT:domain6 2.6880207 1.777405 0.8679688 8.2781840

mulow_typeabstract 1.7940508 1.280468 1.1070465 2.9360652

mulow_typeFT 0.8792068 1.195247 0.6192366 1.2452715

mulow_domain6 1.0541907 1.717141 0.3681595 3.0797445

mulow_abstract_year>2008 3.4632993 1.299756 2.0681470 5.8198539

mulow_typeFT:domain6 2.7212938 1.805681 0.8561516 8.7681548

**Domain 7**

Estimate SE

elpd_waic -624.2 16.7

p_waic 45.3 1.6

waic 1248.5 33.4

Estimate Est.Error Q2.5 Q97.5

muunclear_typeabstract 7.8405297 1.260248 5.0459124 12.4874834

muunclear_typeFT 0.5025690 1.221288 0.3379625 0.7392139

muunclear_domain7 0.3363211 1.494108 0.1545894 0.7439451

muunclear_abstract_year>2008 2.0987609 1.317554 1.2163665 3.6472448

muunclear_typeFT:domain7 5.5773708 1.682837 2.0053350 15.4120300

mulow_typeabstract 1.4658786 1.296685 0.8769762 2.4405451

mulow_typeFT 0.8182408 1.201716 0.5678793 1.1722225

mulow_domain7 2.1877265 1.494931 1.0072741 4.8727542

mulow_abstract_year>2008 3.5372688 1.308011 2.1152230 6.0234557

mulow_typeFT:domain7 1.3177483 1.631128 0.5102770 3.4494161

# CONSORT statement analysis (low reference)

**Domain 1**

Estimate SE

elpd_waic -644.9 16.1

p_waic 46.2 1.6

waic 1289.8 32.1

2 (0.3%) p_waic estimates greater than 0.4.

LOO

Estimate SE

elpd_loo -645.1 16.1

p_loo 46.4 1.6

looic 1290.2 32.1

All Pareto k estimates are good (k < 0.7).

Estimate Est.Error Q2.5 Q97.5

muhigh_typeabstract 0.4569902 1.293775 0.2728011 0.7521715

muhigh_typeFT 1.0356430 1.206499 0.7164646 1.4990849

muhigh_domain1 1.0628455 1.676519 0.3751454 2.8172682

muhigh_abstract_year>2008 0.2756401 1.316186 0.1587601 0.4656879

muhigh_typeFT:domain1 0.9851176 1.767545 0.3290357 3.0676020

muunclear_typeabstract 3.2965422 1.209444 2.2814668 4.8542432

muunclear_typeFT 0.5508747 1.224534 0.3673185 0.8144994

muunclear_domain1 1.9856947 1.420841 1.0126609 4.0144649

muunclear_abstract_year>2008 0.6319302 1.269634 0.3954802 1.0075147

muunclear_typeFT:domain1 0.5137811 1.616225 0.1950281 1.2989085

**Domain 2**

Estimate SE

elpd_waic -638.9 16.0

p_waic 48.7 1.7

waic 1277.8 32.1

2 (0.3%) p_waic estimates greater than 0.4.

LOO Estimate SE

elpd_loo -639.2 16.1

p_loo 48.9 1.7

looic 1278.3 32.1

All Pareto k estimates are good (k < 0.7).

Estimate Est.Error Q2.5 Q97.5

muhigh_typeabstract 0.4542148 1.303882 0.26599882 0.7485849

muhigh_typeFT 0.8804879 1.219952 0.59143820 1.2917823

muhigh_domain2 0.6589546 1.748584 0.21501752 1.9127319

muhigh_abstract_year>2008 0.2834352 1.361853 0.15197756 0.5113449

muhigh_typeFT:domain2 1.6240268 1.824108 0.50720728 5.3229053

muunclear_typeabstract 3.2120638 1.211881 2.22424476 4.7060538

muunclear_typeFT 0.5555746 1.225479 0.37038150 0.8253596

muunclear_domain2 2.2370636 1.424390 1.13775511 4.5294396

muunclear_abstract_year>2008 0.6384556 1.282378 0.39431041 1.0425728

muunclear_typeFT:domain2 0.2665836 1.643373 0.09924931 0.6914347

muunclear_typeFT:domain2 0.3337903 1.660894 0.1198639 0.8762152

**Domain 3**

Estimate SE

elpd_waic -621.5 16.4

p_waic 48.4 1.9

waic 1243.1 32.8

4 (0.6%) p_waic estimates greater than 0.4.

LOO Estimate SE

elpd_loo -621.8 16.4

p_loo 48.6 1.9

looic 1243.6 32.9

All Pareto k estimates are good (k < 0.7).

Estimate Est.Error Q2.5 Q97.5

muhigh_typeabstract 0.3889318 1.324676 0.21813358 0.6657526

muhigh_typeFT 0.7817262 1.223329 0.52145484 1.1555187

muhigh_domain3 2.3874557 1.548309 1.00768484 5.5825150

muhigh_abstract_year>2008 0.2531048 1.344847 0.14026330 0.4467200

muhigh_typeFT:domain3 2.6649160 1.675961 0.98259392 7.3233909

muunclear_typeabstract 3.5703657 1.211824 2.44756170 5.2265484

muunclear_typeFT 0.5864962 1.218041 0.39288431 0.8538051

muunclear_domain3 1.0250259 1.397028 0.53818184 2.0053975

muunclear_abstract_year>2008 0.6340364 1.275557 0.39250442 1.0222155

muunclear_typeFT:domain3 0.2826547 1.902450 0.07724795 0.9451983

**Domain 4**

Estimate SE

elpd_waic -642.9 16.2

p_waic 46.4 1.6

waic 1285.8 32.3

2 (0.3%) p_waic estimates greater than 0.4.

LOO Estimate SE

elpd_loo -643.1 16.2

p_loo 46.6 1.7

looic 1286.2 32.3

All Pareto k estimates are good (k < 0.7).

Estimate Est.Error Q2.5 Q97.5

muhigh_typeabstract 0.4476522 1.296210 0.2646609 0.7410429

muhigh_typeFT 0.9543617 1.205693 0.6590156 1.3654082

muhigh_domain4 1.2692900 1.671419 0.4558784 3.4303289

muhigh_abstract_year>2008 0.2747330 1.317499 0.1572879 0.4662263

muhigh_typeFT:domain4 1.5076368 1.758837 0.5045593 4.6326258

muunclear_typeabstract 3.2716669 1.210663 2.2723010 4.8152650

muunclear_typeFT 0.5190163 1.223296 0.3453299 0.7669910

muunclear_domain4 2.1432545 1.428652 1.0849031 4.3686028

muunclear_abstract_year>2008 0.6291836 1.269539 0.3940827 1.0055159

muunclear_typeFT:domain4 0.7664033 1.608461 0.2956030 1.9154183

**Domain 5**

Estimate SE

elpd_waic -625.2 16.9

p_waic 47.7 1.8

waic 1250.5 33.7

1 (0.1%) p_waic estimates greater than 0.4

LOO Estimate SE

elpd_loo -625.4 16.9

p_loo 47.9 1.8

looic 1250.9 33.8

All Pareto k estimates are good (k < 0.7).

Estimate Est.Error Q2.5 Q97.5

muhigh_typeabstract 0.5485078 1.310498 0.3161305 0.9214220

muhigh_typeFT 1.3174118 1.215919 0.8932543 1.9243845

muhigh_domain5 0.3356800 1.630726 0.1245153 0.8420450

muhigh_abstract_year>2008 0.2690922 1.329388 0.1539006 0.4668766

muhigh_typeFT:domain5 0.4403055 1.792054 0.1404407 1.3766927

muunclear_typeabstract 4.4164104 1.221856 3.0128456 6.5785755

muunclear_typeFT 0.6626366 1.227425 0.4423219 0.9824833

muunclear_domain5 0.2959955 1.350156 0.1642879 0.5294573

muunclear_abstract_year>2008 0.6260028 1.276708 0.3846409 1.0067572

muunclear_typeFT:domain5 0.9456660 1.610871 0.3664179 2.3793031

**Domain 6**

Estimate SE

elpd_waic -618.3 16.9

p_waic 48.4 1.9

waic 1236.7 33.8

5 (0.7%) p_waic estimates greater than 0.4.

LOO Estimate SE

elpd_loo -618.6 16.9

p_loo 48.6 1.9

looic 1237.1 33.8

All Pareto k estimates are good (k < 0.7).

Estimate Est.Error Q2.5 Q97.5

muhigh_typeabstract 0.4625900 1.295933 0.27228315 0.7578516

muhigh_typeFT 1.1498267 1.205794 0.79201074 1.6491182

muhigh_domain6 0.6702244 1.829124 0.19507907 2.0965936

muhigh_abstract_year>2008 0.2708642 1.323540 0.15449849 0.4617792

muhigh_typeFT:domain6 0.3552570 1.966476 0.09382914 1.3641136

muunclear_typeabstract 3.1222111 1.216077 2.13433140 4.5809379

muunclear_typeFT 0.3991857 1.240665 0.25641790 0.6006262

muunclear_domain6 3.6271181 1.478844 1.74217278 7.9933834

muunclear_abstract_year>2008 0.6361518 1.281229 0.38967803 1.0340362

muunclear_typeFT:domain6 1.2405063 1.597623 0.48739428 3.0638078

**Domain 7**

Estimate SE

elpd_waic -623.1 16.6

p_waic 48.5 1.8

waic 1246.1 33.2

Estimate Est.Error Q2.5 Q97.5

muhigh_typeabstract 0.4785677 1.324203 0.27195808 0.8156855

muhigh_typeFT 1.2114844 1.209164 0.83441811 1.7636467

muhigh_domain7 0.8228417 1.527677 0.35181018 1.8633157

muhigh_abstract_year>2008 0.2722162 1.324131 0.15377797 0.4709208

muhigh_typeFT:domain7 0.3489151 1.705970 0.11963494 0.9946808

muunclear_typeabstract 4.7158635 1.226751 3.18591670 7.0876696

muunclear_typeFT 0.5770611 1.233986 0.37554332 0.8591127

muunclear_domain7 0.1828961 1.374063 0.09804179 0.3400845

muunclear_abstract_year>2008 0.6361583 1.282016 0.39201608 1.0427544

muunclear_typeFT:domain7 3.7197316 1.570150 1.52433560 8.9036128

# CONSORT statement analysis (interaction term; unclear reference)

This analysis includes interactions between abstract year (≤2008 vs >2008), type (abstract vs full text), and domain.

**Domain 1**

Estimate SE

elpd_waic -638.5 15.6

p_waic 47.7 1.6

waic 1276.9 31.1

Estimate Est.Error Q2.5 Q97.5

mulow_typeabstract 0.3245884 1.224692 0.21648567 0.4794364

mulow_typeFT 1.6621838 1.245938 1.07757735 2.5539413

mulow_domain1 0.4353480 1.533693 0.18315830 0.9645408

mulow_abstract_year>2008 1.3912933 1.335692 0.78953422 2.4796220

mulow_typeFT:domain1 1.8666333 1.634770 0.73139390 4.9952486

mulow_domain1:abstract_year>2008 1.3105360 1.612479 0.50926248 3.3523817

mulow_typeFT:abstract_year>2008 1.1668431 1.418657 0.59273561 2.3418154

muhigh_typeabstract 0.1158282 1.303722 0.06799631 0.1909459

muhigh_typeFT 1.8860615 1.237594 1.24142134 2.8810925

muhigh_domain1 0.9420001 1.647014 0.34369007 2.4144405

muhigh_abstract_year>2008 0.9630758 1.468073 0.44871675 2.0223994

muhigh_typeFT:domain1 1.3940136 1.784513 0.46430015 4.4497512

muhigh_domain1:abstract_year>2008 0.1853870 2.004932 0.04414840 0.6780293

muhigh_typeFT:abstract_year>2008 0.3585236 1.556044 0.14949584 0.8488340

**Domain 2**

Estimate SE

elpd_waic -628.5 15.8

p_waic 48.1 1.7

waic 1257.0 31.7

1 (0.1%) p_waic estimates greater than 0.4.

LOO Estimate SE

elpd_loo -628.7 15.9

p_loo 48.4 1.7

looic 1257.5 31.7

All Pareto k estimates are good (k < 0.7).

Estimate Est.Error Q2.5 Q97.5

mulow_typeabstract 0.3292445 1.222929 0.22175972 0.4830476

mulow_typeFT 1.6826314 1.242523 1.10380938 2.5915296

mulow_domain2 0.3541763 1.556040 0.14771419 0.8173299

mulow_abstract_year>2008 1.3675341 1.333172 0.78112834 2.3956289

mulow_typeFT:domain2 1.8325636 1.676825 0.66994803 5.0439460

mulow_domain2:abstract_year>2008 1.7145005 1.649923 0.64830786 4.5372994

mulow_typeFT:abstract_year>2008 1.1637176 1.414280 0.58839312 2.3071650

muhigh_typeabstract 0.1173064 1.309958 0.06799565 0.1938844

muhigh_typeFT 1.6164288 1.241929 1.05650932 2.4899132

muhigh_domain2 0.7670616 1.682670 0.27075038 2.0153690

muhigh_abstract_year>2008 0.9610445 1.473615 0.44299593 2.0512891

muhigh_typeFT:domain2 5.1649604 1.826201 1.62960300 16.9566350

muhigh_domain2:abstract_year>2008 0.1609287 1.897129 0.04422284 0.5404283

muhigh_typeFT:abstract_year>2008 0.3845793 1.573274 0.15788813 0.9421003

**Domain 3**

Estimate SE

elpd_waic -617.8 16.1

p_waic 50.1 1.8

waic 1235.6 32.2

1 (0.1%) p_waic estimates greater than 0.4.

LOO Estimate SE

elpd_loo -618.1 16.1

p_loo 50.4 1.8

looic 1236.2 32.2

All Pareto k estimates are good (k < 0.7).

Estimate Est.Error Q2.5 Q97.5

mulow_typeabstract 0.3032502 1.229369 0.20129106 0.4521063

mulow_typeFT 1.5939684 1.242566 1.04173818 2.4474677

mulow_domain3 0.7986395 1.501527 0.35230014 1.7233073

mulow_abstract_year>2008 1.3677517 1.336084 0.77485140 2.4102927

mulow_typeFT:domain3 2.0383375 1.770694 0.68935815 6.5300635

mulow_domain3:abstract_year>2008 1.4629141 1.662395 0.53535620 3.9064273

mulow_typeFT:abstract_year>2008 1.2026071 1.420592 0.60432932 2.4089306

muhigh_typeabstract 0.1011554 1.321114 0.05746663 0.1716625

muhigh_typeFT 1.5168431 1.248587 0.97602167 2.3450412

muhigh_domain3 1.6746923 1.560207 0.68772795 3.9615257

muhigh_abstract_year>2008 0.6461646 1.506470 0.28298306 1.4009241

muhigh_typeFT:domain3 5.2520726 1.785790 1.72983913 16.8612911

muhigh_domain3:abstract_year>2008 3.2958262 1.716787 1.15181656 9.4726864

muhigh_typeFT:abstract_year>2008 0.3151363 1.603873 0.12620727 0.8056606

**Domain 4**

Estimate SE

elpd_waic -641.2 16.0

p_waic 48.5 1.7

waic 1282.4 32.0

2 (0.3%) p_waic estimates greater than 0.4.

LOO Estimate SE

elpd_loo -641.5 16.0

p_loo 48.8 1.7

looic 1282.9 32.0

All Pareto k estimates are good (k < 0.7).

Estimate Est.Error Q2.5 Q97.5

mulow_typeabstract 0.3288078 1.223403 0.21990653 0.4827402

mulow_typeFT 1.7960110 1.250139 1.16441620 2.8087165

mulow_domain4 0.3675092 1.547223 0.15020948 0.8442680

mulow_abstract_year>2008 1.3608060 1.333043 0.77009672 2.3767382

mulow_typeFT:domain4 1.1696054 1.643107 0.44937858 3.1578585

mulow_domain4:abstract_year>2008 1.6861500 1.617448 0.64946490 4.3936268

mulow_typeFT:abstract_year>2008 1.1656859 1.418077 0.58505697 2.3330416

muhigh_typeabstract 0.1195106 1.306722 0.06928781 0.1970086

muhigh_typeFT 1.9377027 1.238071 1.28465763 2.9638410

muhigh_domain4 0.6854942 1.644197 0.25155588 1.7351430

muhigh_abstract_year>2008 0.8385853 1.469222 0.39089533 1.7827134

muhigh_typeFT:domain4 1.5650976 1.731044 0.54714173 4.6278146

muhigh_domain4:abstract_year>2008 1.1246176 1.730796 0.38108661 3.2987428

muhigh_typeFT:abstract_year>2008 0.3443696 1.567554 0.14312921 0.8303687

**Domain 5**

Estimate SE

elpd_waic -620.4 16.8

p_waic 49.9 2.0

waic 1240.9 33.6

3 (0.4%) p_waic estimates greater than 0.4.

LOO Estimate SE

elpd_loo -620.7 16.8

p_loo 50.1 2.0

looic 1241.4 33.6

All Pareto k estimates are good (k < 0.7).

Estimate Est.Error Q2.5 Q97.5

mulow_typeabstract 0.2263857 1.243855 0.14705407 0.3437275

mulow_typeFT 1.2429918 1.255886 0.79246260 1.9503496

mulow_domain5 4.5357134 1.429128 2.26719130 9.1048691

mulow_abstract_year>2008 1.6002988 1.355222 0.88203435 2.8966511

mulow_typeFT:domain5 1.2754752 1.615055 0.51350524 3.3124654

mulow_domain5:abstract_year>2008 0.4802777 1.568688 0.19753579 1.1412237

mulow_typeFT:abstract_year>2008 1.1943489 1.424623 0.59894074 2.4036111

muhigh_typeabstract 0.1149797 1.298950 0.06752942 0.1887248

muhigh_typeFT 2.0403235 1.231802 1.35906370 3.0699702

muhigh_domain5 0.9443945 1.713139 0.31621820 2.6047612

muhigh_abstract_year>2008 0.8812561 1.465276 0.40711058 1.8361691

muhigh_typeFT:domain5 0.6477250 1.905908 0.18499266 2.3143681

muhigh_domain5:abstract_year>2008 0.5042708 2.052520 0.11538836 1.9690621

muhigh_typeFT:abstract_year>2008 0.3431220 1.562896 0.14449472 0.8271761

**Domain 6**

Estimate SE

elpd_waic -606.2 16.5

p_waic 48.1 2.0

waic 1212.4 33.1

4 (0.6%) p_waic estimates greater than 0.4.

LOO Estimate SE

elpd_loo -606.5 16.6

p_loo 48.3 2.0

looic 1212.9 33.1

All Pareto k estimates are good (k < 0.7).

Estimate Est.Error Q2.5 Q97.5

mulow_typeabstract 0.3538309 1.225811 0.23361528 0.5225605

mulow_typeFT 2.4904140 1.270449 1.56609435 4.0130488

mulow_domain6 0.1025520 1.631926 0.03832727 0.2586659

mulow_abstract_year>2008 1.2474410 1.339365 0.70384713 2.2254689

mulow_typeFT:domain6 1.0835571 1.640786 0.41500304 2.8692544

mulow_domain6:abstract_year>2008 6.4223156 1.633909 2.48712330 17.0589617

mulow_typeFT:abstract_year>2008 0.9461443 1.428369 0.47211210 1.8896191

muhigh_typeabstract 0.1312036 1.302626 0.07641318 0.2154695

muhigh_typeFT 3.1576034 1.263235 2.00300931 5.0317639

muhigh_domain6 0.2329535 1.773378 0.07211315 0.6806322

muhigh_abstract_year>2008 0.7929405 1.467243 0.36661128 1.6737140

muhigh_typeFT:domain6 0.2280134 1.911260 0.06417345 0.8230793

muhigh_domain6:abstract_year>2008 1.5864252 2.048766 0.36960443 6.2357235

muhigh_typeFT:abstract_year>2008 0.2874342 1.574637 0.11913681 0.6970929

**Domain 7**

Estimate SE

elpd_waic -610.3 16.8

p_waic 50.0 1.9

waic 1220.5 33.6

1 (0.1%) p_waic estimates greater than 0.4.

LOO Estimate SE

elpd_loo -610.5 16.8

p_loo 50.3 1.9

looic 1221.1 33.6

All Pareto k estimates are good (k < 0.7).

Estimate Est.Error Q2.5 Q97.5

mulow_typeabstract 0.1993836 1.248142 0.12740449 0.3055224

mulow_typeFT 1.3026960 1.256789 0.83090827 2.0438950

mulow_domain7 9.4606128 1.437785 4.68995610 19.2577212

mulow_abstract_year>2008 1.9059865 1.353787 1.05671871 3.4904204

mulow_typeFT:domain7 0.4321467 1.576667 0.17821709 1.0650671

mulow_domain7:abstract_year>2008 0.1790139 1.564762 0.07329814 0.4323245

mulow_typeFT:abstract_year>2008 1.1485149 1.429516 0.57082788 2.2906645

muhigh_typeabstract 0.1058671 1.305133 0.06134161 0.1753397

muhigh_typeFT 2.0885987 1.234623 1.39639593 3.1950155

muhigh_domain7 1.9265322 1.655394 0.70821029 5.0311150

muhigh_abstract_year>2008 0.7122189 1.482663 0.32310675 1.5070186

muhigh_typeFT:domain7 0.2465575 1.773050 0.08060053 0.7587367

muhigh_domain7:abstract_year>2008 1.9079501 1.745071 0.64598873 5.6823135

muhigh_typeFT:abstract_year>2008 0.3902365 1.571958 0.16239899 0.9445720

# CONSORT statement analysis (interaction term; high reference)

**Domain 1**

Estimate SE

elpd_waic -640.2 15.7

p_waic 45.5 1.4

waic 1280.4 31.5

Estimate Est.Error Q2.5 Q97.5

muunclear_typeabstract 7.5725613 1.274170 4.7738473 12.4408661

muunclear_typeFT 0.5019698 1.240293 0.3249939 0.7653387

muunclear_domain1 1.3612725 1.574079 0.5669314 3.3361449

muunclear_abstract_year>2008 1.1069777 1.421115 0.5600900 2.2337003

muunclear_typeFT:domain1 0.5616537 1.704892 0.1945980 1.5777625

muunclear_domain1:abstract_year>2008 3.1628545 1.822052 1.0012591 10.6867135

muunclear_typeFT:abstract_year>2008 2.3912380 1.526431 1.0468651 5.4016747

mulow_typeabstract 2.2626345 1.311636 1.3258439 3.8827003

mulow_typeFT 0.8833036 1.213316 0.5999292 1.2841858

mulow_domain1 0.5752444 1.656464 0.2157879 1.5553157

mulow_abstract_year>2008 1.7087150 1.459808 0.8281157 3.6202046

mulow_typeFT:domain1 1.2633386 1.714969 0.4337858 3.6849572

mulow_domain1:abstract_year>2008 3.4632972 1.789775 1.1231941 11.0711827

mulow_typeFT:abstract_year>2008 2.4837226 1.516097 1.1046511 5.6812640

**Domain 2**

Estimate SE

elpd_waic -630.6 15.9

p_waic 45.6 1.5

waic 1261.3 31.8

Estimate Est.Error Q2.5 Q97.5

muunclear_typeabstract 7.4526609 1.278112 4.6642148 12.2692062

muunclear_typeFT 0.5694512 1.239568 0.3714233 0.8639120

muunclear_domain2 1.5572287 1.584522 0.6470256 3.9064651

muunclear_abstract_year>2008 1.1021126 1.425598 0.5589481 2.2431808

muunclear_typeFT:domain2 0.1899522 1.724510 0.0651426 0.5452815

muunclear_domain2:abstract_year>2008 3.3172380 1.779968 1.0689522 10.3831100

muunclear_typeFT:abstract_year>2008 2.3326375 1.526557 1.0107831 5.3916162

mulow_typeabstract 2.2906149 1.312653 1.3442526 3.8928392

mulow_typeFT 1.0074630 1.215785 0.6855547 1.4817037

mulow_domain2 0.4575740 1.685225 0.1639975 1.2726920

mulow_abstract_year>2008 1.6719896 1.453891 0.7991207 3.4887369

mulow_typeFT:domain2 0.5672120 1.723941 0.1914337 1.6358413

mulow_domain2:abstract_year>2008 4.8660675 1.757926 1.6488687 15.0849084

mulow_typeFT:abstract_year>2008 2.3943498 1.514772 1.0554195 5.4934107

**Domain 3**

Estimate SE

elpd_waic -619.5 16.0

p_waic 45.5 1.5

waic 1238.9 32.1

1 (0.1%) p_waic estimates greater than 0.4.

Estimate Est.Error Q2.5 Q97.5

muunclear_typeabstract 8.6793086 1.282246 5.38985317 14.2696057

muunclear_typeFT 0.6243776 1.237909 0.40804246 0.9402639

muunclear_domain3 0.6376645 1.517488 0.28263390 1.4595744

muunclear_abstract_year>2008 1.5208515 1.450595 0.73997271 3.1684423

muunclear_typeFT:domain3 0.1651346 1.857557 0.04637907 0.5325078

muunclear_domain3:abstract_year>2008 0.3100411 1.730726 0.10588870 0.9064234

muunclear_typeFT:abstract_year>2008 2.5825733 1.563621 1.06546837 6.2173137

mulow_typeabstract 2.4564936 1.323115 1.43511393 4.2841191

mulow_typeFT 1.0430105 1.218552 0.70757390 1.5331571

mulow_domain3 0.4303295 1.593147 0.16922189 1.0553749

mulow_abstract_year>2008 2.1965038 1.487952 1.01631139 4.8163271

mulow_typeFT:domain3 0.5316910 1.655965 0.19721174 1.4339017

mulow_domain3:abstract_year>2008 0.5535409 1.654759 0.20644186 1.4928199

mulow_typeFT:abstract_year>2008 2.8495924 1.547922 1.20817416 6.6331019

**Domain 4**

Estimate SE

elpd_waic -641.5 16.1

p_waic 46.3 1.5

waic 1283.0 32.2

Estimate Est.Error Q2.5 Q97.5

muunclear_typeabstract 7.4116898 1.278648 4.6521150 12.126006

muunclear_typeFT 0.4832556 1.243184 0.3136440 0.736069

muunclear_domain4 1.7629494 1.562753 0.7547626 4.309762

muunclear_abstract_year>2008 1.2584209 1.424373 0.6402752 2.551912

muunclear_typeFT:domain4 0.5903761 1.656614 0.2150779 1.556834

muunclear_domain4:abstract_year>2008 0.7245636 1.709149 0.2539379 2.099172

muunclear_typeFT:abstract_year>2008 2.5093494 1.525890 1.0976272 5.710142

mulow_typeabstract 2.2792222 1.309698 1.3508173 3.883093

mulow_typeFT 0.9416490 1.212442 0.6430404 1.366190

mulow_domain4 0.5173042 1.658880 0.1903631 1.400749

mulow_abstract_year>2008 1.8311913 1.453096 0.8849026 3.804373

mulow_typeFT:domain4 0.8142742 1.702226 0.2888365 2.323400

mulow_domain4:abstract_year>2008 1.4653211 1.715837 0.5145364 4.301312

mulow_typeFT:abstract_year>2008 2.5843238 1.517380 1.1508917 5.813897

**Domain 5**

Estimate SE

elpd_waic -620.6 16.9

p_waic 47.0 1.7

waic 1241.2 33.8

1 (0.1%) p_waic estimates greater than 0.4.

LOO Estimate SE

elpd_loo -620.8 16.9

p_loo 47.2 1.7

looic 1241.6 33.8

All Pareto k estimates are good (k < 0.7).

Estimate Est.Error Q2.5 Q97.5

muunclear_typeabstract 8.3295289 1.273813 5.2421054 13.5659272

muunclear_typeFT 0.4862695 1.230100 0.3216327 0.7234745

muunclear_domain5 0.7119170 1.614413 0.2861271 1.8433307

muunclear_abstract_year>2008 1.1051467 1.416880 0.5608751 2.1981911

muunclear_typeFT:domain5 1.5056131 1.801110 0.4722272 4.7454768

muunclear_domain5:abstract_year>2008 2.5840661 1.858172 0.7828013 8.8623119

muunclear_typeFT:abstract_year>2008 2.5305137 1.516415 1.1009801 5.6209462

mulow_typeabstract 1.6248768 1.330405 0.9389569 2.8438695

mulow_typeFT 0.6397334 1.223250 0.4271868 0.9494529

mulow_domain5 4.3078692 1.596557 1.7534134 11.2479393

mulow_abstract_year>2008 2.0547531 1.470937 0.9514500 4.3985460

mulow_typeFT:domain5 1.7613867 1.708843 0.6205374 5.0990767

mulow_domain5:abstract_year>2008 0.9035115 1.820559 0.2871976 2.9767224

mulow_typeFT:abstract_year>2008 2.5953676 1.520409 1.1555133 5.9688538

**Domain 6**

Estimate SE

elpd_waic -603.4 16.7

p_waic 45.6 1.7

waic 1206.8 33.5

4 (0.6%) p_waic estimates greater than 0.4.

LOO Estimate SE

elpd_loo -603.6 16.7

p_loo 45.8 1.7

looic 1207.2 33.5

All Pareto k estimates are good (k < 0.7)

Estimate Est.Error Q2.5 Q97.5

muunclear_typeabstract 6.6464819 1.274756 4.21417535 10.8834365

muunclear_typeFT 0.2741001 1.272127 0.16997818 0.4339754

muunclear_domain6 9.4010269 1.741126 3.21846531 28.5378938

muunclear_abstract_year>2008 1.3423501 1.430848 0.66673951 2.7388123

muunclear_typeFT:domain6 2.3039575 1.788756 0.72667411 7.0093570

muunclear_domain6:abstract_year>2008 0.2577626 1.856903 0.07868192 0.8977753

muunclear_typeFT:abstract_year>2008 3.2410319 1.533666 1.39024167 7.4513969

mulow_typeabstract 2.2200495 1.304881 1.32880926 3.7337529

mulow_typeFT 0.8480706 1.209933 0.58385213 1.2254495

mulow_domain6 0.4364102 1.832792 0.13276264 1.4086756

mulow_abstract_year>2008 1.7622147 1.458995 0.84879621 3.7114000

mulow_typeFT:domain6 3.0040350 1.828870 0.93628518 9.8844577

mulow_domain6:abstract_year>2008 3.6864147 1.888104 1.08111827 13.2551268

mulow_typeFT:abstract_year>2008 2.3962819 1.515068 1.06170827 5.4366828

**Domain 7**

Estimate SE

elpd_waic -609.1 17.1

p_waic 47.9 1.8

waic 1218.2 34.2

Estimate Est.Error Q2.5 Q97.5

muunclear_typeabstract 9.4923768 1.282408 5.86097779 15.6441081

muunclear_typeFT 0.4741334 1.232013 0.31251746 0.7079133

muunclear_domain7 0.2649187 1.576013 0.10776273 0.6447029

muunclear_abstract_year>2008 1.1864811 1.437794 0.58676685 2.4389327

muunclear_typeFT:domain7 5.0362487 1.710380 1.77022609 14.3141356

muunclear_domain7:abstract_year>2008 1.2708000 1.712569 0.45212564 3.7072987

muunclear_typeFT:abstract_year>2008 2.3884820 1.524711 1.03142404 5.3890990

mulow_typeabstract 1.5460619 1.339717 0.86967307 2.7392677

mulow_typeFT 0.6653657 1.227099 0.44234739 0.9929697

mulow_domain7 4.2621763 1.544864 1.82449735 10.2458043

mulow_abstract_year>2008 2.6649686 1.474315 1.23886710 5.6845688

mulow_typeFT:domain7 1.3655197 1.640646 0.51347745 3.6113978

mulow_domain7:abstract_year>2008 0.1589176 1.669947 0.05813126 0.4357271

mulow_typeFT:abstract_year>2008 2.3761654 1.523220 1.03113401 5.4310236

# CONSORT statement analysis (interaction term; low reference)

**Domain 1**

Estimate SE

elpd_waic -638.4 15.8

p_waic 49.1 1.8

waic 1276.8 31.6

2 (0.3%) p_waic estimates greater than 0.4.

LOO Estimate SE

elpd_loo -638.6 15.8

p_loo 49.4 1.8

looic 1277.3 31.6

All Pareto k estimates are good (k < 0.7).

Estimate Est.Error Q2.5 Q97.5

muhigh_typeabstract 0.3353193 1.346870 0.18528171 0.5923982

muhigh_typeFT 1.1106803 1.218436 0.75999760 1.6344773

muhigh_domain1 1.4029336 1.710685 0.47507252 3.9647991

muhigh_abstract_year>2008 0.6817486 1.500790 0.30543544 1.5040027

muhigh_typeFT:domain1 1.0470567 1.785017 0.33557968 3.3027420

muhigh_domain1:abstract_year>2008 0.1860928 1.989780 0.04532522 0.6849020

muhigh_typeFT:abstract_year>2008 0.3243982 1.562460 0.13565845 0.7731225

muunclear_typeabstract 3.2622210 1.226540 2.20202470 4.9117688

muunclear_typeFT 0.5483318 1.249420 0.35438261 0.8448118

muunclear_domain1 1.7992882 1.497139 0.82806029 3.9972130

muunclear_abstract_year>2008 0.6397884 1.334864 0.36282122 1.1274788

muunclear_typeFT:domain1 0.5182130 1.625983 0.19678916 1.3141643

muunclear_domain1:abstract_year>2008 1.2255242 1.617385 0.48484255 3.1571579

muunclear_typeFT:abstract_year>2008 1.0108878 1.425521 0.50321103 2.0084660

**Domain 2**

Estimate SE

elpd_waic -627.3 15.9

p_waic 49.7 1.8

waic 1254.7 31.9

2 (0.3%) p_waic estimates greater than 0.4.

LOO Estimate SE

elpd_loo -627.6 16.0

p_loo 49.9 1.8

looic 1255.1 31.9

All Pareto k estimates are good (k < 0.7).

Estimate Est.Error Q2.5 Q97.5

muhigh_typeabstract 0.3341876 1.345685 0.18263288 0.5886277

muhigh_typeFT 0.9477192 1.224228 0.63211547 1.4018355

muhigh_domain2 1.2594792 1.758964 0.40797778 3.7971151

muhigh_abstract_year>2008 0.6869316 1.505970 0.30733919 1.5234841

muhigh_typeFT:domain2 3.7295321 1.832441 1.15056589 12.5905531

muhigh_domain2:abstract_year>2008 0.1369168 1.878044 0.03763018 0.4522992

muhigh_typeFT:abstract_year>2008 0.3484092 1.564929 0.14396935 0.8340350

muunclear_typeabstract 3.1446707 1.225248 2.11892629 4.7065037

muunclear_typeFT 0.5507220 1.252596 0.35126522 0.8486969

muunclear_domain2 2.3455945 1.526181 1.04171404 5.4941135

muunclear_abstract_year>2008 0.6488375 1.330451 0.37043180 1.1393286

muunclear_typeFT:domain2 0.3369527 1.668381 0.12033064 0.9060213

muunclear_domain2:abstract_year>2008 1.0938048 1.676910 0.39426077 3.0187379

muunclear_typeFT:abstract_year>2008 1.0273980 1.417938 0.51985676 2.0352944

**Domain 3**

Estimate SE

elpd_waic -616.7 16.4

p_waic 51.2 2.0

waic 1233.4 32.9

5 (0.7%) p_waic estimates greater than 0.4.

LOO Estimate SE

elpd_loo -617.0 16.4

p_loo 51.5 2.0

looic 1234.0 32.9

All Pareto k estimates are good (k < 0.7).

Estimate Est.Error Q2.5 Q97.5

muhigh_typeabstract 0.3075984 1.366749 0.16306503 0.5589160

muhigh_typeFT 0.9183299 1.234449 0.60492925 1.3864934

muhigh_domain3 1.7984435 1.636729 0.67114068 4.6847690

muhigh_abstract_year>2008 0.4792773 1.540877 0.20354206 1.1100339

muhigh_typeFT:domain3 2.7928508 1.694385 0.99076686 7.7664223

muhigh_domain3:abstract_year>2008 2.3078875 1.682317 0.82417325 6.3798325

muhigh_typeFT:abstract_year>2008 0.2720638 1.587072 0.11132782 0.6714350

muunclear_typeabstract 3.4054363 1.225711 2.30283593 5.1407571

muunclear_typeFT 0.5710936 1.245391 0.36891039 0.8672451

muunclear_domain3 1.3011399 1.501862 0.58943104 2.9160745

muunclear_abstract_year>2008 0.7002139 1.334130 0.39673676 1.2368124

muunclear_typeFT:domain3 0.2789241 1.914060 0.07492319 0.9510279

muunclear_domain3:abstract_year>2008 0.5496849 1.726890 0.18651134 1.5796210

muunclear_typeFT:abstract_year>2008 0.9825111 1.422758 0.49261181 1.9582423

**Domain 4**

Estimate SE

elpd_waic -641.1 16.2

p_waic 50.0 1.9

waic 1282.1 32.3

3 (0.4%) p_waic estimates greater than 0.4.

LOO Estimate SE

elpd_loo -641.3 16.2

p_loo 50.2 1.9

looic 1282.6 32.3

All Pareto k estimates are good (k < 0.7).

Estimate Est.Error Q2.5 Q97.5

muhigh_typeabstract 0.3401610 1.338861 0.1881453 0.5906559

muhigh_typeFT 1.0641001 1.217391 0.7239932 1.5524182

muhigh_domain4 1.3295521 1.729019 0.4435609 3.7752832

muhigh_abstract_year>2008 0.6155934 1.506465 0.2712574 1.3666345

muhigh_typeFT:domain4 1.6170016 1.769930 0.5323875 5.0064212

muhigh_domain4:abstract_year>2008 0.8542340 1.744763 0.2764988 2.4821468

muhigh_typeFT:abstract_year>2008 0.3034155 1.568224 0.1258533 0.7250929

muunclear_typeabstract 3.1282410 1.220683 2.1254521 4.6520815

muunclear_typeFT 0.4949413 1.254431 0.3141241 0.7638448

muunclear_domain4 2.6968925 1.519523 1.2002701 6.3095945

muunclear_abstract_year>2008 0.6923356 1.329355 0.3968133 1.2216243

muunclear_typeFT:domain4 0.7830033 1.628468 0.2969028 1.9969958

muunclear_domain4:abstract_year>2008 0.5976896 1.623929 0.2321892 1.5471608

muunclear_typeFT:abstract_year>2008 1.0243446 1.414241 0.5208918 2.0043145

**Domain 5**

Estimate SE

elpd_waic -620.7 16.8

p_waic 50.7 2.0

waic 1241.5 33.6

3 (0.4%) p_waic estimates greater than 0.4.

Estimate SE

elpd_loo -621.0 16.8

p_loo 50.9 2.0

looic 1241.9 33.6

All Pareto k estimates are good (k < 0.7).

Estimate Est.Error Q2.5 Q97.5

muhigh_typeabstract 0.4166629 1.348243 0.2290119 0.7344667

muhigh_typeFT 1.5154178 1.224281 1.0191533 2.2623611

muhigh_domain5 0.3478209 1.671503 0.1230418 0.9165615

muhigh_abstract_year>2008 0.6100500 1.506631 0.2728401 1.3519479

muhigh_typeFT:domain5 0.4104190 1.822341 0.1268848 1.3225364

muhigh_domain5:abstract_year>2008 0.6323410 2.051373 0.1448917 2.4096641

muhigh_typeFT:abstract_year>2008 0.2896823 1.561789 0.1226734 0.6951461

muunclear_typeabstract 4.5185573 1.233718 3.0116900 6.8932486

muunclear_typeFT 0.6839447 1.260737 0.4331799 1.0783828

muunclear_domain5 0.2118568 1.423375 0.1042681 0.4211158

muunclear_abstract_year>2008 0.5816493 1.346342 0.3249630 1.0446586

muunclear_typeFT:domain5 0.8929864 1.629345 0.3363218 2.3115349

muunclear_domain5:abstract_year>2008 2.2769305 1.588621 0.9284750 5.6123880

muunclear_typeFT:abstract_year>2008 1.0258315 1.430918 0.5059091 2.0785849

**Domain 6**

Estimate SE

elpd_waic -604.0 17.1

p_waic 50.3 2.2

waic 1208.0 34.2

6 (0.8%) p_waic estimates greater than 0.4.

Estimate SE

elpd_loo -604.2 17.1

p_loo 50.5 2.3

looic 1208.5 34.3

All Pareto k estimates are good (k < 0.7).

Estimate Est.Error Q2.5 Q97.5

muhigh_typeabstract 0.3435513 1.341335 0.19046966 0.6066180

muhigh_typeFT 1.2546650 1.214868 0.85681924 1.8374665

muhigh_domain6 0.7260378 1.930130 0.19286721 2.5311138

muhigh_abstract_year>2008 0.6424579 1.501597 0.28736104 1.4106387

muhigh_typeFT:domain6 0.4127150 1.997532 0.10531752 1.6162756

muhigh_domain6:abstract_year>2008 0.8287235 1.990685 0.21054589 3.0848721

muhigh_typeFT:abstract_year>2008 0.3021753 1.571539 0.12419042 0.7310159

muunclear_typeabstract 2.9135761 1.230761 1.96629078 4.4092441

muunclear_typeFT 0.3163232 1.284091 0.19257598 0.5130683

muunclear_domain6 10.0758420 1.636608 3.96644547 27.2407527

muunclear_abstract_year>2008 0.7669230 1.348396 0.42753431 1.3733545

muunclear_typeFT:domain6 1.1485105 1.643306 0.42249269 3.0222944

muunclear_domain6:abstract_year>2008 0.1355232 1.632567 0.05118348 0.3475573

muunclear_typeFT:abstract_year>2008 1.3525509 1.438803 0.66241397 2.7295914

**Domain 7**

Estimate SE

elpd_waic -610.2 16.8

p_waic 51.2 2.0

waic 1220.5 33.6

5 (0.7%) p_waic estimates greater than 0.4.

LOO Estimate SE

elpd_loo -610.5 16.8

p_loo 51.5 2.1

looic 1221.0 33.6

All Pareto k estimates are good (k < 0.7).

Estimate Est.Error Q2.5 Q97.5

muhigh_typeabstract 0.3967036 1.359476 0.21546408 0.7158988

muhigh_typeFT 1.4725624 1.221605 0.98852379 2.1675392

muhigh_domain7 0.4893776 1.577131 0.19634348 1.1748861

muhigh_abstract_year>2008 0.4602961 1.520322 0.19987296 1.0275673

muhigh_typeFT:domain7 0.3438167 1.696191 0.11917739 0.9686601

muhigh_domain7:abstract_year>2008 4.3383219 1.718872 1.52163772 12.4990481

muhigh_typeFT:abstract_year>2008 0.3325417 1.567716 0.13905601 0.8120683

muunclear_typeabstract 5.0874228 1.238447 3.35674105 7.7876343

muunclear_typeFT 0.6364046 1.258366 0.40157965 0.9941724

muunclear_domain7 0.1013869 1.440748 0.04901788 0.2057851

muunclear_abstract_year>2008 0.5231638 1.354349 0.28799417 0.9464267

muunclear_typeFT:domain7 3.2916535 1.593426 1.31261552 8.0589526

muunclear_domain7:abstract_year>2008 4.3623672 1.559990 1.81190242 10.4546157

muunclear_typeFT:abstract_year>2008 1.0131428 1.429689 0.50732614 2.0543677

# Sensitivity analysis (domain 2 decisions)

Estimate SE

elpd_waic -640.9 15.2

p_waic 53.1 1.8

waic 1281.8 30.5

1 (0.1%) p_waic estimates greater than 0.4.

Estimate SE

elpd_loo -641.2 15.3

p_loo 53.4 1.8

looic 1282.4 30.5

All Pareto k estimates are good (k < 0.7).

Estimate Est.Error Q2.5 Q97.5

muhigh_typeabstract 0.1124362 1.270004 0.06909686 0.1752134

muhigh_typeFT 1.0787037 1.215999 0.72954498 1.5758635

muhigh_domain2 0.3222555 1.705564 0.10890780 0.8745703

muhigh_typeFT:domain2 0.9654033 1.855544 0.29062346 3.2981832

mulow_typeabstract 0.3779290 1.175502 0.27469416 0.5166664

mulow_typeFT 2.0585749 1.182814 1.47982430 2.8718968

mulow_domain2 0.4508532 1.441552 0.21846541 0.9027862

mulow_typeFT:domain2 1.7369697 1.582561 0.70764496 4.2749974

# Sensitivity analysis (unclear reference)

## Normal (0, 2.5)

**Domain 1**

Estimate Est.Error Q2.5 Q97.5

muhigh_typeabstract 0.09678212 1.292781 0.05749076 0.1565920

muhigh_typeFT 1.26192857 1.218055 0.84460735 1.8384526

muhigh_domain1 0.57421331 1.879689 0.14911097 1.7824236

muhigh_typeFT:domain1 1.83478633 2.108217 0.45375949 8.4317701

mulow_typeabstract 0.36614844 1.185992 0.25933448 0.5088748

mulow_typeFT 2.10029559 1.193290 1.48020127 2.9807565

mulow_domain1 0.41085020 1.541451 0.16704402 0.9247878

mulow_typeFT:domain1 2.56451183 1.776256 0.85678173 8.0962521

**Domain 2**

Estimate Est.Error Q2.5 Q97.5

muhigh_typeabstract 0.1029746 1.289583 0.06122662 0.1648850

muhigh_typeFT 1.0703675 1.223190 0.71846870 1.5803958

muhigh_domain2 0.2279039 2.265376 0.03933941 0.9494093

muhigh_typeFT:domain2 13.9894402 2.499912 2.63958261 94.2073772

mulow_typeabstract 0.3723024 1.185714 0.26482531 0.5136187

mulow_typeFT 2.0839846 1.191822 1.47971840 2.9479991

mulow_domain2 0.3733597 1.543744 0.15202723 0.8360260

mulow_typeFT:domain2 3.4120305 1.859851 1.04049052 11.8947531

**Domain 3**

Estimate Est.Error Q2.5 Q97.5

muhigh_typeabstract 0.07180083 1.327009 0.04036755 0.1216876

muhigh_typeFT 0.85202946 1.237699 0.55181934 1.2726849

muhigh_domain3 2.44489957 1.618164 0.92929522 6.0278279

muhigh_typeFT:domain3 13.57172515 2.474189 2.60415508 90.5425188

mulow_typeabstract 0.33522170 1.186121 0.23682476 0.4640446

mulow_typeFT 2.00688789 1.191506 1.42875270 2.8411419

mulow_domain3 0.83911556 1.475680 0.38099897 1.7636914

mulow_typeFT:domain3 5.64368020 2.439389 1.13049943 37.1472514

**Domain 4**

Estimate Est.Error Q2.5 Q97.5

muhigh_typeabstract 0.07180083 1.327009 0.04036755 0.1216876

muhigh_typeFT 0.85202946 1.237699 0.55181934 1.2726849

muhigh_domain3 2.44489957 1.618164 0.92929522 6.0278279

muhigh_typeFT:domain3 13.57172515 2.474189 2.60415508 90.5425188

mulow_typeabstract 0.33522170 1.186121 0.23682476 0.4640446

mulow_typeFT 2.00688789 1.191506 1.42875270 2.8411419

mulow_domain3 0.83911556 1.475680 0.38099897 1.7636914

mulow_typeFT:domain3 5.64368020 2.439389 1.13049943 37.1472514

**Domain 5**

Estimate Est.Error Q2.5 Q97.5

muhigh_typeabstract 0.0892164 1.294023 0.05216056 0.1434595

muhigh_typeFT 1.3395159 1.210821 0.91578385 1.9347316

muhigh_domain5 0.9317061 1.945846 0.22557227 3.1077122

muhigh_typeFT:domain5 0.4831737 2.424189 0.08543162 2.8357641

mulow_typeabstract 0.2558644 1.203443 0.17641035 0.3644228

mulow_typeFT 1.7283715 1.201237 1.20191155 2.4773139

mulow_domain5 3.9675864 1.395908 2.07130987 7.6673136

mulow_typeFT:domain5 1.0246013 1.739236 0.35815485 3.1251207

**Domain 6**

Estimate Est.Error Q2.5 Q97.5

muhigh_typeabstract 0.1004975 1.297651 0.05852907 0.1621959

muhigh_typeFT 1.9086389 1.239964 1.24283341 2.9038405

muhigh_domain6 0.2632741 2.112291 0.05152693 0.9800574

muhigh_typeFT:domain6 0.1629925 2.569126 0.02687981 1.0903374

mulow_typeabstract 0.3900787 1.186317 0.27627373 0.5420498

mulow_typeFT 2.8868095 1.214101 1.98311901 4.2473709

mulow_domain6 0.1908503 1.700095 0.06087229 0.5010232

mulow_typeFT:domain6 1.2746779 1.867872 0.39488989 4.6111259

**Domain 7**

Estimate Est.Error Q2.5 Q97.5

muhigh_typeabstract 0.07015422 1.320887 0.03902969 0.1176050

muhigh_typeFT 1.41225261 1.215184 0.94919649 2.0529162

muhigh_domain7 4.96627251 1.653635 1.81746531 13.0150827

muhigh_typeFT:domain7 0.07187907 2.009801 0.01805560 0.2770714

mulow_typeabstract 0.23689629 1.205536 0.16220775 0.3394456

mulow_typeFT 2.00644362 1.201476 1.39642616 2.9032889

mulow_domain7 6.92541993 1.420369 3.49321833 13.8633670

mulow_typeFT:domain7 0.20531128 1.656584 0.07697106 0.5551611

## Cauchy(0, 1)

**Domain 1**

Estimate Est.Error Q2.5 Q97.5

muhigh_typeabstract 0.09641062 1.286958 0.05785271 0.1543725

muhigh_typeFT 1.25507996 1.206526 0.86235359 1.8081716

muhigh_domain1 0.73608482 1.612122 0.26261015 1.7311772

muhigh_typeFT:domain1 1.32995050 1.747618 0.47710419 4.4189780

mulow_typeabstract 0.36435380 1.183657 0.26029474 0.5042110

mulow_typeFT 2.08627939 1.191618 1.48300317 2.9597508

mulow_domain1 0.51196794 1.472310 0.22519183 1.0324169

mulow_typeFT:domain1 1.88685738 1.668640 0.73928887 5.5689342

**Domain 2**

Estimate Est.Error Q2.5 Q97.5

muhigh_typeabstract 0.1008148 1.295188 0.05925790 0.1635236

muhigh_typeFT 1.0777363 1.213243 0.73013999 1.5586277

muhigh_domain2 0.4000961 2.146224 0.06476795 1.3036360

muhigh_typeFT:domain2 6.7669282 2.467843 1.55185312 53.8654876

mulow_typeabstract 0.3693105 1.187033 0.26088177 0.5101696

mulow_typeFT 2.0830011 1.193649 1.47031353 2.9748049

mulow_domain2 0.4758437 1.481832 0.20587791 0.9751869

mulow_typeFT:domain2 2.2576995 1.757230 0.83544895 7.6540856

**Domain 3**

Estimate Est.Error Q2.5 Q97.5

muhigh_typeabstract 0.07488655 1.330341 0.04117889 0.1270512

muhigh_typeFT 0.86442269 1.225521 0.57246186 1.2771656

muhigh_domain3 2.25276560 1.620631 0.88045295 5.8959211

muhigh_typeFT:domain3 9.35437878 2.574892 1.81757390 70.5097683

mulow_typeabstract 0.33884799 1.186762 0.23986383 0.4709010

mulow_typeFT 2.00739690 1.187518 1.43638606 2.8329368

mulow_domain3 0.89414189 1.425407 0.42938251 1.7543586

mulow_typeFT:domain3 3.34191810 2.366981 0.83071897 24.1681406

**Domain 4**

Estimate Est.Error Q2.5 Q97.5

muhigh_typeabstract 0.09554686 1.291334 0.05675733 0.1538181

muhigh_typeFT 1.22518930 1.214168 0.83398063 1.7872166

muhigh_domain4 0.76711880 1.600391 0.28410832 1.8089053

muhigh_typeFT:domain4 1.47769190 1.739337 0.53469586 4.8143749

mulow_typeabstract 0.36636904 1.186284 0.25998670 0.5096743

mulow_typeFT 2.24327625 1.195900 1.57364653 3.2039847

mulow_domain4 0.48079688 1.464102 0.21475982 0.9720042

mulow_typeFT:domain4 1.18546524 1.624394 0.47543876 3.3264498

**Domain 5**

Estimate Est.Error Q2.5 Q97.5

muhigh_typeabstract 0.09240706 1.294695 0.05437875 0.1483557

muhigh_typeFT 1.30777966 1.205278 0.90075205 1.8858342

muhigh_domain5 0.85157892 1.675865 0.28948821 2.2454041

muhigh_typeFT:domain5 0.60614414 1.948967 0.15043731 2.0945152

mulow_typeabstract 0.26425137 1.203220 0.18230886 0.3759557

mulow_typeFT 1.69019678 1.196076 1.19105113 2.4148487

mulow_domain5 3.62416031 1.380016 1.92729561 6.8794310

mulow_typeFT:domain5 1.16183068 1.599870 0.47432104 2.9827125

**Domain 6**

Estimate Est.Error Q2.5 Q97.5

muhigh_typeabstract 0.1011504 1.298935 0.05925052 0.1645952

muhigh_typeFT 1.8047379 1.229463 1.19958174 2.6924507

muhigh_domain6 0.2813257 2.190869 0.04991549 1.0400597

muhigh_typeFT:domain6 0.1881936 2.703290 0.02604628 1.2102625

mulow_typeabstract 0.3903428 1.184079 0.27801850 0.5393597

mulow_typeFT 2.7573509 1.209344 1.90878068 4.0363556

mulow_domain6 0.2317006 1.608726 0.08408436 0.5468104

mulow_typeFT:domain6 1.1084435 1.706170 0.40739620 3.4084860

**Domain 7**

Estimate Est.Error Q2.5 Q97.5

muhigh_typeabstract 0.07598519 1.315464 0.04285873 0.1256576

muhigh_typeFT 1.35446700 1.212295 0.92680931 1.9614423

muhigh_domain7 3.42037877 1.721659 1.18531239 9.8524241

muhigh_typeFT:domain7 0.12376642 2.159099 0.02703013 0.5475858

mulow_typeabstract 0.24911949 1.202342 0.17185027 0.3529384

mulow_typeFT 1.91690427 1.198575 1.34188127 2.7323128

mulow_domain7 5.52264992 1.420925 2.82644509 11.1532777

mulow_typeFT:domain7 0.30152569 1.680628 0.10842458 0.8243184

## Cauchy(0, 2.5)

**Domain 1**

Estimate Est.Error Q2.5 Q97.5

muhigh_typeabstract 0.09627961 1.291788 0.05683394 0.1546860

muhigh_typeFT 1.26318361 1.217840 0.84646384 1.8458889

muhigh_domain1 0.60661468 1.831395 0.16826499 1.7877269

muhigh_typeFT:domain1 1.71234898 2.063852 0.45366453 7.7693964

mulow_typeabstract 0.36666842 1.184627 0.26009107 0.5085683

mulow_typeFT 2.11355734 1.196354 1.49884446 3.0221488

mulow_domain1 0.42582681 1.538441 0.17168306 0.9464008

mulow_typeFT:domain1 2.42775546 1.780044 0.81822588 7.8988835

**Domain 2**

Estimate Est.Error Q2.5 Q97.5

muhigh_typeabstract 0.1028573 1.291234 0.06062951 0.1665056

muhigh_typeFT 1.0649986 1.225274 0.70991709 1.5739614

muhigh_domain2 0.2223568 2.483716 0.02749358 0.9718445

muhigh_typeFT:domain2 14.2937038 2.773389 2.47020568 136.0264532

mulow_typeabstract 0.3700306 1.184952 0.26347174 0.5132283

mulow_typeFT 2.0686914 1.190718 1.47161421 2.9150479

mulow_domain2 0.3868296 1.527210 0.16000792 0.8473465

mulow_typeFT:domain2 3.2533741 1.858674 1.02161327 11.8075130

**Domain 3**

Estimate Est.Error Q2.5 Q97.5

muhigh_typeabstract 0.0718720 1.331031 0.04000663 0.1221724

muhigh_typeFT 0.8498139 1.237668 0.55329552 1.2718819

muhigh_domain3 2.3775021 1.632133 0.88833405 6.0760501

muhigh_typeFT:domain3 14.6321358 2.806232 2.53952234 146.6057040

mulow_typeabstract 0.3365055 1.188187 0.23837388 0.4684315

mulow_typeFT 2.0155467 1.188050 1.44261483 2.8296235

mulow_domain3 0.8472258 1.474951 0.38757684 1.7629088

mulow_typeFT:domain3 5.8373516 2.704501 1.08876177 55.3143866

**Domain 4**

Estimate Est.Error Q2.5 Q97.5

muhigh_typeabstract 0.09579982 1.296209 0.05617741 0.1549714

muhigh_typeFT 1.23190543 1.222472 0.82140131 1.8197987

muhigh_domain4 0.61135193 1.832123 0.16728293 1.8384186

muhigh_typeFT:domain4 1.94148082 2.021758 0.52568507 8.3303145

mulow_typeabstract 0.36602579 1.184106 0.26116797 0.5066363

mulow_typeFT 2.26697354 1.198176 1.60166479 3.2545151

mulow_domain4 0.41696244 1.520270 0.17381353 0.9193503

mulow_typeFT:domain4 1.40797733 1.752888 0.48737871 4.3465544

**Domain 5**

Estimate Est.Error Q2.5 Q97.5

muhigh_typeabstract 0.08949885 1.298348 0.05215902 0.1455068

muhigh_typeFT 1.33582732 1.214862 0.90812125 1.9600126

muhigh_domain5 0.92346344 1.877426 0.24727399 2.9192992

muhigh_typeFT:domain5 0.49876623 2.295538 0.09613993 2.5517289

mulow_typeabstract 0.25612579 1.206160 0.17614763 0.3683456

mulow_typeFT 1.72317095 1.202222 1.20171053 2.4690197

mulow_domain5 3.91632448 1.396634 2.04047999 7.5648393

mulow_typeFT:domain5 1.04364174 1.712355 0.37779841 3.0852712

**Domain 6**

Estimate Est.Error Q2.5 Q97.5

muhigh_typeabstract 0.09963234 1.296654 0.05901105 0.1620225

muhigh_typeFT 1.88587535 1.235891 1.23559202 2.8591705

muhigh_domain6 0.26841555 2.173163 0.04914368 1.0259407

muhigh_typeFT:domain6 0.16049186 2.696175 0.02290716 1.1342196

mulow_typeabstract 0.38797365 1.183682 0.27664881 0.5374054

mulow_typeFT 2.86071750 1.212401 1.97209715 4.1997419

mulow_domain6 0.19858284 1.679689 0.06656549 0.5087526

mulow_typeFT:domain6 1.23399573 1.834083 0.38837510 4.2216956

**Domain 7**

Estimate Est.Error Q2.5 Q97.5

muhigh_typeabstract 0.07003826 1.326844 0.03897850 0.1188770

muhigh_typeFT 1.41078500 1.221812 0.94788273 2.0814651

muhigh_domain7 4.89399424 1.677436 1.75503390 13.3741841

muhigh_typeFT:domain7 0.07276718 2.068463 0.01728095 0.2993011

mulow_typeabstract 0.23848899 1.205064 0.16328011 0.3399675

mulow_typeFT 2.00020807 1.203928 1.39558512 2.8985687

mulow_domain7 6.78434959 1.429338 3.43598631 13.9097552

mulow_typeFT:domain7 0.21253998 1.670687 0.07694032 0.5784928

# Sensitivity analysis (high reference)

## Normal (0, 2.5)

**Domain 1**

Estimate Est.Error Q2.5 Q97.5

mulow_typeabstract 3.1473673 1.295199 1.9214679 5.3078730

mulow_typeFT 1.5419438 1.184234 1.1122232 2.1470717

mulow_domain1 0.6956787 1.956849 0.1964857 2.7462276

mulow_typeFT:domain1 1.4415650 2.101509 0.3163528 5.9254803

muunclear_typeabstract 8.8333216 1.258578 5.7058911 14.0277508

muunclear_typeFT 0.6986478 1.196555 0.4866290 0.9903292

muunclear_domain1 1.7911763 1.796666 0.6225265 6.1126539

muunclear_typeFT:domain1 0.5205700 2.029819 0.1230208 1.9820307

**Domain 2**

Estimate Est.Error Q2.5 Q97.5

mulow_typeabstract 3.00304945 1.285190 1.85397443 4.9909191

mulow_typeFT 1.76236172 1.188396 1.25016468 2.4780757

mulow_domain2 1.25562844 2.176310 0.29876402 6.2614058

mulow_typeFT:domain2 0.35520278 2.303983 0.06333986 1.6957619

muunclear_typeabstract 8.24671464 1.251647 5.39845282 12.9377290

muunclear_typeFT 0.81302153 1.198745 0.56763098 1.1465092

muunclear_domain2 3.86996880 2.022755 1.09265191 17.0328073

muunclear_typeFT:domain2 0.08505761 2.276224 0.01586236 0.3954934

**Domain 3**

Estimate Est.Error Q2.5 Q97.5

mulow_typeabstract 3.62384961 1.313426 2.131320290 6.2695266

mulow_typeFT 2.06067290 1.194668 1.463998502 2.9303384

mulow_domain3 0.35591630 1.700319 0.126240990 1.0089476

mulow_typeFT:domain3 0.51217430 1.884342 0.147658489 1.7515626

muunclear_typeabstract 11.12566260 1.275948 7.074439133 18.2890129

muunclear_typeFT 0.98631674 1.198640 0.684057134 1.3922873

muunclear_domain3 0.43855911 1.586599 0.181178693 1.0896206

muunclear_typeFT:domain3 0.06675375 2.708723 0.007979677 0.3987603

**Domain 4**

Estimate Est.Error Q2.5 Q97.5

mulow_typeabstract 3.1438500 1.291737 1.9243639 5.2583966

mulow_typeFT 1.6894471 1.186277 1.2070040 2.3556635

mulow_domain4 0.6729737 1.964640 0.1840887 2.6815070

mulow_typeFT:domain4 0.7710661 2.122994 0.1700035 3.3071265

muunclear_typeabstract 8.8086409 1.256267 5.7255867 13.9417295

muunclear_typeFT 0.7094716 1.195387 0.4954085 0.9987813

muunclear_domain4 1.7627279 1.792138 0.6101466 6.0726697

muunclear_typeFT:domain4 0.4978309 1.991048 0.1227536 1.8145646

**Domain 5**

Estimate Est.Error Q2.5 Q97.5

mulow_typeabstract 2.3504351 1.302229 1.4086020 3.955835

mulow_typeFT 1.2064478 1.188808 0.8583936 1.685599

mulow_domain5 4.0889129 1.863775 1.2887344 14.876583

mulow_typeFT:domain5 1.9513354 2.171101 0.4052851 8.765470

muunclear_typeabstract 9.6304569 1.255440 6.2549341 15.304194

muunclear_typeFT 0.6672142 1.190518 0.4700452 0.935624

muunclear_domain5 0.9631497 1.836037 0.3139883 3.431698

muunclear_typeFT:domain5 1.9929360 2.283746 0.3877185 9.937908

**Domain 6**

Estimate Est.Error Q2.5 Q97.5

mulow_typeabstract 3.1058377 1.282842 1.9176859 5.1314478

mulow_typeFT 1.4066642 1.180238 1.0145337 1.9422545

mulow_domain6 0.8036575 2.294116 0.1663864 4.3665599

mulow_typeFT:domain6 5.6335516 2.607744 0.8279226 37.0754692

muunclear_typeabstract 8.2579209 1.259233 5.3472075 13.1573631

muunclear_typeFT 0.4532203 1.217178 0.3034711 0.6586424

muunclear_domain6 4.4469326 2.076339 1.1960271 20.8632201

muunclear_typeFT:domain6 4.5800919 2.422197 0.7805978 25.5934324

**Domain 7**

Estimate Est.Error Q2.5 Q97.5

mulow_typeabstract 2.7044619 1.322258 1.58654883 4.7330723

mulow_typeFT 1.3262241 1.189997 0.93901373 1.8586373

mulow_domain7 1.5531733 1.608045 0.62964378 4.1065828

mulow_typeFT:domain7 2.3076039 1.868558 0.67435884 7.8701056

muunclear_typeabstract 12.2316722 1.281554 7.66670391 20.3741725

muunclear_typeFT 0.6198093 1.199945 0.43158379 0.8775801

muunclear_domain7 0.1895980 1.619571 0.07480534 0.4945712

muunclear_typeFT:domain7 13.8810624 1.944265 3.69787277 51.5466099

## Cauchy(0, 1)

**Domain 1**

Estimate Est.Error Q2.5 Q97.5

mulow_typeabstract 2.9127678 1.285560 1.8042308 4.8390539

mulow_typeFT 1.5079566 1.183235 1.0882902 2.1139080

mulow_domain1 0.7256743 1.601896 0.2785011 1.8055298

mulow_typeFT:domain1 1.3802634 1.692155 0.5099339 4.0637173

muunclear_typeabstract 8.4720682 1.251906 5.5292107 13.4318533

muunclear_typeFT 0.6983960 1.188617 0.4927467 0.9792547

muunclear_domain1 1.5959246 1.538954 0.7209196 3.9756318

muunclear_typeFT:domain1 0.6167434 1.703759 0.2015279 1.6472591

**Domain 2**

Estimate Est.Error Q2.5 Q97.5

mulow_typeabstract 2.8948488 1.282209 1.78532673 4.7582364

mulow_typeFT 1.6959594 1.188389 1.20420642 2.3768655

mulow_domain2 0.7752484 1.707941 0.28797639 2.4104114

mulow_typeFT:domain2 0.6197713 1.799307 0.17817379 1.8104034

muunclear_typeabstract 8.1944670 1.249597 5.34902013 12.8351922

muunclear_typeFT 0.8018946 1.191288 0.56317404 1.1236927

muunclear_domain2 2.2687991 1.730050 0.88664683 7.5724424

muunclear_typeFT:domain2 0.1613576 2.043619 0.03513152 0.5801091

**Domain 3**

Estimate Est.Error Q2.5 Q97.5

mulow_typeabstract 3.28220807 1.307134 1.969772239 5.6567792

mulow_typeFT 1.96207227 1.193911 1.390058492 2.7845810

mulow_domain3 0.40976807 1.629318 0.155417879 1.0477785

mulow_typeFT:domain3 0.49087842 1.780295 0.152156557 1.4962367

muunclear_typeabstract 10.31655042 1.271289 6.611577877 16.8428744

muunclear_typeFT 0.96564303 1.192202 0.680695028 1.3586724

muunclear_domain3 0.50673542 1.553054 0.207990553 1.1834273

muunclear_typeFT:domain3 0.06778315 3.122465 0.005053496 0.4529419

**Domain 4**

Estimate Est.Error Q2.5 Q97.5

mulow_typeabstract 2.9515047 1.284195 1.8181161 4.8525811

mulow_typeFT 1.6357662 1.180011 1.1848954 2.2746010

mulow_domain4 0.6247540 1.591564 0.2418405 1.5042772

mulow_typeFT:domain4 0.8707103 1.678463 0.3077314 2.3960354

muunclear_typeabstract 8.5729801 1.253319 5.5957968 13.5464175

muunclear_typeFT 0.7061193 1.191850 0.5001060 0.9904637

muunclear_domain4 1.4598445 1.526717 0.6694764 3.5392539

muunclear_typeFT:domain4 0.6394104 1.681135 0.2155153 1.6653885

**Domain 5**

Estimate Est.Error Q2.5 Q97.5

mulow_typeabstract 2.1839206 1.299524 1.3200270 3.6369765

mulow_typeFT 1.1982902 1.186842 0.8572351 1.6706616

mulow_domain5 4.0921875 1.680217 1.5344161 11.6198330

mulow_typeFT:domain5 1.6529478 1.813895 0.5458445 5.7375743

muunclear_typeabstract 9.1321868 1.250289 6.0237412 14.3738284

muunclear_typeFT 0.6843957 1.185472 0.4836399 0.9483573

muunclear_domain5 0.9919005 1.616987 0.3962365 2.6312183

muunclear_typeFT:domain5 1.5686054 1.854014 0.5007838 5.7761517

**Domain 6**

Estimate Est.Error Q2.5 Q97.5

mulow_typeabstract 2.8519082 1.282283 1.7641357 4.6623110

mulow_typeFT 1.4043161 1.177601 1.0198428 1.9388877

mulow_domain6 1.0748192 1.936952 0.2877167 4.0316258

mulow_typeFT:domain6 3.1021962 2.254636 0.7551333 18.0542690

muunclear_typeabstract 7.8022036 1.252854 5.1082537 12.3281096

muunclear_typeFT 0.4740745 1.212712 0.3231533 0.6865067

muunclear_domain6 5.2652113 1.945786 1.5316619 20.2742678

muunclear_typeFT:domain6 2.7947998 2.241444 0.6779791 15.9829237

**Domain 7**

Estimate Est.Error Q2.5 Q97.5

mulow_typeabstract 2.4212241 1.311479 1.44917475 4.1883966

mulow_typeFT 1.3185961 1.183434 0.94673293 1.8356779

mulow_domain7 1.8992952 1.563339 0.82550985 4.6889161

mulow_typeFT:domain7 1.6657019 1.757538 0.57987292 5.4743053

muunclear_typeabstract 11.1423613 1.270397 7.07231450 18.1929241

muunclear_typeFT 0.6389891 1.195363 0.44456430 0.8975414

muunclear_domain7 0.2495379 1.640206 0.09630118 0.6705389

muunclear_typeFT:domain7 9.0645181 1.996401 2.36277372 36.1107002

## Cauchy(0, 2.5)

**Domain 1**

Estimate Est.Error Q2.5 Q97.5

mulow_typeabstract 3.1001031 1.291440 1.9117286 5.1733961

mulow_typeFT 1.5324748 1.186022 1.0963230 2.1414926

mulow_domain1 0.7167446 1.903282 0.2088780 2.6669577

mulow_typeFT:domain1 1.3997952 2.057370 0.3304697 5.5585817

muunclear_typeabstract 8.7394545 1.256584 5.6937724 13.9352428

muunclear_typeFT 0.6995529 1.197466 0.4874685 0.9870557

muunclear_domain1 1.7997680 1.764651 0.6384182 5.9748691

muunclear_typeFT:domain1 0.5220345 1.985045 0.1262030 1.9387942

**Domain 2**

Estimate Est.Error Q2.5 Q97.5

mulow_typeabstract 2.99040317 1.292061 1.83017632 5.0151364

mulow_typeFT 1.74928867 1.190972 1.24567086 2.4702571

mulow_domain2 1.18796418 2.177890 0.29303441 6.3702127

mulow_typeFT:domain2 0.37952174 2.304282 0.06434628 1.7187146

muunclear_typeabstract 8.25635944 1.255680 5.37290410 13.1435877

muunclear_typeFT 0.81237704 1.199715 0.56665443 1.1576069

muunclear_domain2 3.68765442 2.073859 1.06304484 19.2337369

muunclear_typeFT:domain2 0.08874109 2.378141 0.01366111 0.4201856

**Domain 3**

Estimate Est.Error Q2.5 Q97.5

mulow_typeabstract 3.56602764 1.313176 2.112583213 6.1625405

mulow_typeFT 2.04676282 1.193341 1.445936259 2.8924807

mulow_domain3 0.36735943 1.700622 0.131809493 1.0589313

mulow_typeFT:domain3 0.49916543 1.877935 0.142273716 1.6561938

muunclear_typeabstract 10.99950593 1.276281 6.982845170 18.2162101

muunclear_typeFT 0.98502609 1.198145 0.683920499 1.3939448

muunclear_domain3 0.45367987 1.587508 0.186381255 1.1678635

muunclear_typeFT:domain3 0.05650245 3.275150 0.003786951 0.3865301

**Domain 4**

Estimate Est.Error Q2.5 Q97.5

mulow_typeabstract 3.1168540 1.288819 1.9120111 5.162510

mulow_typeFT 1.6796207 1.187576 1.2051922 2.370664

mulow_domain4 0.6636802 1.899186 0.1939999 2.425057

mulow_typeFT:domain4 0.7895837 2.035885 0.1906738 3.057031

muunclear_typeabstract 8.7952144 1.253204 5.7632964 13.973133

muunclear_typeFT 0.7088258 1.197900 0.4954131 1.005141

muunclear_domain4 1.7046551 1.758572 0.6055569 5.618726

muunclear_typeFT:domain4 0.5209829 1.935404 0.1350358 1.770131

**Domain 5**

Estimate Est.Error Q2.5 Q97.5

mulow_typeabstract 2.3323704 1.302889 1.3986294 3.9394019

mulow_typeFT 1.2032171 1.194640 0.8455837 1.7023730

mulow_domain5 4.0365595 1.824010 1.3027736 14.1162394

mulow_typeFT:domain5 1.9258268 2.085580 0.4742914 8.1976852

muunclear_typeabstract 9.5825139 1.253865 6.2582512 15.2340098

muunclear_typeFT 0.6675764 1.188423 0.4752023 0.9312225

muunclear_domain5 0.9560477 1.792160 0.3227276 3.1859978

muunclear_typeFT:domain5 1.9561345 2.211201 0.4194078 9.3720554

**Domain 6**

Estimate Est.Error Q2.5 Q97.5

mulow_typeabstract 3.0659614 1.282182 1.9176801 5.0301375

mulow_typeFT 1.4040739 1.180114 1.0121837 1.9534755

mulow_domain6 0.8333831 2.284501 0.1654605 4.2818276

mulow_typeFT:domain6 5.2545686 2.674044 0.8299771 38.2939619

muunclear_typeabstract 8.1840001 1.256562 5.3213695 12.9705243

muunclear_typeFT 0.4566817 1.216253 0.3079993 0.6655915

muunclear_domain6 4.5515499 2.077101 1.2373655 21.1183183

muunclear_typeFT:domain6 4.3047734 2.468730 0.7301714 25.8416864

**Domain 7**

Estimate Est.Error Q2.5 Q97.5

mulow_typeabstract 2.6670131 1.326010 1.53473549 4.6302884

mulow_typeFT 1.3273999 1.188129 0.94728767 1.8675785

mulow_domain7 1.6081836 1.615280 0.64611820 4.1516921

mulow_typeFT:domain7 2.2072288 1.867481 0.66766056 7.6489779

muunclear_typeabstract 12.0911234 1.283003 7.60328002 20.1979554

muunclear_typeFT 0.6214745 1.200945 0.43074158 0.8837228

muunclear_domain7 0.1964474 1.637773 0.07525497 0.5158284

muunclear_typeFT:domain7 13.3459854 1.986279 3.56158739 51.3337741

# Sensitivity analysis (low reference)

## Normal (0, 2.5)

**Domain 1**

Estimate Est.Error Q2.5 Q97.5

muunclear_typeabstract 2.6590573 1.176495 1.9472814 3.6831015

muunclear_typeFT 0.4436793 1.194675 0.3103283 0.6243490

muunclear_domain1 2.3970197 1.515140 1.0998616 5.5792565

muunclear_typeFT:domain1 0.3932438 1.765880 0.1261057 1.1677322

muhigh_typeabstract 0.2387214 1.314758 0.1379847 0.4018623

muhigh_typeFT 0.5843265 1.207519 0.3985228 0.8359977

muhigh_domain1 1.2218790 2.009102 0.2888868 4.5280796

muhigh_typeFT:domain1 0.8335673 2.147221 0.1938735 3.8521857

**Domain 2**

Estimate Est.Error Q2.5 Q97.5

muunclear_typeabstract 2.6015288 1.176682 1.90563794 3.6182566

muunclear_typeFT 0.4551274 1.190551 0.32144537 0.6363359

muunclear_domain2 2.8397631 1.529776 1.27881950 6.7303543

muunclear_typeFT:domain2 0.2431379 1.867324 0.06994116 0.8049441

muhigh_typeabstract 0.2497933 1.318649 0.14298661 0.4221751

muhigh_typeFT 0.5031340 1.215333 0.33913240 0.7305992

muhigh_domain2 0.4781966 2.487094 0.06708489 2.4357783

muhigh_typeFT:domain2 5.1197802 2.611661 0.89791163 38.8324203

**Domain 3**

Estimate Est.Error Q2.5 Q97.5

muunclear_typeabstract 2.8891518 1.175010 2.10592183 3.9842843

muunclear_typeFT 0.4740345 1.184491 0.33832519 0.6569078

muunclear_domain3 1.1673111 1.470502 0.55687239 2.5287007

muunclear_typeFT:domain3 0.1156958 2.763492 0.01242232 0.6762018

muhigh_typeabstract 0.1907798 1.357561 0.10280218 0.3397795

muhigh_typeFT 0.4110566 1.231700 0.26495273 0.6049901

muhigh_domain3 2.7061611 1.738645 0.88814239 7.9097354

muhigh_typeFT:domain3 2.5493330 1.941297 0.70201366 9.5543858

**Domain 4**

Estimate Est.Error Q2.5 Q97.5

muunclear_typeabstract 2.6574537 1.177394 1.9471907 3.7029181

muunclear_typeFT 0.4146501 1.194988 0.2901518 0.5837901

muunclear_domain4 2.4374194 1.529612 1.0988115 5.8312459

muunclear_typeFT:domain4 0.6638429 1.776591 0.2095861 1.9839059

muhigh_typeabstract 0.2373331 1.320416 0.1343835 0.4018057

muhigh_typeFT 0.5325989 1.211835 0.3598706 0.7672727

muhigh_domain4 1.2869628 2.015748 0.3067827 4.9412387

muhigh_typeFT:domain4 1.5350273 2.177043 0.3434747 7.4399046

**Domain 5**

Estimate Est.Error Q2.5 Q97.5

muunclear_typeabstract 3.7533638 1.194808 2.67585331 5.3914944

muunclear_typeFT 0.5306741 1.198814 0.37011106 0.7562057

muunclear_domain5 0.2584645 1.394542 0.13419856 0.4894013

muunclear_typeFT:domain5 1.0587304 1.741150 0.34462083 3.0189854

muhigh_typeabstract 0.2988347 1.334976 0.16457187 0.5171165

muhigh_typeFT 0.7453233 1.213331 0.50110697 1.0847902

muhigh_domain5 0.2814490 1.925715 0.07117734 0.9261901

muhigh_typeFT:domain5 0.4347001 2.253584 0.09015891 2.2022403

**Domain 6**

Estimate Est.Error Q2.5 Q97.5

muunclear_typeabstract 2.5293148 1.177879 1.84026348 3.5104405

muunclear_typeFT 0.3159754 1.215224 0.21467325 0.4588221

muunclear_domain6 4.9123840 1.657586 1.97030394 14.3998372

muunclear_typeFT:domain6 0.9297035 1.824959 0.27141761 2.8644282

muhigh_typeabstract 0.2368628 1.323099 0.13226172 0.4015669

muhigh_typeFT 0.6539935 1.207892 0.44648975 0.9383772

muhigh_domain6 1.0446950 2.386301 0.16979530 5.1722327

muhigh_typeFT:domain6 0.1642011 2.785943 0.02196319 1.2862841

**Domain 7**

Estimate Est.Error Q2.5 Q97.5

muunclear_typeabstract 4.1376174 1.198800 2.93415985 5.9491818

muunclear_typeFT 0.4515043 1.204022 0.30994945 0.6449161

muunclear_domain7 0.1351396 1.424410 0.06663587 0.2703210

muunclear_typeFT:domain7 5.7709694 1.659565 2.15206000 15.5042527

muhigh_typeabstract 0.2499106 1.360107 0.13341293 0.4496297

muhigh_typeFT 0.6868484 1.210712 0.46753346 0.9936453

muhigh_domain7 0.8880701 1.620567 0.33643258 2.2337013

muhigh_typeFT:domain7 0.2807966 1.911728 0.07824775 1.0070068

## Cauchy(0, 1)

**Domain 1**

Estimate Est.Error Q2.5 Q97.5

muunclear_typeabstract 2.6894253 1.174190 1.9738680 3.7136860

muunclear_typeFT 0.4490967 1.189134 0.3165940 0.6233879

muunclear_domain1 1.9345567 1.443308 0.9845015 4.1520671

muunclear_typeFT:domain1 0.5233029 1.654066 0.1845195 1.3134718

muhigh_typeabstract 0.2559131 1.310729 0.1487366 0.4276894

muhigh_typeFT 0.6077715 1.200921 0.4194918 0.8633084

muhigh_domain1 1.0308446 1.651609 0.3642260 2.7544171

muhigh_typeFT:domain1 0.9935755 1.742324 0.3292492 3.0353502

**Domain 2**

Estimate Est.Error Q2.5 Q97.5

muunclear_typeabstract 2.6155660 1.174995 1.91393759 3.6157313

muunclear_typeFT 0.4621107 1.190238 0.32425186 0.6463448

muunclear_domain2 2.4385316 1.497911 1.16591877 5.6190982

muunclear_typeFT:domain2 0.3067253 1.834732 0.08542338 0.9267893

muhigh_typeabstract 0.2585608 1.311758 0.14964467 0.4292180

muhigh_typeFT 0.5292083 1.208772 0.35939803 0.7561907

muhigh_domain2 0.7379866 1.981500 0.15819015 2.2883848

muhigh_typeFT:domain2 3.1568993 2.136356 0.87369608 16.8099005

**Domain 3**

Estimate Est.Error Q2.5 Q97.5

muunclear_typeabstract 2.9051444 1.177200 2.12460633 4.0150582

muunclear_typeFT 0.4840654 1.182176 0.34757172 0.6698490

muunclear_domain3 1.0759334 1.414750 0.55801909 2.1633853

muunclear_typeFT:domain3 0.1684865 2.873992 0.01484738 0.8808306

muhigh_typeabstract 0.2079675 1.350388 0.11245804 0.3638102

muhigh_typeFT 0.4414831 1.230196 0.29011517 0.6539668

muhigh_domain3 2.4346247 1.649564 0.91456708 6.5116216

muhigh_typeFT:domain3 2.5891836 1.852797 0.82981021 9.1143229

**Domain 4**

Estimate Est.Error Q2.5 Q97.5

muunclear_typeabstract 2.6677391 1.174802 1.9480861 3.6786182

muunclear_typeFT 0.4223900 1.188671 0.2993835 0.5888217

muunclear_domain4 2.0867085 1.431610 1.0659726 4.3495353

muunclear_typeFT:domain4 0.7706719 1.602109 0.2921920 1.8611610

muhigh_typeabstract 0.2517730 1.309094 0.1461810 0.4215401

muhigh_typeFT 0.5608107 1.198183 0.3881050 0.7941882

muhigh_domain4 1.2303994 1.659605 0.4322337 3.2513938

muhigh_typeFT:domain4 1.5016768 1.759569 0.5247314 4.9583504

**Domain 5**

Estimate Est.Error Q2.5 Q97.5

muunclear_typeabstract 3.6687749 1.191737 2.62536125 5.2168291

muunclear_typeFT 0.5394267 1.192267 0.38099449 0.7577631

muunclear_domain5 0.2831421 1.378800 0.14902713 0.5315674

muunclear_typeFT:domain5 0.9820453 1.601925 0.37499696 2.4194961

muhigh_typeabstract 0.3101896 1.325006 0.17508714 0.5304723

muhigh_typeFT 0.7608960 1.200865 0.52630461 1.0805396

muhigh_domain5 0.3080953 1.819525 0.08688697 0.9137765

muhigh_typeFT:domain5 0.4543166 2.031690 0.10600110 1.7136430

**Domain 6**

Estimate Est.Error Q2.5 Q97.5

muunclear_typeabstract 2.5379922 1.178771 1.85339065 3.5373117

muunclear_typeFT 0.3241457 1.214781 0.22023491 0.4712980

muunclear_domain6 4.0139740 1.547809 1.77653937 10.0142167

muunclear_typeFT:domain6 1.1181844 1.645616 0.41103763 2.9756284

muhigh_typeabstract 0.2547966 1.314774 0.14591200 0.4261881

muhigh_typeFT 0.6741109 1.201863 0.46466012 0.9602601

muhigh_domain6 0.7685812 2.009431 0.17827826 2.8954562

muhigh_typeFT:domain6 0.2621178 2.449488 0.03989229 1.2873773

**Domain 7**

Estimate Est.Error Q2.5 Q97.5

muunclear_typeabstract 4.0289250 1.197069 2.86046246 5.8034977

muunclear_typeFT 0.4694743 1.202781 0.32312139 0.6691601

muunclear_domain7 0.1544183 1.429290 0.07501823 0.3083332

muunclear_typeFT:domain7 4.6094595 1.689993 1.67948528 13.0647040

muhigh_typeabstract 0.2686654 1.340605 0.14981764 0.4698635

muhigh_typeFT 0.7068232 1.200936 0.48837328 1.0048403

muhigh_domain7 0.8314126 1.543655 0.35060619 1.9427996

muhigh_typeFT:domain7 0.3295948 1.846811 0.09305033 1.0254561

## Cauchy(0, 2.5)

**Domain 1**

Estimate Est.Error Q2.5 Q97.5

muunclear_typeabstract 2.6743821 1.177968 1.9558837 3.7106540

muunclear_typeFT 0.4460380 1.190900 0.3145871 0.6249386

muunclear_domain1 2.3024332 1.512280 1.0563092 5.3992899

muunclear_typeFT:domain1 0.4174968 1.761176 0.1314309 1.2372900

muhigh_typeabstract 0.2425788 1.313981 0.1382551 0.4071468

muhigh_typeFT 0.5879701 1.208550 0.3988593 0.8431750

muhigh_domain1 1.1580789 1.967970 0.2865620 4.0356778

muhigh_typeFT:domain1 0.8789134 2.108349 0.2138541 4.0759989

**Domain 2**

Estimate Est.Error Q2.5 Q97.5

muunclear_typeabstract 2.6015795 1.176590 1.90076690 3.5928652

muunclear_typeFT 0.4568126 1.191507 0.31992833 0.6379223

muunclear_domain2 2.8098165 1.531651 1.27249376 6.8157578

muunclear_typeFT:domain2 0.2470641 1.879487 0.06775672 0.7966423

muhigh_typeabstract 0.2518527 1.312814 0.14565035 0.4251946

muhigh_typeFT 0.5073044 1.216181 0.34199742 0.7343210

muhigh_domain2 0.5063640 2.556909 0.06284672 2.4217234

muhigh_typeFT:domain2 4.8374735 2.712864 0.90766329 43.8025176

**Domain 3**

Estimate Est.Error Q2.5 Q97.5

muunclear_typeabstract 2.8942928 1.178639 2.120517429 4.0366849

muunclear_typeFT 0.4782444 1.184759 0.339170150 0.6624986

muunclear_domain3 1.1558497 1.466178 0.556036378 2.5196192

muunclear_typeFT:domain3 0.1113512 3.103207 0.008184036 0.6978985

muhigh_typeabstract 0.1926858 1.359704 0.101822519 0.3459386

muhigh_typeFT 0.4141042 1.237939 0.266834235 0.6185310

muhigh_domain3 2.6579488 1.717580 0.908250708 7.5092356

muhigh_typeFT:domain3 2.5607853 1.913786 0.745301598 9.4405829

**Domain 4**

Estimate Est.Error Q2.5 Q97.5

muunclear_typeabstract 2.6509179 1.172808 1.9444397 3.6400717

muunclear_typeFT 0.4147185 1.189787 0.2934194 0.5798535

muunclear_domain4 2.3755357 1.514240 1.0946569 5.4963298

muunclear_typeFT:domain4 0.6845868 1.741580 0.2253952 1.9882690

muhigh_typeabstract 0.2390073 1.314319 0.1363692 0.3998839

muhigh_typeFT 0.5362823 1.209283 0.3635955 0.7674927

muhigh_domain4 1.2766739 1.937023 0.3295649 4.4271463

muhigh_typeFT:domain4 1.5417869 2.082754 0.3832884 6.7524967

**Domain 5**

Estimate Est.Error Q2.5 Q97.5

muunclear_typeabstract 3.7470834 1.192611 2.68630384 5.3806493

muunclear_typeFT 0.5319285 1.197559 0.36964821 0.7490208

muunclear_domain5 0.2613276 1.380775 0.13938040 0.4874024

muunclear_typeFT:domain5 1.0454438 1.710449 0.35799510 2.9119762

muhigh_typeabstract 0.2999105 1.331217 0.16765107 0.5184262

muhigh_typeFT 0.7447300 1.211148 0.50574221 1.0721290

muhigh_domain5 0.2833738 1.897426 0.07293343 0.9179206

muhigh_typeFT:domain5 0.4379498 2.205596 0.09455983 2.0701531

**Domain 6**

Estimate Est.Error Q2.5 Q97.5

muunclear_typeabstract 2.5276332 1.177838 1.84638220 3.5087755

muunclear_typeFT 0.3160794 1.215287 0.21143058 0.4563983

muunclear_domain6 4.7198500 1.635976 1.92278936 13.3340176

muunclear_typeFT:domain6 0.9711313 1.791743 0.29675094 2.8956988

muhigh_typeabstract 0.2395456 1.319618 0.13684447 0.4019258

muhigh_typeFT 0.6568669 1.205234 0.45159364 0.9354653

muhigh_domain6 0.9766930 2.399918 0.15440248 4.8740969

muhigh_typeFT:domain6 0.1768507 2.891783 0.02137332 1.4676894

**Domain 7**

**Estimate Est.Error Q2.5 Q97.5**

**muunclear_typeabstract 4.1289180 1.195754 2.93970152 5.9127924**

**muunclear_typeFT 0.4539094 1.202709 0.31298622 0.6430708**

**muunclear_domain7 0.1365428 1.429628 0.06709504 0.2712983**

**muunclear_typeFT:domain7 5.6653384 1.673256 2.08690248 15.4158135**

**muhigh_typeabstract 0.2533978 1.353682 0.13741192 0.4501716**

**muhigh_typeFT 0.6893826 1.203716 0.47665610 0.9837350**

**muhigh_domain7 0.8690638 1.612946 0.33604104 2.1806985**

**muhigh_typeFT:domain7 0.2921315 1.919137 0.07916835 1.0263288**

# Ordinal model

**Domain 1**

**Estimate SE**

**elpd_waic -663.0 14.3**

**p_waic 25.3 0.9**

**waic 1326.1 28.7**

**Estimate Est.Error Q2.5 Q97.5**

**Intercept[1] 1.9176214 1.145242 1.4739967 2.5122045**

**Intercept[2] 14.5346631 1.187455 10.4259786 20.5311227**

**typeFT 6.4721150 1.184091 4.6605181 9.0221188**

**domain1 0.5268263 1.387599 0.2694799 0.9827573**

**typeFT:domain1 1.9047315 1.500903 0.8703904 4.2747135**

**Domain 2**

**Estimate SE**

**elpd_waic -656.6 14.3**

**p_waic 25.6 0.9**

**waic 1313.1 28.6**

**Estimate Est.Error Q2.5 Q97.5**

**Intercept[1] 1.8672011 1.143152 1.4413921 2.4378058**

**Intercept[2] 14.5106721 1.185200 10.5353113 20.3668119**

**typeFT 5.7933066 1.179166 4.2030843 8.0396420**

**domain2 0.4279931 1.401966 0.2143729 0.8081685**

**typeFT:domain2 4.7589770 1.525435 2.1196956 11.1184523**

**Domain 3**

**Estimate SE**

**elpd_waic -644.3 14.9**

**p_waic 26.7 1.0**

**waic 1288.6 29.8**

**Estimate Est.Error Q2.5 Q97.5**

**Intercept[1] 2.165935 1.149077 1.6450819 2.857935**

**Intercept[2] 18.119584 1.201922 12.6995299 26.273284**

**typeFT 6.138165 1.186211 4.4120193 8.630543**

**domain3 1.441694 1.350422 0.7946077 2.555728**

**typeFT:domain3 4.446753 1.508543 2.0036077 9.997029**

**Domain 4**

Estimate SE

elpd_waic -663.3 14.4

p_waic 25.3 0.9

waic 1326.7 28.8

Estimate Est.Error Q2.5 Q97.5

Intercept[1] 1.9259736 1.144119 1.4863988 2.526949

Intercept[2] 14.5890686 1.188812 10.4753264 20.805908

typeFT 6.3606607 1.185157 4.5761779 8.915688

domain4 0.5472001 1.388060 0.2864339 1.029591

typeFT:domain4 2.1550636 1.505060 0.9754451 4.822778

**Domain 5**

Estimate SE

elpd_waic -659.5 14.7

p_waic 24.7 0.9

waic 1319.1 29.3

Estimate Est.Error Q2.5 Q97.5

Intercept[1] 2.3401963 1.150389 1.7909227 3.0868728

Intercept[2] 17.9020488 1.194232 12.7669976 25.6497460

typeFT 8.3166053 1.193210 5.9035921 11.8471483

domain5 2.0503559 1.307993 1.2018192 3.4703513

typeFT:domain5 0.3692432 1.424593 0.1844016 0.7386776

**Domain 6**

Estimate SE

elpd_waic -639.7 15.0

p_waic 26.5 1.0

waic 1279.3 30.1

Estimate Est.Error Q2.5 Q97.5

Intercept[1] 1.7781735 1.147486 1.3627387 2.3305929

Intercept[2] 15.2274200 1.198278 10.7597747 21.8343503

typeFT 8.1083365 1.190848 5.7959376 11.4857683

domain6 0.2584984 1.462845 0.1215589 0.5270304

typeFT:domain6 0.6681817 1.577078 0.2783697 1.6555362

**Domain 7**

Estimate SE

elpd_waic -646.3 14.8

p_waic 25.1 1.0

waic 1292.5 29.6

Estimate Est.Error Q2.5 Q97.5

Intercept[1] 2.6398077 1.154447 1.99961253 3.5090193

Intercept[2] 20.9418668 1.201005 14.75226968 30.1589676

typeFT 9.7897423 1.193729 6.91613287 13.8454092

domain7 4.0332699 1.304396 2.38861174 6.7628363

typeFT:domain7 0.1594219 1.433427 0.07901759 0.3226259
